# Assessing the sustainability of reef and demersal fish stocks in Northwest México under a data-limited approach

**DOI:** 10.7717/peerj.21404

**Published:** 2026-07-02

**Authors:** Valerie Juárez-Vera, Georgina Ramírez-Ortiz, Fernando Aranceta-Garza, Felipe Amezcua

**Affiliations:** 1 Departamento de Ciencias de la Tierra, Facultad de Ciencias, Universidad Nacional Autónoma de México, Alcaldía Coyoacán, Ciudad de México, Mexico; 2 Instituto de Ciencias del Mar y Limnología, Universidad Nacional Autónoma de México, Mazatlán, Sinaloa, Mexico; 3 CONAHCYT, Centro de Investigaciones Biológicas Del Noroeste S.C., La Paz, Baja California Sur, Mexico

**Keywords:** Fisheries, Marine ecosystems, Catch-only stock management yield models, Resilience, Sustainable development goal 14

## Abstract

In México, most assessments have focused on industrial fisheries, while many finfish stocks remain underrepresented in regional evaluations. These information gaps present a significant challenge to achieving sustainability in artisanal fisheries, in addition to the complexities introduced by high species diversity and extensive social participation. This study aimed to evaluate the exploitation status of reef and demersal stocks reported in northwest México, dividing the region between the stocks of the Californian province (CAL) and the Cortez province (COR). Data-limited models (CMSY++) were employed to estimate relative stock size (B/B _MSY_) and exploitation rate (F/F_MSY_) for 111 stocks. Three exploitation approaches were considered to classify stocks as sustainable, not over-fished or unsustainable: (i) Flexible, (ii) Intermediate, and (iii) Conservative. Based on this classification, the proportion of sustainable stocks with respect to the total assessed was calculated (indicator 14.4.1, Sustainable Development Goal 14). Through this regional assessment, a low proportion of sustainable stocks were registered according to the three exploitation approaches considered: Flexible (CAL: 30%, COR: 26%), Intermediate (CAL: 11%, COR: 11%), and Conservative (CAL: 11%, COR: 9%), placing both provinces far from meeting the sustainable fisheries target of Sustainable Development Goal 14. These low proportions differ from what is reported globally, which might be due to the underrepresentation of reef and demersal stocks in fishery evaluations.

## Introduction

Sustainable fishing is defined as the extraction of resources based on the natural reproductive capacity of species, ensuring the good conservation status of ecosystems over time, as well as the protection of the livelihoods of coastal communities (Alho, Reis & Aquino, 2015; Macusi et al., 2023; Grati et al., 2025). According to the latest global assessment of marine fish stocks, 64.5% of stocks are exploited sustainably (whereas for the Eastern Central Pacific this proportion goes up to 69.2%), while 35.5% are exploited unsustainably (FAO, 2025a). This recent analysis indicates that the proportion of sustainable resources has remained stable compared to 2019 (64.6%) and 2017 (65.8%), showing a slight decrease in the percentage of sustainable stocks (FAO, 2025a). However, there is a lack of consensus regarding the reported status of fisheries at the regional and national levels, thus, global assessments might differ considerably depending on the estimates incorporated (Hilborn et al., 2020a; FAO, 2025a).

Since México is ranked 14th worldwide in fishery production, with the northwest region accounting for nearly 80% of the total catch (CONAPESCA, 2023), it is crucial to evaluate the state of its fishery resources to determine the long-term ecological, social, and economic viability of fishing activities (Lluch-Cota et al., 2006). Regarding multi-species stock assessments in México, Arreguín-Sánchez & Arcos-Huitrón (2011) reported that almost half (46.8%) were in decline, 46.3% were reported to be at maximum utilization, and 6.9% were classified as developing fisheries. At the regional level, the Gulf of California presented the lowest proportion of deteriorated stocks (41.6%), while the central Mexican Pacific had the highest (68.7%). In contrast, Giron-Nava et al. (2019a) conducted the first systematic assessment of artisanal fisheries (121 stocks) in the Gulf of California, finding that 69% of the stocks were unsustainable, 13% were classified as over-fished, 11% were subject to overfishing, while only 7% were sustainably fished.

Despite these multi-specific efforts, most stock assessments in México have been conducted on industrial fisheries, comprising high-value commercial species, namely crustaceans, tunas, and small pelagic fish (INAPESCA, 2016; Vallarta-Zárate et al., 2020; IATTC, 2024). In contrast, a high percentage of finfish stocks have not been evaluated (Sandoval-Ramírez et al., 2020), which represents a problem towards the sustainability of Mexican fisheries, since finfish are some of the primary resources captured by artisanal fisheries, along with sharks and mollusks (Ponce-Díaz et al., 2009).

Artisanal fisheries constitute approximately 97% of the fishing fleet in México, and produce 434.9 million dollars annually (2.5 times more than the income generated by industrial fleets), representing a source of employment and livelihood for the coastal communities (Seijo & Martínez, 2006; Ponce-Díaz et al., 2009; Martínez-Estrada et al., 2017). These characteristics highlight the need for their continuous monitoring.

In fishery resource sustainability, México has been engaged in various initiatives, such as the Agreement on Port State Measures (FAO, 2025b), the 2030 Agenda (Naciones Unidas México, 2025), and the 2025–2030 National Development Plan (Gobierno de México, 2025). To fulfill these commitments, it is essential to consider as many resources as possible in the national fishery assessments, including underrepresented fishery groups (*e.g.*, finfish). In this sense, Sustainable Development Goal 14 (SDG 14) of the 2030 Agenda establishes the guidelines for the conservation and sustainable use of oceans and marine resources, highlighting in its target 14.4 the effective regulation of fishing and its sustainable management (SEMARNAT, 2018; Constantino et al., 2022; Kuemlangan et al., 2023).

To assess compliance with this goal, ten indicators are employed, including indicator 14.4.1, which measures the proportion of biologically sustainable fish stocks (*i.e.,* stocks with abundance at Maximum Sustainable Yield or higher) relative to the total number of assessed stocks (FAO, 2019). For the evaluation of indicator 14.4.1, it is necessary to account for the Maximum Sustainable Yield (MSY), which is calculated using variables that indicate the fishery status, such as biomass (B), fishing pressure (F), fishing effort, and even economic rent (Haddon, 2011). In data-limited contexts, life history traits are essential for estimating stock status when biomass or fishing effort are unavailable (Fischer, De Oliveira & Kell, 2020). One of these traits is the intrinsic growth rate (*r*), closely linked to resilience, which is the capacity of a stock to remain viable facing environmental variation (Suatoni, Atkinson & Masterton, 2022). Species differ in their resilience based on life history traits, which influence how vulnerable fish stocks are to overexploitation (Musick, 1999). This understanding provides a baseline for categorizing species into resilience levels.

Despite the increase in biological information for commercial fish, it remains scarce for other species, thus, in the last decade, data-limited models have gained relevance for providing scientific information for decision-making, even in contexts of limited data (Scarcella et al., 2023). These models are used to estimate MSY and stock depletion levels (Feltrim & Dias, 2025), making them a key tool for assessing low-commercial-interest stocks when insufficient data preclude the use of more complex evaluations (Froese et al., 2023).

To interpret the results derived from these models, three main approaches have been employed to classify fisheries status (Giron-Nava et al., 2019a; Zhai, Liang & Pauly, 2020; Sharma et al., 2021). First, the Conservative approach (Kobe diagram) based on relative stock size (B/B_MSY_) and exploitation rate (F/*F*_MSY_) has been used to identify four exploitation states (Table 1): sustainable, unsustainable, overfishing (B/B_MSY_ and F/*F*_MSY_ > 1), and over-fished but not undergoing overfishing (B/B_MSY_ and F/*F*_MSY_ < 1; Maunder & Aires-da Silva, 2011). Through the diagram obtained with this approach, it is possible to visualize the historical trajectory of the stocks, and it is currently used by the Instituto Mexicano de Investigación en Pesca y Acuacultura Sustentables (IMIPAS) to report the status of fishing resources in México at the Carta Nacional Pesquera (CNP; Restrepo, 2019; INAPESCA, 2023).

Second, the Food and Agriculture Organization (FAO) approach, hereafter referred to as Intermediate approach, is based on identifying maximally sustainably fished, unsustainable, and underexploited stocks (Table 1; FAO, 2025a). This approach is used for reports on the status of global fishery resources, such as the State of World Fisheries and Aquaculture (SOFIA) report issued by the FAO biannually since 1997 (FAO, 2025a).

Third, the approach used by the National Oceanic and Atmospheric Administration of the United States of America (NOAA; Federal Register, 2016) and the Ministry of Fisheries in New Zealand (MFNZ, 2008), subsequently will be referred as the Flexible approach. This approach implies a less conservative overexploitation threshold (B/B_MSY_ ratio) than previous approaches (Conservative and Intermediate; Table 1), as it accounts for natural fluctuations in biomass levels because of environmental factors or species reproductive cycles (FAO, 2021).

**Table 1:** Biological reference points for stock status.

		**Approach**		
**Status**		**Flexible**	**Intermediate**	**Conservative**
Sustainable	Biomass	B/B_MSY_ > 0.8	B/B_MSY_ > 0.8 B/B_MSY_ > 1.2^*^	B/B_MSY_ > 1
	Catch	F/*F*_MSY_ < 1	F/*F*_MSY_ < 1	F/*F*_MSY_ < 1
Not over-fished	Biomass	B/B_MSY_ > 0.5		
	Catch	F/*F*_MSY_ < 1		
Unsustainable	Biomass	B/B_MSY_ < 0.5	B/B_MSY_ < 0.8	B/B_MSY_ < 1
	Catch	F/*F*_MSY_ > 1	F/*F*_MSY_ > 1	F/*F*_MSY_ > 1

**Notes.**

* Reference points defining a sustainable, unsustainable, not over-fished, and underexploited (*) stock according to the approaches considered in this study.

Under this Flexible approach, stocks with biomass levels below 0.5 B/B_MSY_ are considered to be over-fished, stocks with biomass above 0.8 are classified as sustainable, whereas those that fall between 0.5 and 0.8 represent an intermediate status which is not fully sustainable, but is not over-fished either (National Marine Fisheries Service, 2015; Federal Register, 2016). Using NOAA-based metrics such as the Fish Stock Sustainability Index (NOAA Fisheries, 2025), it is possible to measure the performance of fisheries, where stocks within the “not over-fished” category may still receive high scores due to adequate management status (up to three out of four points; NOAA Fisheries, 2025).

These three approaches are useful when a traditional assessment is not possible, since MSY reference points can be calculated with data-limited models like Catch-only Stock Management Yield (CMSY++; Froese et al., 2023). This model requires minimum parameters, such as catch series and resilience of each species, and can be complemented with estimates of biomass and/or Catch per Unit Effort (Froese et al., 2023).

Previous versions of the CMSY++ models (CMSY, CMSY+) have been used to conduct global and regional assessments, exhibiting a high proportion of unsustainable stocks (Froese et al., 2018; Zhai, Liang & Pauly, 2020; Sharma et al., 2021). At the global level, Sharma et al. (2021) reported that 75% of 48 stocks analyzed were found to be unsustainable under the Intermediate approach. In Europe, an assessment of 397 fish and invertebrate stocks found that 51% were unsustainable (Froese et al., 2018). Similarly, Zhai, Liang & Pauly (2020) assessed the status of 16 coastal fish stocks in China and reported that most were unsustainable. These stocks would also be considered as unsustainable under the Flexible approach, with a more restrictive threshold for sustainability (B ≥ B_MSY_) being applied in these studies (Froese et al., 2018; Zhai, Liang & Pauly, 2020).

Global reports on this matter may differ from regional reports due to the underrepresentation of certain groups in the evaluations in large-scale analyses. Based on all the above, in this work, it was hypothesized that the assessment of reef and demersal finfish in northwest México will reveal a high proportion of unsustainable stocks, resulting in low values for the 14.4.1 indicator (SDG 14), and positioning the most productive fishing region in the country far from achieving target 14.4. Accordingly, this study aimed to determine the exploitation status of reef and demersal finfish stocks in the Californian and Cortez provinces using three approaches with different sustainability thresholds (Flexible, Intermediate, and Conservative).

In addition, the study aimed to quantify the extent to which these stocks contribute to achieving SDG 14 target 14.4 in northwest México through the calculation of indicator 14.4.1. Similarly to FAO (2025a), which extended the criteria for “maximally sustainably fished” stocks from B ≥ B_MSY_ to B/B_MSY_ ≥ 0.8 for their global report of the indicator, an extension of the exploitation threshold for the Flexible approach was examined in this investigation, considering “not over-fished” stocks as sustainable. This assumption does not imply full biological sustainability, but rather serves as an operative exercise to assess how indicator 14.4.1 responds to less conservative thresholds.

## Materials and Methods

### Study area

Northwest México constitutes the region located between the Tropic of Cancer (23°N) to 32°N, comprising the states of Baja California, Baja California Sur, Sonora, and Sinaloa (Hernández-Cerda, Vidal-Zepeda & García, 1999). According to the marine biogeographic province classification by Robertson & Cramer (2009) for the Eastern Tropical Pacific (ETP), the Cortez province encompasses the Gulf of California and the Pacific coast of Baja California below 25°N. This region is distinguished by a marked environmental gradient, with a tropical climate in the south and a temperate climate in the far north (Robertson & Cramer, 2009). The combination of unique climatic and geographic conditions has made it a biodiversity hotspot with the confluence of temperate and tropical species, and the presence of many endemic species (Arellano-Peralta & Medrano-González, 2013).

**Figure 1:**
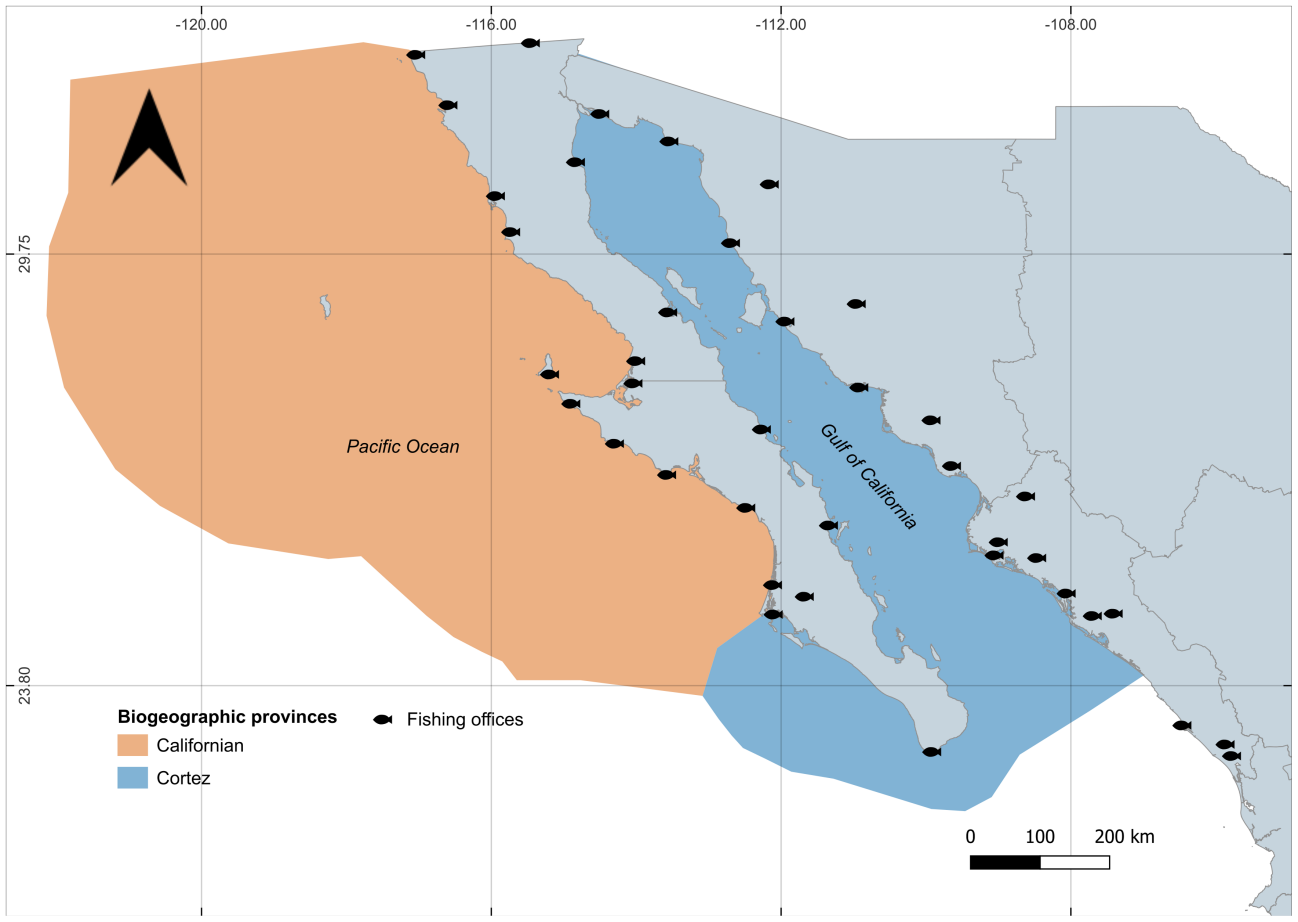
Map of the study area in northwest México. The fishing offices (fish markers) considered for the present analysis, along with their locations in the Cortez (blue) and Californian (orange) biogeographic provinces.

Likewise, the northwestern region of México includes a portion of the Californian province, which extends along the Pacific coast of the Baja California Peninsula above 25°N and reaches Southern California in the United States of America. This biogeographic province has a temperate climate, so species with affinities for cold water dominate its marine communities, although the presence of some tropical species has been reported (Robertson & Cramer, 2009). Based on this classification by Robertson & Cramer (2009), the reef and demersal finfish stocks of northwest México were divided for analysis into the Californian (CAL) and the Cortez (COR) provinces (Fig. 1).

### Data-limited models

To assess the status of fisheries exploitation in northwest México, catch data (in tonnes) of reef and demersal species were collected from catch reports at 41 fisheries offices located in the states of Baja California, Baja California Sur, Sonora, and Sinaloa for the period 2006–2023. The data were generated by the Comisión Nacional de Acuacultura y Pesca and provided through dataMares (Mascareñas Osorio, Aburto-Oropeza & Sánchez-Ortiz, 2018). Besides the total catch, the database included information on the year, month, state name and code, fishery office name and code, common and scientific names of the captured species, and monetary value.

From 325 stocks registered for northwest México, those that met two criteria were selected: that the species were associated with reefs or soft bottoms, and that they had a minimum of 15 years of catch records, which is essential to carry out the CMSY++ models (FAO, 2019). For those stocks that presented gaps in catch records, the mean values of adjacent years were calculated following Froese et al. (2021).

A review of the scientific names of the species reported in the catch records was conducted based on their geographic distribution (Robertson et al., 2024; Froese & Pauly, 2025; CONABIO, 2025). For those records in which a species did not meet the 15-year data requirement or it was not possible to determine the identity at the species level, records of the same Genus were grouped into multi-specific stocks (18 out of 111 stocks analyzed). The area abbreviation was added as an identifier (*e.g.*, *Mugil spp*. CAL, *Mugil spp*. COR) to distinguish stocks present in both provinces but composed of different species.

For each stock, the species resilience level was obtained from FishBase (Froese & Pauly, 2025). For multi-specific stocks, the most prevalent resilience level among the species comprising each stock was used as a homogenization criterion to address potential differences in life-history traits. When no clear majority of resilience levels was present in multi-specific stocks, we followed a precautionary approach and selected the lowest level.

Resilience was incorporated into CMSY++ models through intrinsic growth rate intervals (*r*), serving as a biological proxy for fish stock productivity. These intervals were proposed by the American Fisheries Society (Musick, 1999) and adapted for FishBase (Froese et al., 2017), ranging from high (0.6–1.5), medium (0.2–0.8), low (0.05–0.5), to very low (0.015–0.1) resilience level for each species.

Based on catch records (in tonnes) and resilience levels obtained from FishBase (Froese & Pauly, 2025), CMSY++ models (Froese et al., 2023) were run to estimate MSY reference points: relative stock size and exploitation rate (B/B_MSY_ and F/*F*_MSY_). Parameter estimation using CMSY++ models are given within a Bayesian framework, and intrinsic growth rate (*r*), carrying capacity (K) and MSY, are assumed to follow lognormal distributions as negative values are biologically implausible (Froese et al., 2023). While *r* is bounded by life-history traits (*i.e.,* resilience), MSY is derived from the catch series, specifically using the maximum catch as an upper bound. Based on these priors and the Schaefer surplus model, K is parameterized as K_prior_ = 4 MSY_prior_/*r*_prior_ (Froese et al., 2023).

One advantage of CMSY++ models over other data-limited models, is that they are based on artificial neural networks trained on information from 400 fish and invertebrate stocks with typical exploitation patterns, allowing the objective prediction of relative stock size (Sea Around Us, 2023). This implementation of artificial intelligence in model parameterization is adequate in regions where biomass estimates are unavailable (Froese et al., 2023). Another key improvement of CMSY++ models is the use of a multivariate lognormal prior that explicitly accounts for the inverse correlation between *r* and K, and departs from the assumption in CMSY models that all combinations of *r* and K are equally probable (Froese et al., 2023).

Using the CMSY++ models, time series of annual catch, fishing pressure (F), and estimated biomass (B) were obtained. The outputs used for this analysis were: (1) estimates of relative stock size (B/B_MSY_) and exploitation rate (F/*F*_MSY_), and (2) Kobe plots to visualize temporal changes in the relationship between these two estimates. These plots (provided in the Supplemental material) include confidence ellipses generated by CMSY++ models representing uncertainty at 50%, 80%, and 95% (Froese et al., 2021). The code used for this analysis was modified from Froese et al. (2023). The modified code, along with the files required to generate CMSY++ models are available on the GitHub repository: (https://github.com/lecofym/Sustainability_Mexican_Finfish_Stocks).

### Exploitation approaches

**Figure 2:**
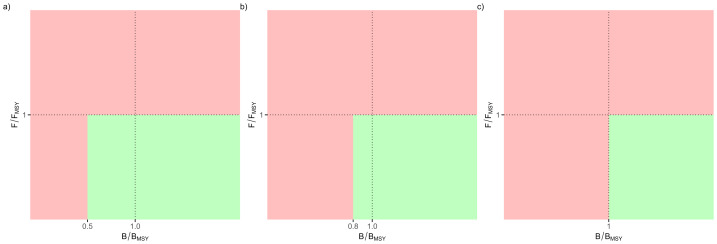
Exploitation status of fishery resources. Graphical representation showing sustainable (green) and unsustainable (red) status, based on the relationship between the relative size of the stock (B/B_MSY_) and the exploitation rate (F/F_MSY_) relative to the Maximum Sustainable Yield (MSY). The exploitation thresholds for the three approaches considered are as follows: (A) Flexible approach (National Marine Fisheries Service, 2015; Federal Register, 2016), (B) Intermediate approach (FAO, 2025a), and (C) Conservative approach (Joint Meeting of Tuna RFMOs, 2007).

For this study, stock status was classified using approaches with different exploitation thresholds (Table 1; Fig. 2). Based on the results for each approach, stocks were grouped as follows:

1. Sustainable stocks under the three approaches.
2. Sustainable stocks only under the Flexible and Intermediate approaches.
3. Not over-fished stocks under the Flexible approach.
4. Unsustainable stocks according to the three approaches.

Cochran’s Q test was applied to evaluate significant differences among the number of sustainable and unsustainable stocks in each province (Aslam, 2023). This test was performed using the package ‘DescTools’ (Signorell, 2025) in R v 4.5.1 (R Core Team, 2025a). When significant differences were detected, McNemar’s test from the package ‘stats’ (R Core Team, 2025b) was applied to determine which paired approaches differed significantly in their classifications (Pembury-Smith & Ruxton, 2020).

### Calculation of indicator 14.4.1

With the information obtained for the three approaches in each biogeographic province, the proportion of biologically sustainable stocks relative to the total number of stocks assessed was calculated (Eq. (1); FAO, 2024):

(1) \begin{eqnarray*}{P}_{s}= \frac{{N}_{S}}{N} \times 100= \frac{{N}_{S}}{{N}_{S}+{N}_{u}} \times 100\end{eqnarray*}

where *P*_*s*_ is the percentage of stocks classified as sustainable calculated for each biogeographic province and for each approach, *N*_*S*_ is the number of stocks classified as sustainable, *N*_*u*_ is the number of stocks classified as unsustainable, and *N* = *N*_*S*_ + *N*_*u*_ corresponds to the total stocks analyzed for each region.

By calculating indicator 14.4.1 of SDG 14, it was possible to evaluate the progress of the biogeographic provinces towards achieving target 14.4, where high values indicated a high proportion of sustainable stocks in the assessed province. In contrast, low values indicated that most stocks were under overexploitation and/or overfishing conditions, so the analyzed biogeographic province was far from achieving target 14.4 (Table 2; FAO, 2023).

**Table 2:** Values of Sustainable Development Goal (SDG) indicator 14.4.1. The intervals are used to define the distance to the achievement of SDG 14 target 14.4 (sustainable fisheries), as reported by FAO (2023).

**Indicator interval** ** 14.4.1**	**Interpretation**
indicator ≤ 0.25	Very far from achieving the goal.
0.25 < indicator ≤ 0.50	Far from achieving the goal.
0.50 < indicator ≤ 0.75	Moderate distance to reach the goal.
0.75 < indicator < 1	Close to reaching the goal.
indicator = 1	Goal accomplished.

## Results

Based on the selection criteria (reef/demersal finfish species with at least 15 years of catch records), 111 stocks (93 single-species stocks and 18 multi-species stocks) were considered for this study. Estimated biomass (B) and fishing pressure (F) values relative to MSY (B/B_MSY_, F/*F*_MSY_), were obtained from the CMSY++ models for the period 2006–2023 in northwest México. Based on the exploitation thresholds resulting from each approach, 28% of stocks were sustainable according to the Flexible approach, 11% according to the Intermediate approach, and 10% according to the Conservative approach. The 111 stocks analyzed had resilience levels ranging from very low to high (0.015 < *r* < 1.5; Froese & Pauly, 2025).

### Californian Province (CAL)

For the Californian Province (CAL), 46 stocks were evaluated and were grouped based on their exploitation status under the three approaches: group 1, classified as sustainable according to the three approaches, was integrated by five stocks with low to medium resilience levels (0.015 < *r* < 0.8; *Brotula spp.*, *Caulolatilus affinis*, *Diapterus spp*., *Mycteroperca rosacea* and *Trachinotus paitensis*; Table 3; Fig. S1). Within this group, *T. paitensis*, was particularly salient, displaying the highest biomass estimate relative to MSY (B/B_MSY_ = 1.259; Fig. S1E) of the analyzed species in this province, indicating that it is an underexploited stock.

**Table 3:** Results of Catch-only Stock Management Yield models (CMSY++) for reef and demersal fish stocks in the Californian province. Results for 46 reef and demersal fish stocks (with stock and species codes) reported to fisheries offices in the Californian province. The resilience level (intervals of intrinsic growth rate), relative stock size (B/B_MSY_), exploitation rate (F/F_MSY_), exploitation status (+ for sustainable and * for unsustainable), and species that integrate the stocks are presented for each identified group. At the end of the table, the proportion of sustainable and unsustainable stocks according to each approach (Flexible, Intermediate, and Conservative), in addition to the indicator 14.4.1 value for the Californian province are showed.

**Stock**	**Code**	**Resilience**	**B/B_MSY_**	**F/F_MSY_**	**Flexible**	**Intermediate**	**Conservative**	**Species**
**Group 1**								
*Brotula spp.*	*Bro*	0.2–0.8	1.174	0.48	+	+	+	*Brotula spp.*
*Caulolatilus affinis*	*Caf*	0.05–0.015	1.125	0.406	+	+	+	*Caulolatilus affinis*
*Diapterus spp.*	*Dia*	0.2–0.8	1.18	0.544	+	+	+	*Diapterus peruvianus, Diapterus auratus*
*Mycteroperca rosacea*	*Mro*	0.05–0.015	1.149	0.707	+	+	+	*Mycteroperca rosacea*
*Trachinotus paitensis*	*Tpa*	0.2–0.8	1.259	0.535	+	+	+	*Trachinotus paitensis*
**Group 3**								
*Anisotremus interruptus*	*Ain*	0.05–0.015	0.544	0.77	+	*	*	*Anisotremus interruptus*
*Balistes polylepis*	*Bpo*	0.05–0.015	0.561	0.379	+	*	*	*Balistes polylepis*
*Centropomus spp. CAL*	*CenCAL*	0.2–0.8	0.768	0.97	+	*	*	*Centropomus spp., Centropomus undecimalis*
*Hyporthodus niphobles*	*Hni*	0.2–0.8	0.521	0.845	+	*	*	*Hyporthodus niphobles*
*Menticirrhus elongatus*	*Mel*	0.2–0.8	0.569	0.66	+	*	*	*Menticirrhus elongatus*
*Paralabrax nebulifer*	*Pne*	0.2–0.8	0.654	0.885	+	*	*	*Paralabrax nebulifer*
*Paralabrax spp.*	*Par*	0.05–0.015	0.572	0.58	+	*	*	*Paralabrax auroguttatus, Paralabrax maculatofasciatus*
*Scorpaena mystes*	*Smy*	0.05–0.015	0.683	0.649	+	*	*	*Scorpaena mystes*
*Trachinotus spp. CAL*	*TraCAL*	0.2–0.8	0.522	0.852	+	*	*	*Trachinotus spp., Trachinotus rhodopus*
**Group 4**								
*Bagre panamensis*	*Bpa*	0.2–0.8	0.466	0.654	*	*	*	*Bagre panamensis*
*Bodianus spp.*	*Bod*	0.015–0.1	0.432	0.362	*	*	*	*Bodianus spp.*
*Caulolatilus princeps*	*Cpr*	0.05–0.015	0.185	0.184	*	*	*	*Caulolatilus princeps*
*Cynoscion spp. CAL*	*CynCAL*	0.05–0.015	0.277	0.125	*	*	*	*Cynoscion spp., Cynoscion nothus, Cynoscion othonopterus, Cynoscion reticulatus, Cynoscion parvipinnis, Cynoscion nebulosus*
*Diplectrum euryplectrum*	*Deu*	0.2–0.8	0.237	0.275	*	*	*	*Diplectrum euryplectrum*
*Eucinostomus spp.*	*Euc*	0.2–0.8	0.214	0.621	*	*	*	*Eucinostomus spp., Eucinostomus argenteus, Eucinostomus gracilis, Eucinostomus gula, Eucinostomus lefroyi*
*Haemulon spp.*	*Hae*	0.05–0.015	0.403	0.75	*	*	*	*Haemulon spp.*
*Hoplopagrus guentherii*	*Hgu*	0.05–0.015	0.267	0.301	*	*	*	*Hoplopagrus guentherii*
*Hyporthodus acanthistius*	*Hac*	0.015–0.1	0.285	0.039	*	*	*	*Hyporthodus acanthistius*
*Kyphosus spp.*	*Kyp*	0.2–0.8	0.217	0.194	*	*	*	*Kyphosus spp.*
*Lutjanus argentiventris*	*Lar*	0.05–0.015	0.281	0.161	*	*	*	*Lutjanus argentiventris*
*Lutjanus colorado*	*Lco*	0.05–0.015	0.266	0.036	*	*	*	*Lutjanus colorado*
*Lutjanus novemfasciatus*	*Lno*	0.015–0.1	0.302	0.04	*	*	*	*Lutjanus novemfasciatus*
*Lutjanus spp.*	*Lut*	0.2–0.8	0.199	0.509	*	*	*	*Lutjanus spp.*
*Menticirrhus spp.*	*Men*	0.2–0.8	0.266	0.501	*	*	*	*Menticirrhus spp.*
*Microlepidotus inornatus*	*Min*	0.2–0.8	0.229	0.425	*	*	*	*Microlepidotus inornatus*
*Micropogonias megalops*	*Mme*	0.2–0.8	0.444	0.742	*	*	*	*Micropogonias megalops*
*Mugil spp. CAL*	*MugCAL*	0.2–0.8	0.413	0.671	*	*	*	*Mugil spp., Mugil hospes, Mugil curema, Mugil setosus*
*Scarus spp. CAL*	*ScaCAL*	0.2–0.8	0.453	0.715	*	*	*	*Scarus spp., Scarus perrico*
*Selar crumenophthalmus*	*Scr*	0.6–1.5	0.191	0.372	*	*	*	*Selar crumenophthalmus*
*Calamus spp.*	*Cal*	0.2–0.8	0.471	1.056	*	*	*	*Calamus brachysomus, Calamus bajonado, Calamus pennatula*
*Cephalopholis colonus*	*Cco*	0.2–0.8	0.323	1.003	*	*	*	*Cephalopholis colonus*
*Cyclopsetta spp.*	*Cyc*	0.2–0.8	0.514	1.074	*	*	*	*Cyclopsetta spp.*
*Diplectrum pacificum*	*Dpa*	0.2–0.8	0.669	1.067	*	*	*	*Diplectrum pacificum*
*Epinephelus analogus*	*Ean*	0.2–0.8	0.693	1.628	*	*	*	*Epinephelus analogus*
*Epinephelus spp. CAL*	*EpiCAL*	0.2–0.8	0.274	1.279	*	*	*	*Epinephelus spp.*
*Lutjanus guttatus*	*Lgu*	0.2–0.8	0.258	1.067	*	*	*	*Lutjanus guttatus*
*Lutjanus peru*	*Lpe*	0.2–0.8	0.643	1.109	*	*	*	*Lutjanus peru*
*Mycteroperca prionura*	*Mpr*	0.015–0.1	0.537	1.104	*	*	*	*Mycteroperca prionura*
*Sebastes spp.*	*Seb*	0.015–0.1	0.82	1.152	*	*	*	*Sebastes spp.*
*Selene brevoortii*	*Sbr*	0.6–1.5	0.275	1.262	*	*	*	*Selene brevoortii*
*Sphoeroides annulatus*	*San*	0.2–0.8	0.58	1.057	*	*	*	*Sphoeroides annulatus*
Sustainable stocks					14	5	5	
Unsustainable stocks					32	41	41	
Indicator 14.4.1					0.3	0.11	0.11	

For group 2 (sustainable stocks only under the Flexible and Intermediate approaches), no stocks were registered within the Californian province. Group 3 presented nine stocks that were sustainable according to the Flexible approach and unsustainable for the Intermediate and Conservative approaches. This group comprised stocks with low to medium resilience levels (0.015 < *r* < 0.8; Table 3), such as *Anisotremus interruptus*, *Balistes polylepis*, *Centropomus spp*. CAL, *Hyporthodus niphobles*, *Menticirrhus elongatus*, *Paralabrax nebulifer*, *Paralabrax spp*., *Scorpaena mystes*, and *Trachinotus spp*. CAL (Fig. S2).

**Figure 3:**
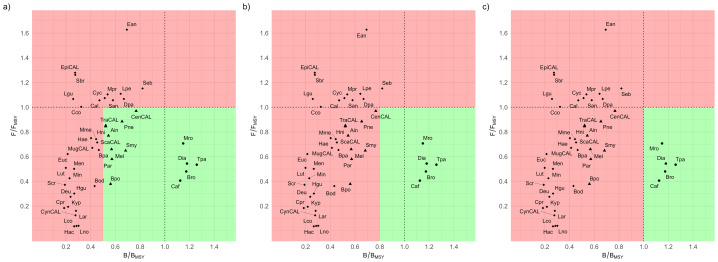
Graphical representation of the exploitation status of 46 reef and demersal fish stocks in the Californian province up to 2023. Each stock is located at a sustainable (green) or unsustainable (red) status according to the approach considered: (A) Flexible, (B) Intermediate, and (C) Conservative. The grouping according to the results of the three approaches is also presented: group 1 (circle) with sustainable stocks for the three approaches, group 3 (triangle) with not over-fished stocks under the Flexible approach, and group 4 (diamond) integrated by unsustainable stocks in all the approaches considered in this study.

Group 4 was the largest in this province, comprising 32 stocks (Fig. S3) that presented unsustainable status under the three approaches (Table 3). Within this group, 20 stocks had the lowest biomass values in the study region (B/B_MSY_ = 0.3 on average; Figs. S3A–S3T), with stocks such as *Caulolatilus princeps, Lutjanus spp.,* and *Selar crumenophthalmus* reaching severe overexploitation levels (B/B_MSY_ < 0.2). In terms of resilience, most stocks in group 4 showed medium levels (0.2 < *r* < 0.8). Figure 3 presents a visual summary of the exploitation status for the 46 stocks reported in the Californian province derived from model estimates based on data up to 2023, where the lowest proportion of stocks belonged to group 1 (five stocks, circles), there was no representation for group 2, group 3 showed an intermediate proportion of stocks (nine stocks, triangles), while the highest proportion of stocks integrated the group 4 (32 stocks, diamonds).

Cochran’s Q test and McNemar’s test were applied to evaluate significant differences in the number of sustainable and unsustainable stocks between each paired approach for the Californian province. Through these tests, significant differences were found (*Q* = 18, *df* = 2, *p* < 0.001) between the Flexible approach and the Intermediate and Conservative approaches (Table 4). Since no discordant cases were observed between Intermediate and Conservative approaches (which are needed to apply McNemar’s test), this indicated complete agreement in their sustainability classifications.

**Table 4:** McNemar’s test results for paired exploitation approaches in the Californian province. The *χ*^2^ values, *p*-values (*p* < 0.05 showing significant differences), and degrees of freedom (df) are presented.

	**Flexible *vs* Intermediate**	**Flexible *vs* Conservative**	**Intermediate *vs* Conservative**
*χ* ^2^	7.1	7.1	–
*p-* value	0.007	0.007	–
df	1	1	1

Based on the classification by approach, indicator 14.4.1 was calculated to assess the Californian province toward achieving the SDG 14 sustainable fisheries target (14.4; Table 3). According to the Flexible approach, 30% of stocks were sustainable, while for the Intermediate and Conservative approach, the same percentage of sustainable stocks was observed (11%). These results (indicator < 0.25) suggest that this province is very far from achieving target 14.4 (Table 2).

### Cortez Province (COR)

For the Cortez Province (COR), 65 stocks were evaluated, and group 1 (sustainable under the three approaches) was composed of six stocks with low to medium resilience levels (0.015 < *r* < 0.8): *Aluterus scriptus*, *Brotula spp.*, *Centropomus spp.* COR, *Cynoscion xanthulus*, *Diplectrum pacificum,* and *Mycteroperca rosacea* (Table 5; Fig. S4). Among these, *D. pacificum* (B/B_MSY_ = 1.350), *A. scriptus* (B/B_MSY_ = 1.297), and *Brotula spp.* (B/B_MSY_ = 1.278) were underexploited. Group 2 in this province was represented only by *Mycteroperca jordani* (Fig. S5), a species with very low resilience (*r* < 0.05; Table 5).

**Table 5:** Results of Catch-only Stock Management Yield models (CMSY++) for reef and demersal fish stocks in the Cortez province. Results for 65 reef and demersal fish stocks (with stock and species codes) reported to fisheries offices in the Cortez province. The resilience level (intervals of intrinsic growth rate), relative stock size (B/B_MSY_), exploitation rate (F/F_MSY_), exploitation status (+ for sustainable and * for unsustainable), and species that integrate the stocks are presented for each identified group. At the end of the table, the proportion of sustainable and unsustainable stocks according to each approach (Flexible, Intermediate, and Conservative), in addition to the indicator 14.4.1 value for the Cortez province are showed.

**Stock**	**Code**	**Resilience**	**B/B_MSY_**	**F/F_MSY_**	**Flexible**	**Intermediate**	**Conservative**	**Species**
**Group 1**								
*Aluterus scriptus*	*Asc*	0.2–0.8	1.297	0.569	+	+	+	*Aluterus scriptus*
*Brotula spp.*	*Bro*	0.2–0.8	1.278	0.537	+	+	+	*Brotula spp.*
*Centropomus spp. COR*	*CenCOR*	0.2–0.8	1.126	0.816	+	+	+	*Centropomus spp., Centropomus undecimalis, Centropomus robalito*
*Cynoscion xanthulus*	*Cxa*	0.05–0.015	1.05	0.539	+	+	+	*Cynoscion xanthulus*
*Diplectrum pacificum*	*Dpa*	0.2–0.8	1.35	0.525	+	+	+	*Diplectrum pacificum*
*Mycteroperca rosacea*	*Mro*	0.05–0.015	1.105	0.43	+	+	+	*Mycteroperca rosacea*
**Group 2**								
*Mycteroperca jordani*	*Mjo*	0.015–0.1	0.928	0.621	+	+	*	*Mycteroperca jordani*
**Group 3**								
*Calamus spp.*	*Cal*	0.2–0.8	0.662	0.896	+	*	*	*Calamus brachysomus, Calamus bajonado, Calamus pennatula*
*Caulolatilus princeps*	*Cpr*	0.05–0.015	0.73	0.995	+	*	*	*Caulolatilus princeps*
*Diapterus spp.*	*Dia*	0.2–0.8	0.695	0.896	+	*	*	*Diapterus peruvianus, Diapterus auratus*
*Eucinostomus spp.*	*Euc*	0.2–0.8	0.551	0.802	+	*	*	*Eucinostomus spp., Eucinostomus argenteus, Eucinostomus gracilis, Eucinostomus gula, Eucinostomus lefroyi*
*Eugerres lineatus*	*Eli*	0.6–1.5	0.707	0.867	+	*	*	*Eugerres lineatus*
*Haemulon spp.*	*Hae*	0.05–0.015	0.638	0.866	+	*	*	*Haemulon spp.*
*Mugil spp. COR*	*MugCOR*	0.2–0.8	0.563	0.984	+	*	*	*Mugil spp., Mugil hospes*
*Synodus spp.*	*Syn*	0.6–1.5	0.701	0.768	+	*	*	*Synodus spp.*
*Trachinotus paitensis*	*Tpa*	0.2–0.8	0.568	0.98	+	*	*	*Trachinotus paitensis*
*Trachinotus rhodopus*	*Trh*	0.2–0.8	0.525	0.783	+	*	*	*Trachinotus rhodopus*
**Group 4**								
*Bagre panamensis*	*Bpa*	0.2–0.8	0.255	0.518	*	*	*	*Bagre panamensis*
*Balistes polylepis*	*Bpo*	0.05–0.015	0.319	0.409	*	*	*	*Balistes polylepis*
*Bodianus spp.*	*Bod*	0.015–0.1	0.479	0.598	*	*	*	*Bodianus spp.*
*Caulolatilus affinis*	*Caf*	0.05–0.015	0.488	0.368	*	*	*	*Caulolatilus affinis*
*Centropomus medius*	*Cme*	0.2–0.8	0.43	0.669	*	*	*	*Centropomus medius*
*Cephalopholis colonus*	*Cco*	0.2–0.8	0.238	0.588	*	*	*	*Cephalopholis colonus*
*Cynoscion reticulatus*	*Cre*	0.2–0.8	0.205	0.367	*	*	*	*Cynoscion reticulatus*
*Cynoscion spp. COR*	*CynCOR*	0.05–0.015	0.215	0.063	*	*	*	*Cynoscion spp., Cynoscion nothus, Cynoscion othonopterus, Cynoscion stolzmanni, Cynoscion parvipinnis, Cynoscion nebulosus*
*Hoplopagrus guentherii*	*Hgu*	0.05–0.015	0.357	0.49	*	*	*	*Hoplopagrus guentherii*
*Hyporthodus acanthistius*	*Hac*	0.015–0.1	0.312	0.112	*	*	*	*Hyporthodus acanthistius*
*Kathetostoma averruncus*	*Kav*	0.2–0.8	0.321	0.756	*	*	*	*Kathetostoma averruncus*
*Kyphosus spp.*	*Kyp*	0.2–0.8	0.331	0.968	*	*	*	*Kyphosus spp.*
*Lutjanus argentiventris*	*Lar*	0.05–0.015	0.32	0.266	*	*	*	*Lutjanus argentiventris*
*Lutjanus colorado*	*Lco*	0.05–0.015	0.304	0.314	*	*	*	*Lutjanus colorado*
*Lutjanus guttatus*	*Lgu*	0.2–0.8	0.25	0.798	*	*	*	*Lutjanus guttatus*
*Lutjanus novemfasciatus*	*Lno*	0.015–0.1	0.318	0.165	*	*	*	*Lutjanus novemfasciatus*
*Lutjanus peru*	*Lpe*	0.2–0.8	0.248	0.66	*	*	*	*Lutjanus peru*
*Menticirrhus spp.*	*Men*	0.2–0.8	0.224	0.705	*	*	*	*Menticirrhus spp.*
*Microlepidotus inornatus*	*Min*	0.2–0.8	0.228	0.203	*	*	*	*Microlepidotus inornatus*
*Micropogonias megalops*	*Mme*	0.2–0.8	0.225	0.168	*	*	*	*Micropogonias megalops*
*Mugil curema*	*Mcu*	0.2–0.8	0.3	0.934	*	*	*	*Mugil curema*
*Mulloidichthys dentatus*	*Mde*	0.2–0.8	0.287	0.193	*	*	*	*Mulloidichthys dentatus*
*Nematistius pectoralis*	*Npe*	0.2–0.8	0.364	0.716	*	*	*	*Nematistius pectoralis*
*Paralabrax nebulifer*	*Pne*	0.2–0.8	0.213	0.172	*	*	*	*Paralabrax nebulifer*
*Paralabrax spp.*	*Par*	0.05–0.015	0.317	0.31	*	*	*	*Paralabrax auroguttatus, Paralabrax maculatofasciatus*
*Scarus spp. COR*	*ScaCOR*	0.2–0.8	0.277	0.611	*	*	*	*Scarus spp.*
*Scorpaena mystes*	*Smy*	0.05–0.015	0.468	0.503	*	*	*	*Scorpaena mystes*
*Seriola spp.*	*Ser*	0.05–0.015	0.296	0.47	*	*	*	*Seriola lalandi,Seriola dumerili*
*Anisotremus interruptus*	*Ain*	0.05–0.015	0.772	1.249	*	*	*	*Anisotremus interruptus*
*Atractoscion nobilis*	*Ano*	0.05–0.015	0.357	1.045	*	*	*	*Atractoscion nobilis*
*Bagre pinnimaculatus*	*Bpi*	0.05–0.015	0.576	1.176	*	*	*	*Bagre pinnimaculatus*
*Chanos chanos*	*Cch*	0.05–0.015	0.503	1.153	*	*	*	*Chanos chanos*
*Cyclopsetta spp.*	*Cyc*	0.2–0.8	0.634	1.181	*	*	*	*Cyclopsetta spp.*
*Diplectrum euryplectrum*	*Deu*	0.2–0.8	0.298	1.257	*	*	*	*Diplectrum euryplectrum*
*Elops affinis*	*Eaf*	0.2–0.8	0.592	1.143	*	*	*	*Elops affinis*
*Epinephelus spp. COR*	*EpiCOR*	0.2–0.8	0.685	1.818	*	*	*	*Epinephelus spp., Epinephelus analogus*
*Hyporthodus niphobles*	*Hni*	0.2–0.8	0.696	1.133	*	*	*	*Hyporthodus niphobles*
*Lutjanus spp.*	*Lut*	0.2–0.8	0.425	1.094	*	*	*	*Lutjanus spp.*
*Menticirrhus elongatus*	*Mel*	0.2–0.8	0.69	1.477	*	*	*	*Menticirrhus elongatus*
*Mugil setosus*	*Mse*	0.2–0.8	0.551	1.038	*	*	*	*Mugil setosus*
*Mycteroperca prionura*	*Mpr*	0.015–0.1	0.566	1.493	*	*	*	*Mycteroperca prionura*
*Scarus perrico*	*Spe*	0.2–0.8	0.698	1.277	*	*	*	*Scarus perrico*
*Sebastes spp.*	*Seb*	0.015–0.1	0.628	1.052	*	*	*	*Sebastes spp.*
*Selar crumenophthalmus*	*Scr*	0.6–1.5	0.419	1.613	*	*	*	*Selar crumenophthalmus*
*Selene brevoortii*	*Sbr*	0.6–1.5	0.432	1.08	*	*	*	*Selene brevoortii*
*Sphoeroides annulatus*	*San*	0.2–0.8	0.412	1.114	*	*	*	*Sphoeroides annulatus*
*Stereolepis gigas*	*Sgi*	0.015–0.1	0.484	1.435	*	*	*	*Stereolepis gigas*
*Trachinotus spp. COR*	*TraCOR*	0.2–0.8	0.259	1.413	*	*	*	*Trachinotus spp.*
Sustainable stocks					17	7	6	
Unsustainable stocks					48	58	59	
**Indicator 14.4.1**					0.26	0.11	0.09	

Group 3 was composed of ten sustainable stocks under the Flexible approach such as *Calamus spp*., *Diapterus spp., Eucinostomus spp.*, *Mugil spp*. COR, *Trachinotus paitensis*, and *Trachinotus rhodopus* (Fig. S6)*.* Most of the stocks in this group showed medium resilience levels (0.2 < *r* < 0.8; Table 5).

**Figure 4:**
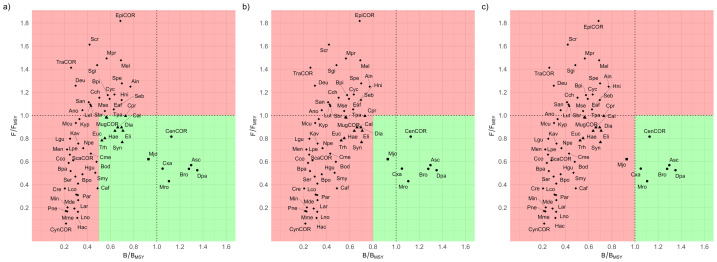
Graphical representation of the exploitation status of 65 reef and demersal fish stocks in the Cortez province up to 2023. Each stock is located at a sustainable (green) or unsustainable (red) status according to the approach considered: (A) Flexible, (B) Intermediate, and (C) Conservative. The grouping according to the results of the three approaches is also presented: group 1 (circles) with sustainable stocks for the three approaches, group 2 (square) with sustainable stocks under the Flexible and Intermediate approaches, group 3 (triangle) with not over-fished stocks according to the Flexible approach, and group 4 (diamond) integrated by unsustainable stocks for all the approaches.

Similarly to the Californian province, in the Cortez province group 4 was the largest, with 48 stocks classified as unsustainable under all the approaches (Table 5; Fig. S7). Most of the stocks in this group displayed medium (0.16 < *r* < 0.5; 27 stocks) and low (0.015 < *r* < 0.05; 13 stocks) resilience levels. It is worth mentioning that 20 stocks in this group were undergoing overfishing (F/*F*_MSY_ > 1) and overexploitation (B/B_MSY_ < 1; Figs. S7cc–S7vv), however, no stocks were identified under conditions of severe overexploitation (B/B_MSY_ < 0.2). The proportion of stocks in group 1 (six stocks, circles) and group 2 (one stock, square) was the lowest, while group 3 (10 stocks, triangles) exhibited an intermediate proportion. The highest proportion of the analyzed stocks was in group 4 (48 stocks, diamonds; Fig. 4).

Cochran’s Q test revealed significant differences among approaches in the Cortez province (*Q* = 20.182, *df* = 2, *p* < 0.001). Similar to the Californian province, McNemar’s test showed significant differences between the Flexible approach and the other approaches considered in this study (Intermediate and Conservative; Table 6).

**Table 6:** McNemar’s test results for paired exploitation approaches in the Cortez province. The *χ*^2^ values, p-values (*p* < 0.05 showing significant differences), and degrees of freedom (df) are presented.

	**Flexible *vs* Intermediate**	**Flexible *vs* Conservative**	**Intermediate *vs* Conservative**
*χ* ^2^	8.1	9.09	0
*p-* value	0.004	0.002	1
df	1	1	1

Based on the classification for each approach, indicator 14.4.1 was calculated to measure the success of the Cortez province toward achieving the sustainable fisheries target of SDG 14 (14.4; Table 5). Under the Flexible approach, 26% of stocks were found to be sustainable (consisting of fully sustainable and not over-fished stocks), reflecting that this region is far from meeting target 14.4 (Table 2). Under the Intermediate approach, 11% of stocks were found to be sustainable, while under the Conservative approach, it was 9%, the lowest percentage obtained for the two analyzed biogeographic provinces. Both results imply that the Cortez province is very far from meeting target 14.4.

Californian and Cortez provinces showed similar proportions of sustainable stocks under the three approaches: Flexible (CAL = 30%; COR = 26%), Intermediate (CAL = 11%; COR = 11%), and Conservative (CAL = 11%; COR = 9%). However, the Californian province had a higher percentage of sustainable stocks under the Flexible and Conservative approaches than Cortez province.

## Discussion

Based on the results of this analysis, it can be asserted that most reef and demersal stocks analyzed in the Californian and Cortez provinces showed signs of an unsustainable state of exploitation. This coincides with the results of Giron-Nava et al. (2019a), which stated that northwest México is familiar with unsustainable fishing practices, since the open-access nature of fisheries leads to fleet overcapacity and intense competition for resources in the Gulf of California.

The high proportion of unsustainable stocks and the low values of the SDG 14.4.1 indicator calculated for reef and demersal fish in northwest México support our hypothesis, since it differs from broader assessments. For example, in the FAO report for the Eastern Central Pacific (which includes the Cortez and Californian provinces), 69.2% of stocks were classified as sustainable (FAO, 2025a). Differences between the present study and global fishery analyses could be attributed to the emphasis of global reports on industrial species of high commercial importance, including tunas, small pelagic fish, and shrimp (FAO, 2025a). In contrast, reef and demersal species (mostly caught through small-scale fishing) are often poorly documented and have been historically underrepresented in stock assessments (Pinto et al., 2018). This underrepresentation could be due to monitoring challenges in artisanal fisheries and to poor institutional capacity, given the extensive social participation in this activity and the large number of vessels operating along the Mexican coasts (Arreguín-Sánchez & Arcos-Huitrón, 2011).

Although both biogeographic provinces exhibit high biodiversity (Robertson & Cramer, 2009), the Cortez province showed a greater collection of fishery resources, probably because of the great variety of habitats and environmental conditions, that allows the confluence of temperate and tropical species (Robertson & Cramer, 2009; Arellano-Peralta & Medrano-González, 2013). The initial differences in the number of stocks analyzed at each biogeographic province strengthen the need to interpret this study as a preliminary regional assessment. Regarding the results obtained for each biogeographic province, the Cortez province showed a lower proportion of sustainable stocks under the Flexible and Conservative approaches than the Californian province. In contrast, under the Intermediate approach, both provinces presented 11% of sustainable stocks. These results (unsustainable stocks > 74% for the three approaches) were consistent with those reported by Giron-Nava et al. (2019a), who indicated that 69% of the stocks assessed in the Gulf of California were unsustainable. Conversely, the Californian province lacks reference values, making this regional study, to our knowledge, the first comprehensive assessment of the exploitation status of artisanal reef and demersal fisheries in this study area.

Based on the criteria of the Intermediate (FAO, 2025a) and Conservative (Joint Meeting of Tuna RFMOs, 2007) approaches, 11% of the stocks or less (9%) were classified as sustainable for both regions. In contrast, the Flexible approach (less conservative) showed that 26% of stocks in the Cortez province were sustainable (fully sustainable and not over-fished), whereas in the Californian province, this value reached 30%. Although a higher proportion of sustainable stocks in the Californian province could reflect more effective fisheries management, the results of this study are insufficient to confirm this, as fisheries management is only one of many factors influencing the population dynamics of finfish stocks (Lima et al., 2020).

The assessment of both biogeographic provinces exhibited that they are far from meeting the SDG 14 sustainable fisheries target (14.4; FAO, 2019). These results are consistent with regional reports for Central America, the Caribbean, and the Middle East, which have identified difficulties in meeting target 14.4 (Andriamahefazafy et al., 2022). However, it is necessary to consider that this target has the lowest percentage of compliance globally compared to other SDG 14 targets, mainly associated with the complexity of its evaluation due to the lack of information (*e.g.*, catch records) reported at the regional and local level (Andriamahefazafy et al., 2022).

While broader reports, such as those by FAO for fisheries around the world or IMIPAS for fisheries in México, tend to focus on stocks of great commercial importance that are generally classified as sustainable (INAPESCA, 2023; FAO, 2025a), this regional analysis showed that when only reef and demersal stocks are considered, the status is mostly unsustainable. Because of this, the results of this assessment could be integrated into the national fisheries evaluations, in order to identify overlooked sustainability issues and to provide a wider picture of the finfish resources status in the most fished region in México. A key factor underlying the unsustainability status observed in reef and demersal fish could be attributed to their biological traits, since slow-growing species exhibit relatively small stock size (B/B_MSY_ ≈ 0.49) compared to fast-growing, commercially important species like small pelagic fish (B/B_MSY_ ≈ 0.85; Costello et al., 2012). Small stock size represents a disadvantage for many fishery resources that have been historically captured but not consistently assessed until now (or for which no public results exist; Costello et al., 2012). Thus, preliminary regional analyses, such as this multi-specific assessment based on data-limited models, should be replicated for other regions in the country.

Related to the stock analysis, it has been documented that CMSY models provide more conservative estimates compared to other data-limited models, since their results tend to underestimate biomass and overestimate fishing mortality (Froese et al., 2018; Bouch, Minto & Reid, 2021; Sharma et al., 2021). However, CMSY models help assess overall views and provide snapshots of stock exploitation status to identify which fisheries and regions require further assessment (Rosenberg et al., 2014). A formal sensitivity analysis was beyond the scope of this study, thus the results of this assessment should be interpreted with caution, given the known sensitivity of CMSY models to parameter prior settings for r and B_end_/K (Chunyin et al., 2025). To complement stock assessments, other evaluations for establishing fishing bans, minimum fishing sizes, among other strategies, are necessary to be considered in northwest México to propose novel management plans in order to promote fisheries sustainability within the region (Apel, Fujita & Karr, 2013).

Regarding the exploitation approaches, the Intermediate approach considers a buffer of 20% of uncertainty around the estimate of B_MSY_ to make allowance for natural fluctuations in stock dynamics (*i.e.,* overexploitation occurs at B/B_MSY_ < 0.8, 20% below MSY), but it has been reported that this uncertainty does not adequately represent the magnitude of environmental variability (Hilborn, 2020b). Furthermore, assessments based on this approach often overestimate the number of overexploited stocks, even when biomass reductions are associated with natural fluctuations (Hilborn, 2020b).

In line with Hilborn et al. (2020a), this could indicate that both the Intermediate and the Conservative (where the overexploitation threshold is even stricter compared to the Intermediate approach) approaches underestimate the reductions in biomass generated by natural processes, thus making their estimates more theoretical and less representative of real-world conditions. This hinders the implementation of its results in fisheries management, especially under the current conditions of high food demand and a changing ocean (Garcia & Rosenberg, 2010; Stock et al., 2011), highlighting the need to reassess current methods and develop more applicable models.

Statistical tests confirmed differences between approaches, especially for the Flexible approach, which differed significantly from the Intermediate and Conservative approaches in both provinces. Since the choice of approach can influence the outcome, its selection should depend on the management objectives, as some fishery resources may require adopting more conservative strategies (Zhao et al., 2025; Feltrim & Dias, 2025). Additionally, the Flexible approach should be implemented with caution, since “not over-fished” stocks does not fully imply sustainability, but could contribute to identifying deteriorated stocks to propose expedited fisheries management (Fowler et al., 2021).

In the Cortez province, Giron-Nava et al. (2019a) reported the highest proportion of stocks as unsustainable (69%) while only 7% were sustainable. Similarly, in the present analysis a low proportion or absence of stocks in group 1 (sustainable stocks according to the three approaches) and 2 (sustainable stocks under the Flexible and Intermediate approaches), and a high representation of group 3 (unsustainable stocks for the Intermediate and Conservative approaches) and 4 (unsustainable stocks under the three approaches) was found. Within group 4, unsustainability was mainly driven by overexploitation (low B/B_MSY_ values; Giron-Nava et al., 2019b) due to severely depleted biomass. By contrast, Giron-Nava et al. (2019a) attributed unsustainability as a consequence of both overfishing (F/*F*_MSY_ > 1) and overexploitation. While overexploitation originates from overfishing, additional pressures such as pollution, habitat degradation, or climatic impacts can hinder stocks recovery, underscoring the need to consider both indicators (B/B_MSY_, F/*F*_MSY_) in fisheries assessments (Soeparna & Taofiqurohman, 2024).

Particular cases such as *C. princeps*, which was classified as unsustainable under the three approaches in the Californian province and had the smallest relative size (B/B_MSY_ = 0.184) among all the evaluated stocks, highlight the need for a precautionary interpretation of these results. For this stock, 250,000 tonnes were registered in 2014, representing a 20,549.0% increase compared to the previous year, when 1,210.9 tonnes were reported. This anomaly, reflected in the Kobe diagram (Fig. S3C), suggests that misidentification or reporting errors may influence stock assessments.

For other stocks (*Cynoscion spp*., *Epinephelus spp*., and *Paralabrax spp*.), the results indicated unsustainability for the Cortez province under the three approaches, which is similar to previous reports that indicated overexploitation within the same region (Arreguín-Sánchez & Arcos-Huitrón, 2011). This is relevant since it indicates that these stocks are still being exploited despite having been reported to be in poor condition since the last decade. Thus, joint action between fishers, academia, and decision-makers is needed to assess these particular cases and the management strategies to follow within the near future (Cisneros-Montemayor & Cisneros-Mata, 2018). In this sense, group 4 (unsustainable under the three approaches), which included the largest proportion of the stocks analyzed in both provinces, should be prioritized for urgent formal assessments to prevent further depletion and potential stock collapse.

In comparison with the latest edition of the CNP (INAPESCA, 2023), the results for certain stocks were similar. For example, according to the CNP, stocks such as *Mugil spp*. in the Cortez province were reported to be harvested at MSY. Consistently, this assessment also classified this stock as sustainable under the Flexible approach. Additionally, in the CNP, stocks like *P. nebulifer* were reported as being harvested at MSY in the Californian province, similar to the results of this evaluation (sustainable under the Flexible approach).

In contrast, other stocks (*Lutjanus spp*., *H. guentherii*, and *S. annulatus*) were classified as exploited at MSY in the CNP (INAPESCA, 2023), whereas in this assessment, they were found unsustainable in both provinces under all three approaches. These discrepancies could be associated with multiple factors, some of which are related to methodological issues in the fisheries data collection, as well as the unavailability of biological information and published records of previous exploitation assessments of these and other exploited fishery resources (Erisman et al., 2011; Erauskin-Extramiana et al., 2017). Despite the lack of detailed information, catch records constitute the primary source for the fisheries assessments in México, particularly for species that are not consistently evaluated (FAO, 1995; Purcell et al., 2010), thus, the present study could constitute a baseline for future evaluations of reef and demersal finfish.

Regarding resilience, 58% of the stocks assessed in both regions exhibited medium resilience levels (Froese & Pauly, 2025), indicating an intermediate capacity for most stocks to overcome disturbances (Musick, 1999). According to the fundamentals of the resilience classification, there could be a relationship between the resilience level and the exploitation status of stocks, where species with high resilience levels could support higher levels of exploitation compared to species with low resilience (Musick, 1999). However, the relationship between resilience level and exploitation status was not straightforward in this study. For instance, even a species with very low resilience (*e.g.*, *M. jordani*) showed a sustainable exploitation status in the Cortez Province according to both the Flexible and Intermediate approaches. On the contrary, stocks such as *Selene brevoortii* and *Selar crumenophthalmus*, with high levels of resilience, were classified as unsustainable for both provinces under the three approaches. Cases like these could indicate that even if a species has a high resilience level, this biological trait could not be sufficient to overcome overexploitation scenarios (Sarvala, Helminen & Ventelä, 2020).

Following the above, these findings suggest that the overall situation of the fisheries assessed in this study is far from optimal. Therefore, it is essential to strengthen evaluation efforts to establish a baseline for the status of reef and demersal finfish fisheries. For this, it is necessary to use reference points for multi-species fisheries, which, besides considering catch records (even in contexts of limited data), complement the assessments with local environmental variables (*e.g.*, coral cover, sea surface temperature, primary productivity, among others; McClanahan, Graham & Darling, 2014; Zamborain-Mason et al., 2023; Zamborain-Mason et al., 2025).

Additionally, future assessments should consider biological traits of the species, together with the socioeconomic, political, and ecological factors, in order to provide a regional context to the artisanal fishing activities (Cisneros-Montemayor & Cisneros-Mata, 2018; Savari, Sharifzadeh & Karami, 2024). Moreover, systematized information (*e.g.*, fishing effort, relative biomass, and long-term catch data identified at the species level) should be incorporated to enable more accurate evaluations of small-scale fisheries (Froese et al., 2023; Farouk-Abdelfattah, Schuchert & Farnsworth, 2024). In particular, multi-species stocks could be re-analyzed as more precise species-level catch data becomes available.

Finally, this study constitutes one of the first multi-specific assessment efforts in the Californian and Cortez provinces of reef and demersal finfish. These findings underscore the importance of evaluating underrepresented stocks and could contribute to further development of management and conservation strategies that benefit finfish and reef/demersal ecosystems, as well as the local communities that depend on these fisheries in northwest México.

## Conclusions

This assessment, based on the development of data-limited models, constitutes one of the first regional studies to determine compliance with the sustainable fishing goals and commitments in which México participates. It expands knowledge and availability of information about the exploitation status of reef and demersal finfish stocks in Mexican marine ecosystems. Through the analysis of 111 stocks in northwest México (2006–2023), it was determined that the majority of reef and demersal finfish stocks harvested in the Californian and Cortez provinces show signs of an unsustainable status, even under different exploitation approaches (Flexible, Intermediate, and Conservative). Thus, indicating that northwest México is far from meeting the sustainable fisheries target (14.4) of SDG 14. These results differ from what is reported nationally or globally, which might be associated with the lack of standardization in the evaluation of small-scale fisheries and the exclusion of some stocks from the official reports. Continuous monitoring of catch records, generation of biological information, homogenization in data collection, as well as the inclusion of as many stocks as possible in the analysis and periodic reports, are necessary for the sustainable management of Mexican fisheries, and for the national evaluation of Sustainable Development Goal 14 and other fisheries initiatives where México is engaged.

##  Supplemental Information

10.7717/peerj.21404/supp-1 Supplemental Information 1 Kobe diagrams based on the relationship between relative stock size and exploitation rate showing the exploitation status of analyzed stocks in the biogeographic provinces (2006–2023) Each point represents a year, and the color legend indicates the probability that the most recent year falls into one of the following states: unsustainable (red), overfishing (orange), overexploited (yellow), and sustainable (green). The ellipse represents uncertainty for the most recent year with confidence intervals of 50% in beige, 80% in gray, and 95% in dark gray.

## Additional Information and Declarations

### Data Availability

The following information was supplied regarding data availability: The data is available at GitHub and Zenodo - https://github.com/lecofym/Sustainability_Mexican_Finfish_Stocks
- lecofym. (2025). lecofym/Sustainability_Mexican_Finfish_Stocks: Assessing the sustainability of reef and demersal fish stocks in Northwest México under a data-limited approach (Sustainability_Mexican_Finfish_Stocks). Zenodo. https://doi.org/10.5281/zenodo.17945088.
