# Supplementary material for: Assessing the sustainability of reef and demersal fish stocks in Northwest México under a data-limited approach
Source: PeerJ. 2026 Jul 2;14:e21404. doi: 10.7717/peerj.21404 (PMC13333131; doi:10.7717/peerj.21404)
Supplement: Supplemental Information 1 — Each point represents a year, and the color legend indicates the probability that the most recent year falls into one of the following states: unsustainable (red), overfishing (orange), overexploited (yellow), and sustainable (green). The ellipse represents uncertainty for the most recent year with confidence intervals of 50% in beige, 80% in gray, and 95% in dark gray. [file peerj-14-21404-s001.docx]

# Assessing the sustainability of reef and demersal fish stocks in Northwest México under a data-limited approach

Valerie Juárez-Vera^1^, Georgina Ramírez-Ortiz^2^, Fernando Aranceta-Garza^3^, Felipe Amezcua^2^.

1. Departamento de Ciencias de la Tierra, Facultad de Ciencias, Universidad Nacional Autónoma de México, Alcaldía Coyoacán, Ciudad de México, México.

2. Instituto de Ciencias del Mar y Limnología, Unidad Mazatlán, Universidad Nacional Autónoma de México, Av. Joel Montes Camarena S/N Col. Playa Sur 82040, Mazatlán, Sin. México.

3. CONAHCYT-Centro de Investigaciones Biológicas Del Noroeste S.C, Av. Instituto Politécnico Nacional 195, Playa Palo de Santa Rita Sur, 23096 La Paz, B.C.S, México.

Corresponding author:

Georgina Ramírez-Ortiz^1^

Av. Joel Montes Camarena S/N Col. Playa Sur 82040, Mazatlán, Sin. México.

Email address: [gramirezortiz@ola.icmyl.unam.mx](mailto:gramirezortiz@ola.icmyl.unam.mx)

**Kobe diagrams for the Californian province**

| a) | 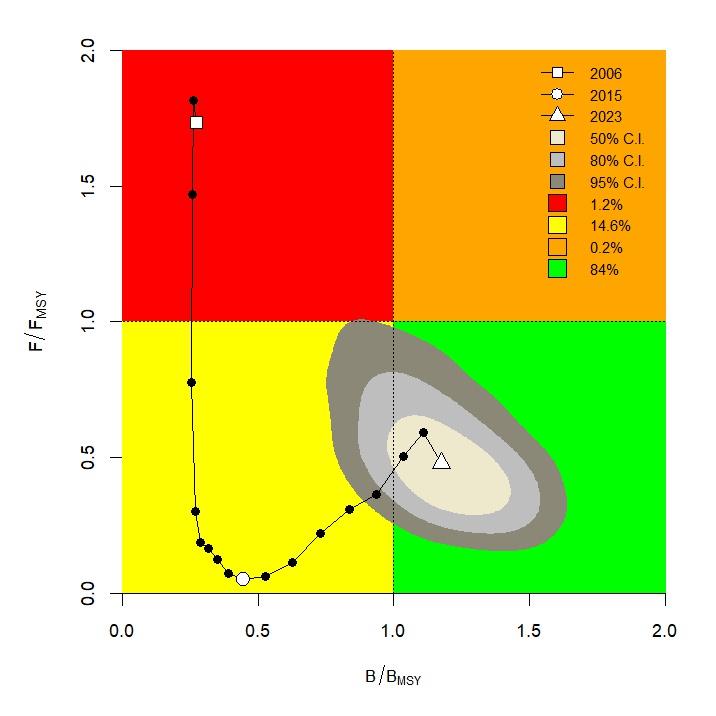 | b) | 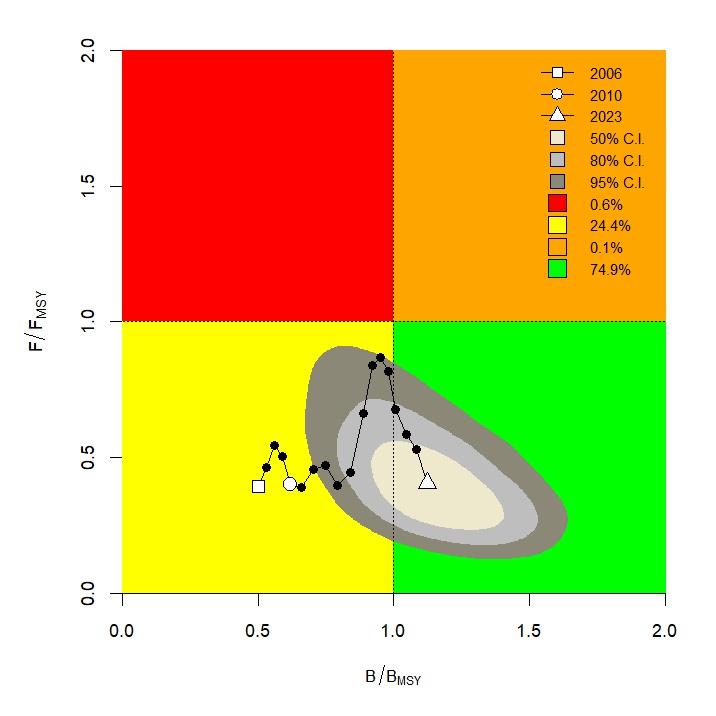 |
| --- | --- | --- | --- |
| c) | 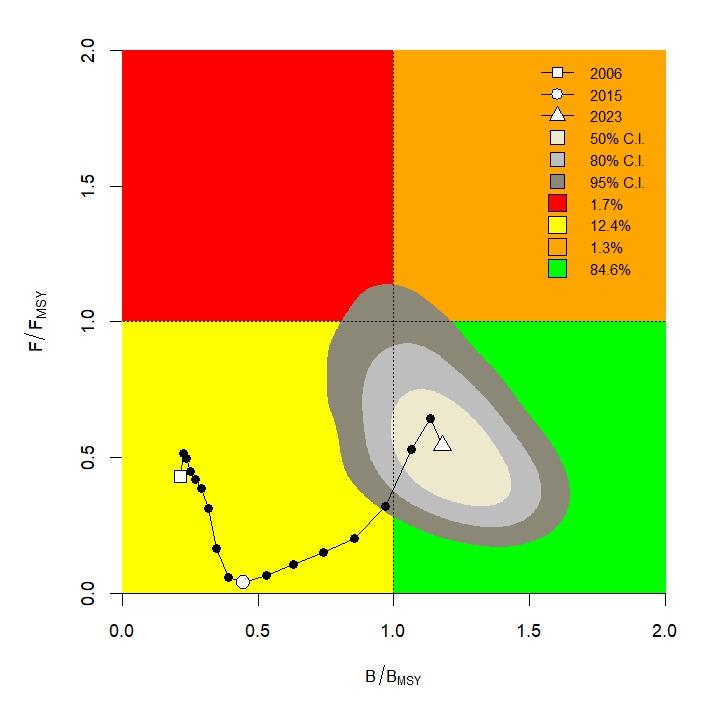 | d) | 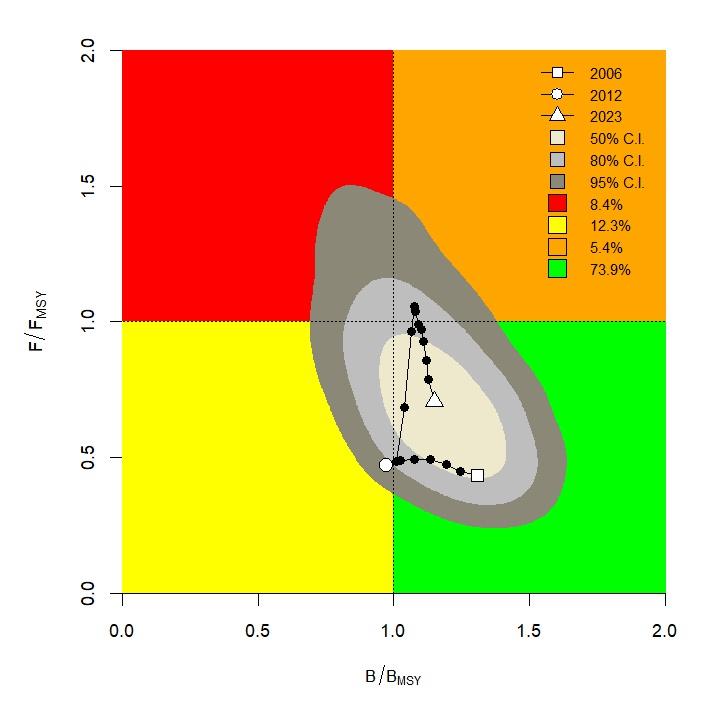 |
| e) | 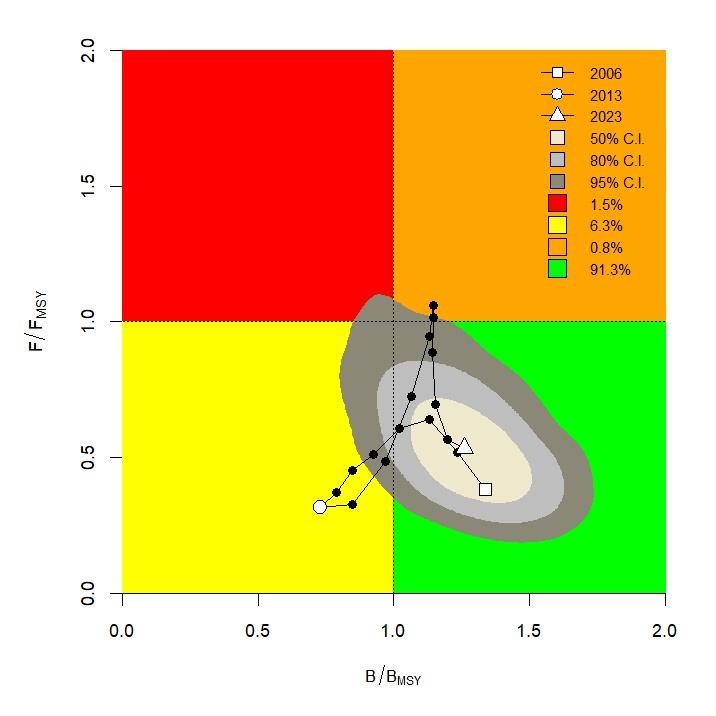 |  | **Figure S1**: **Kobe diagrams based on the relationship between biomass (B) and fishing pressure (F) relative to maximum sustainable yield (MSY), illustrating the status of the stocks in group 1 (sustainable under all the approaches) in the Californian province from 2006 to 2023**. a) *Brotula spp*., b) *Caulolatilus affinis*, c) *Diapterus spp*., d) *Mycteroperca rosacea*, and e) *Trachinotus paitensis*. Each point represents a year, and the color legend indicates the probability that the most recent year falls into one of the following states: unsustainable (red), overfishing (orange), overexploited (yellow), and sustainable (green). The ellipse represents uncertainty for the most recent year with confidence intervals of 50% in beige, 80% in gray, and 95% in dark gray. |

| a) | 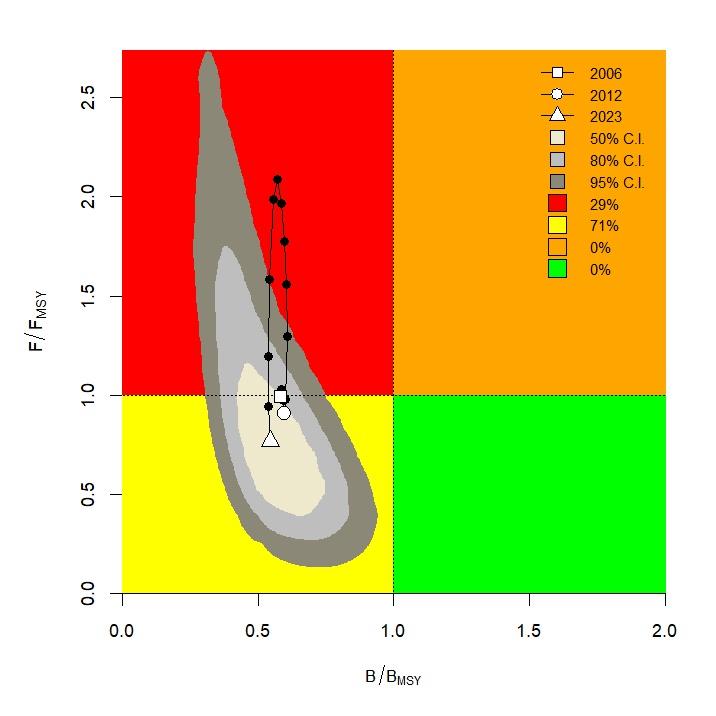 | b) | 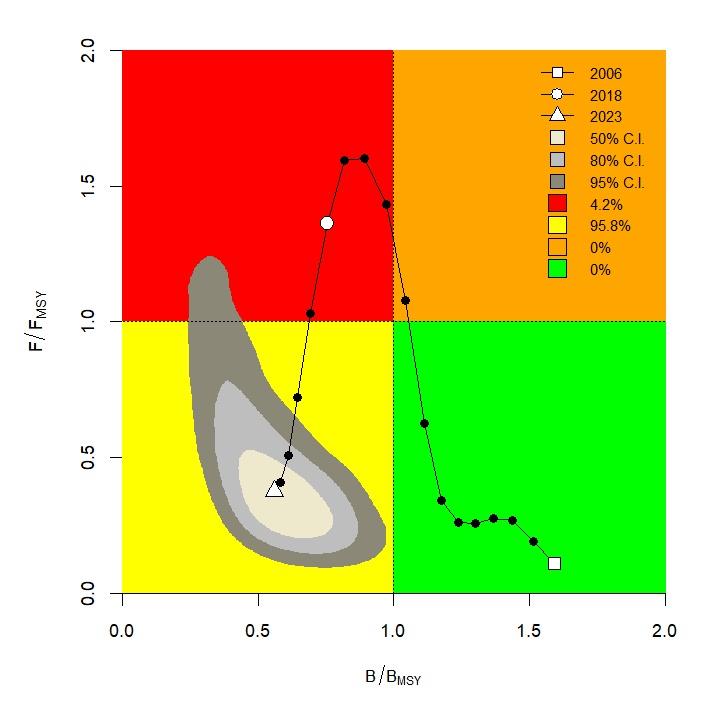 |
| --- | --- | --- | --- |
| c) | 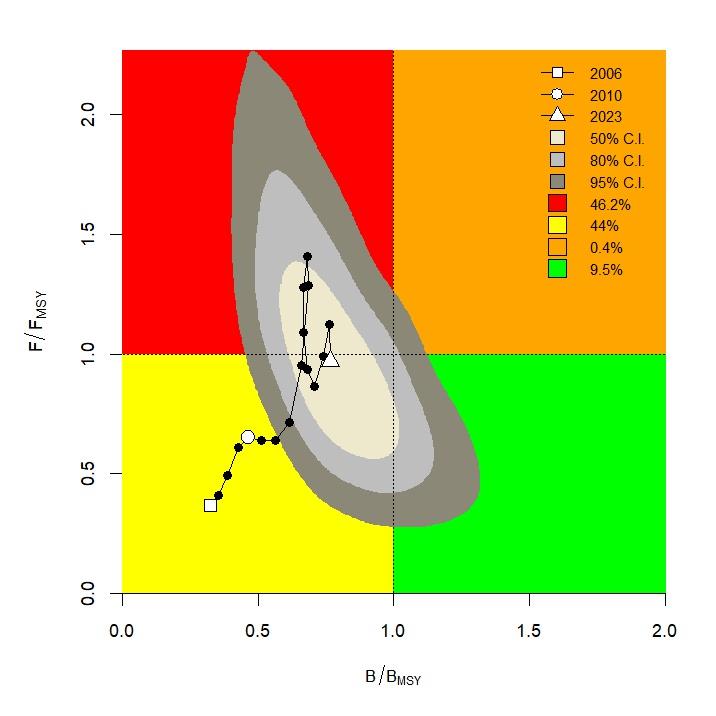 | d) | 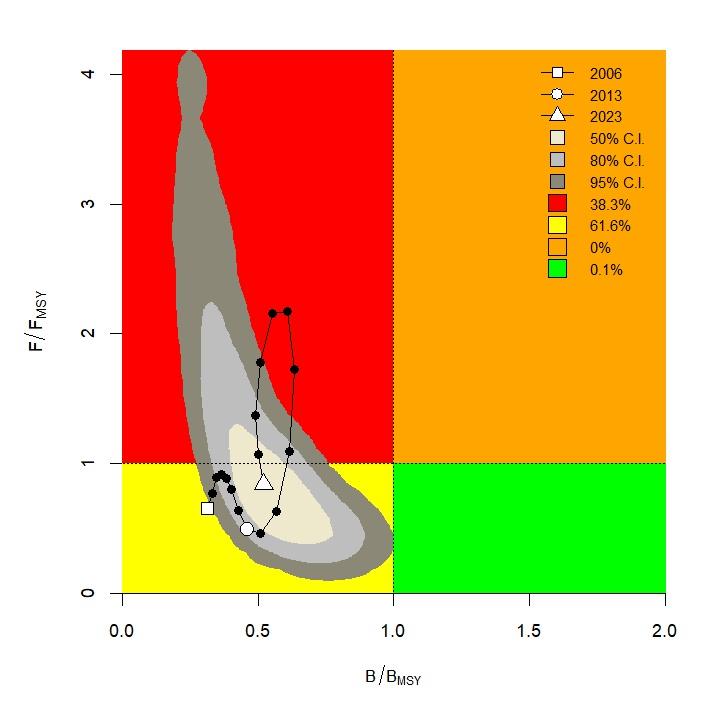 |
| e) | 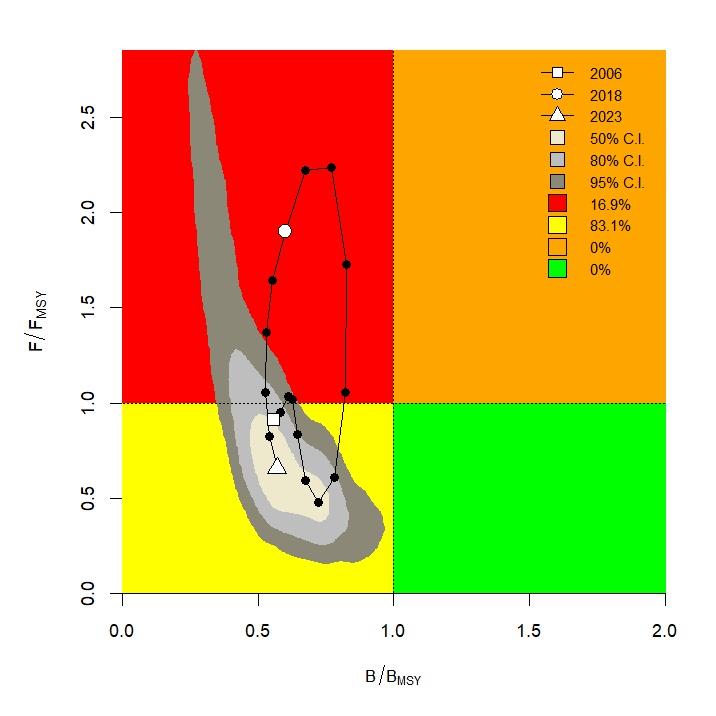 | f) | 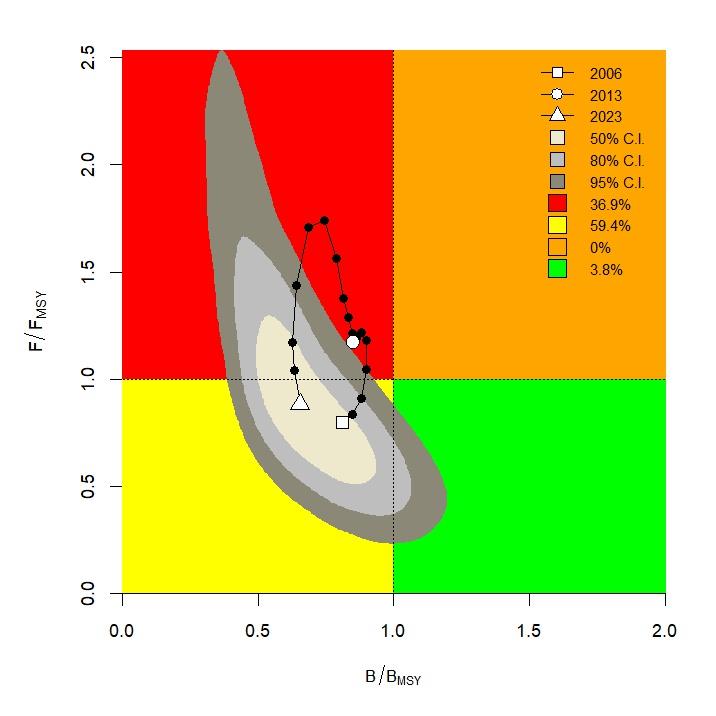 |
| g) | 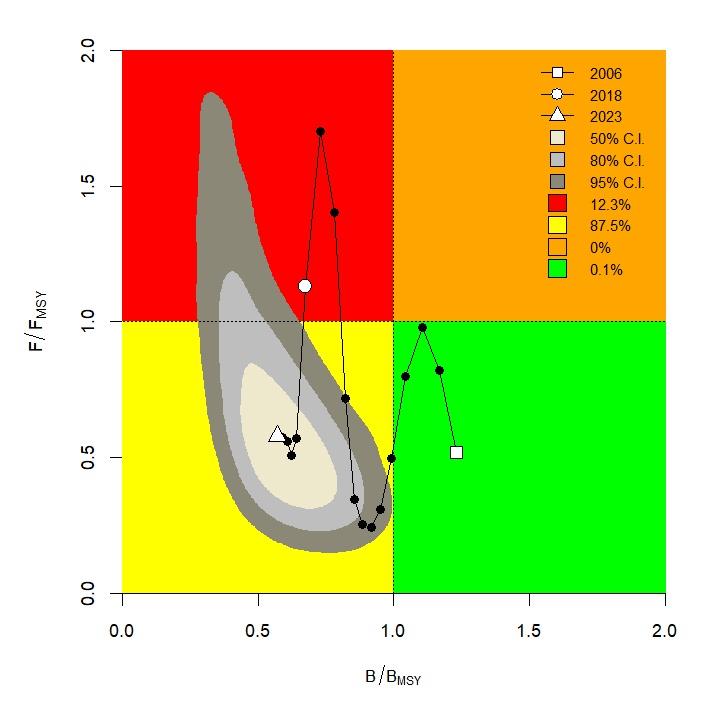 | h) | 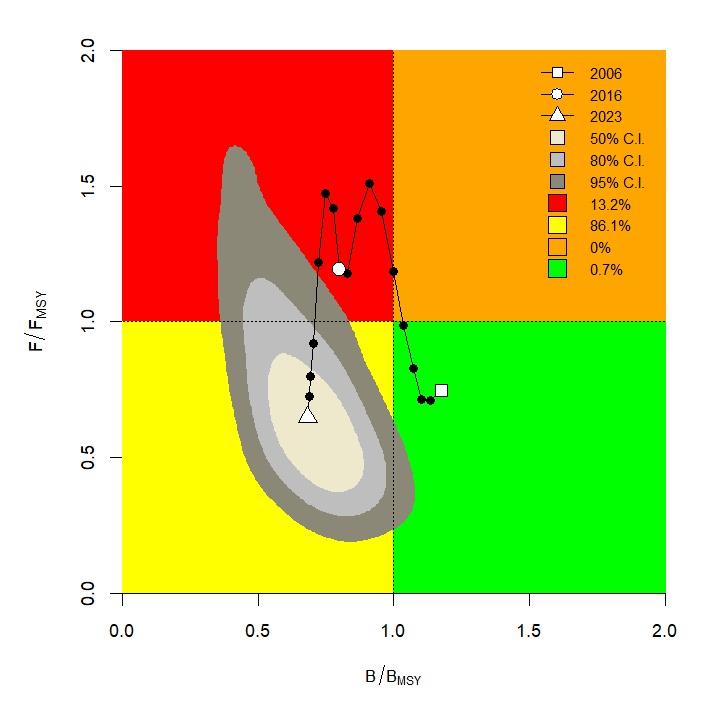 |
| i) | 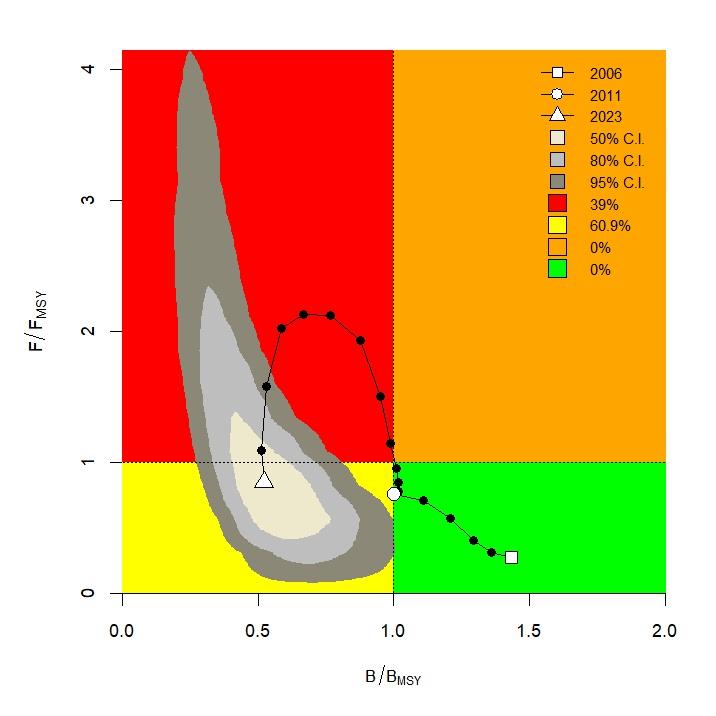 |  | **Figure S2: Kobe diagrams based on the relationship between biomass (B) and fishing pressure (F) relative to maximum sustainable yield (MSY), illustrating the status of the stocks in group 3 (not over-fished under the Flexible approach) in the Californian province from 2006 to 2023**. a) *Anisotremus interruptus*, b) *Balistes polylepis*, c) *Centropomus spp*. CAL, d) *Hyporthodus niphobles*, e) *Menticirrhus elongatus*, f) *Paralabrax nebulifer*, g) *Paralabrax spp*., h) *Scorpaena mystes*, and i) *Trachinotus spp.* CAL. Each point represents a year, and the color legend indicates the probability that the most recent year falls into one of the following states: unsustainable (red), overfishing (orange), overexploited (yellow), and sustainable (green). The ellipse represents uncertainty for the most recent year with confidence intervals of 50% in beige, 80% in gray, and 95% in dark gray. |

| a) | 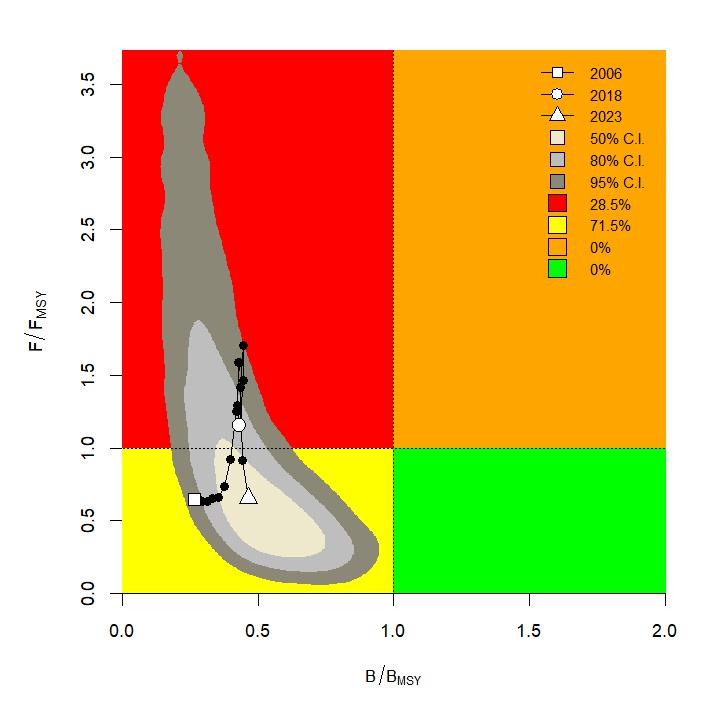 | b) | 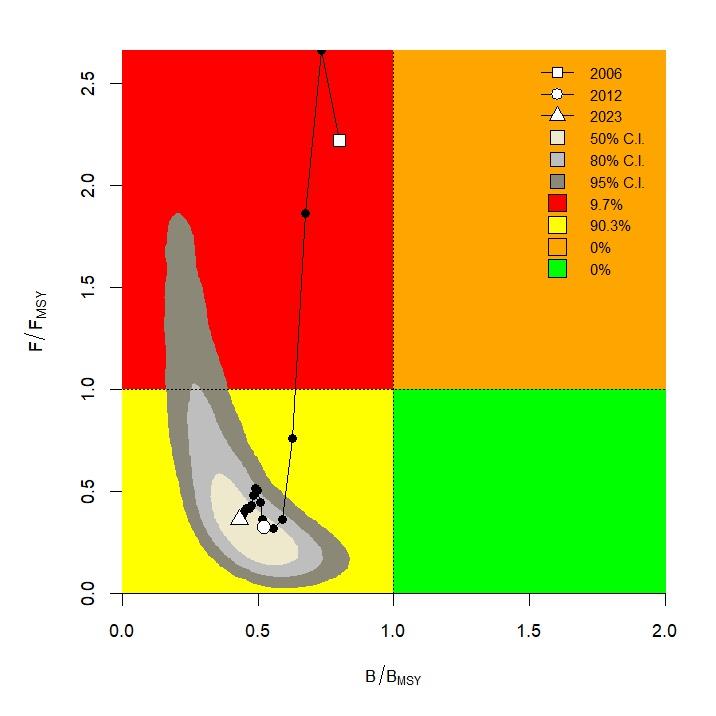 |
| --- | --- | --- | --- |
| c) | 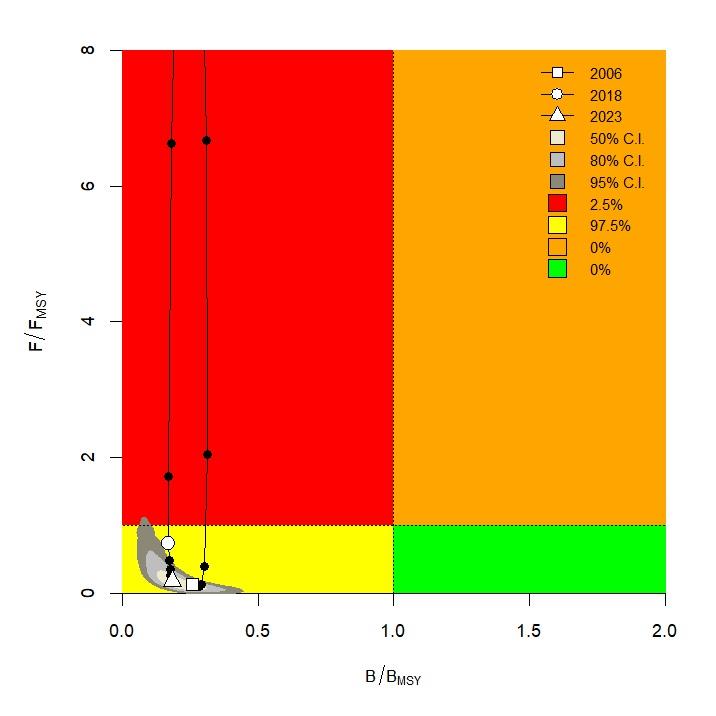 | d) | 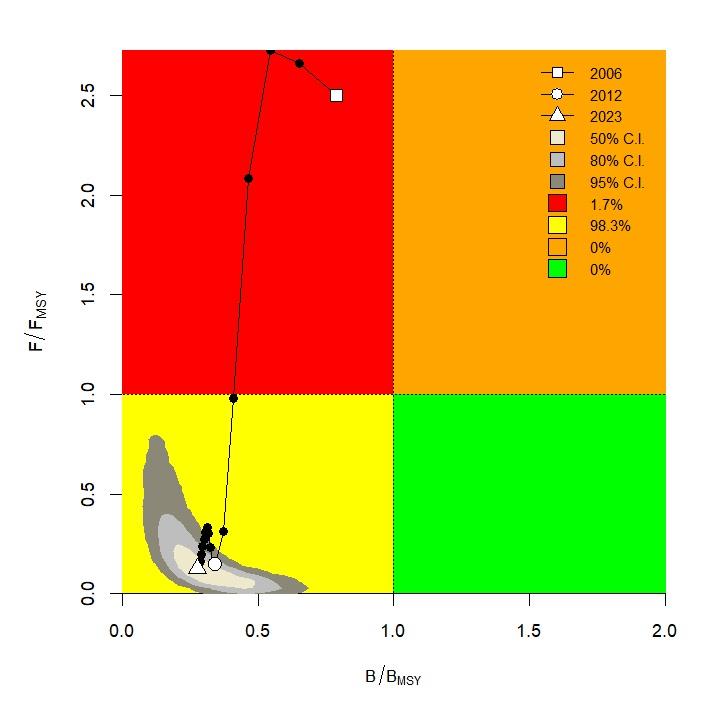 |
| e) | 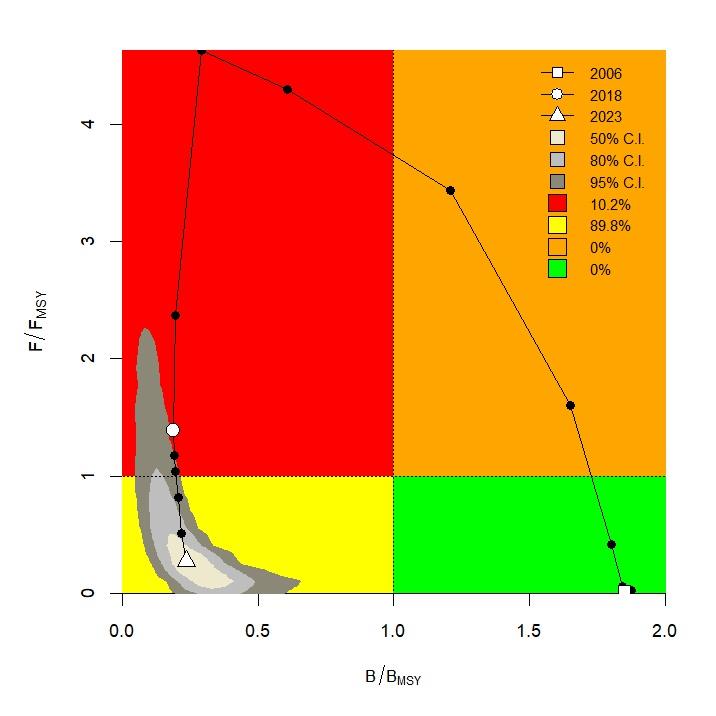 | f) | 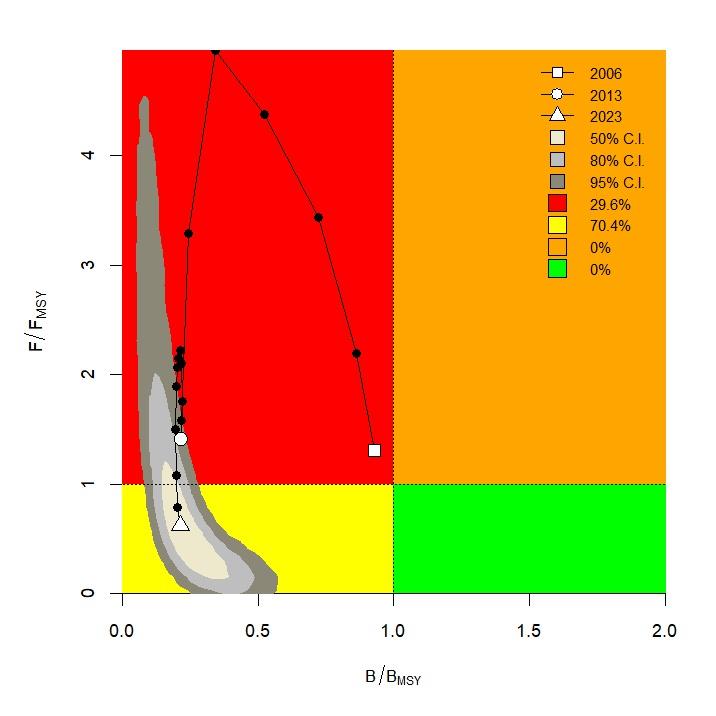 |
| g) | 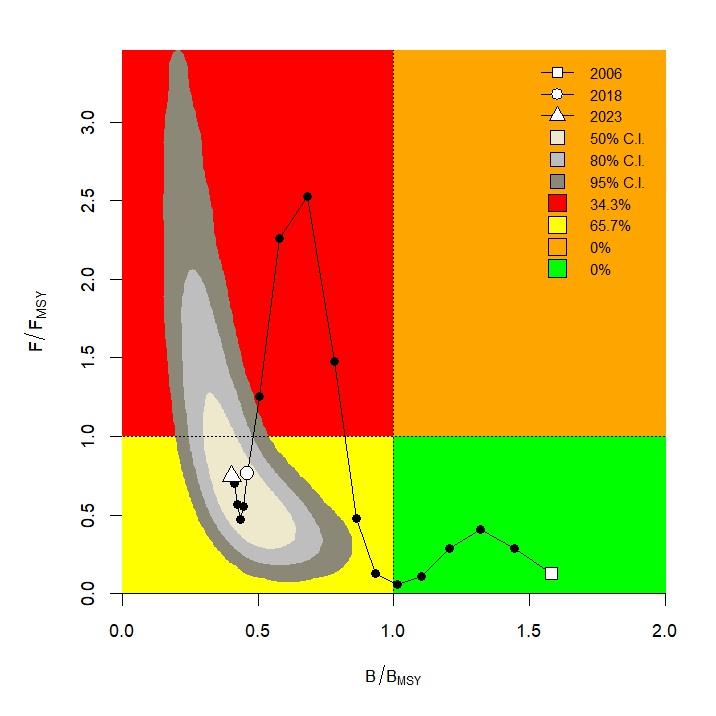 | h) | 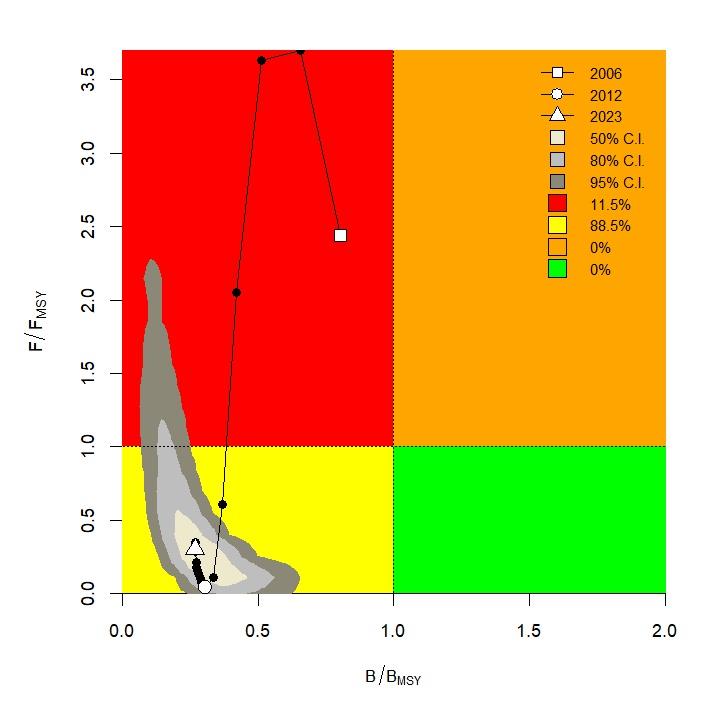 |
| i) | 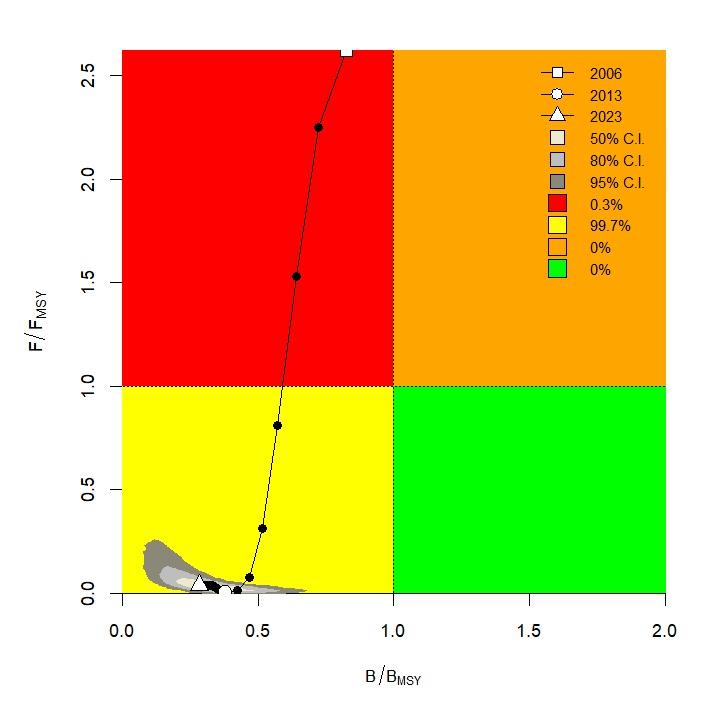 | j) | 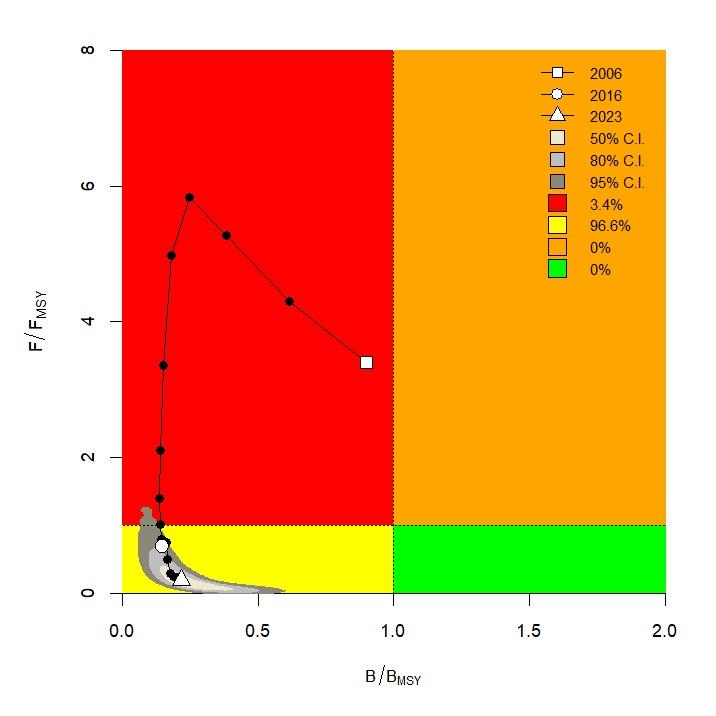 |
| k) | 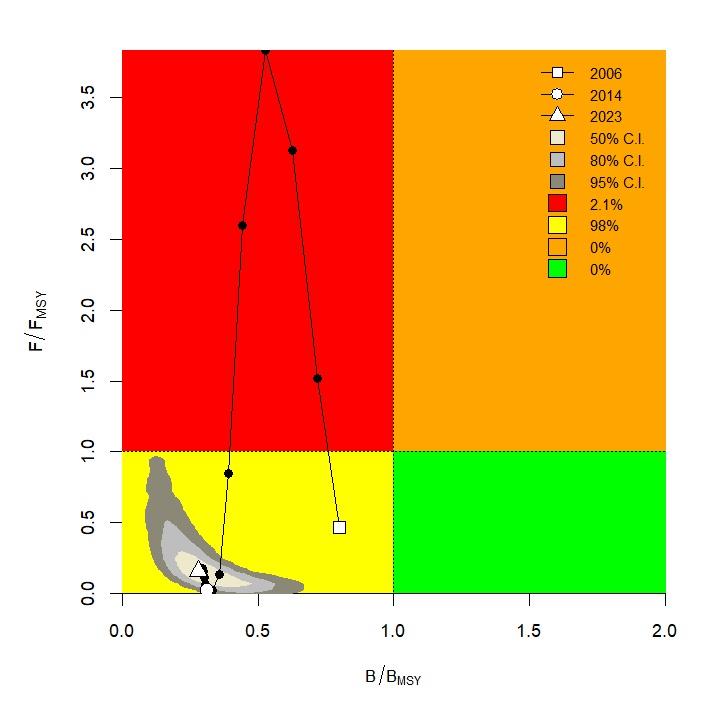 | l) | 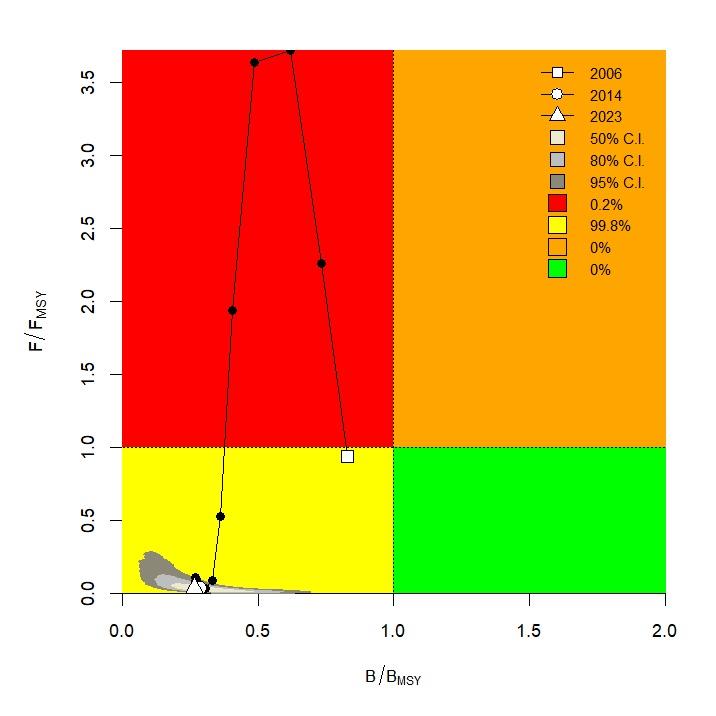 |
| m) | 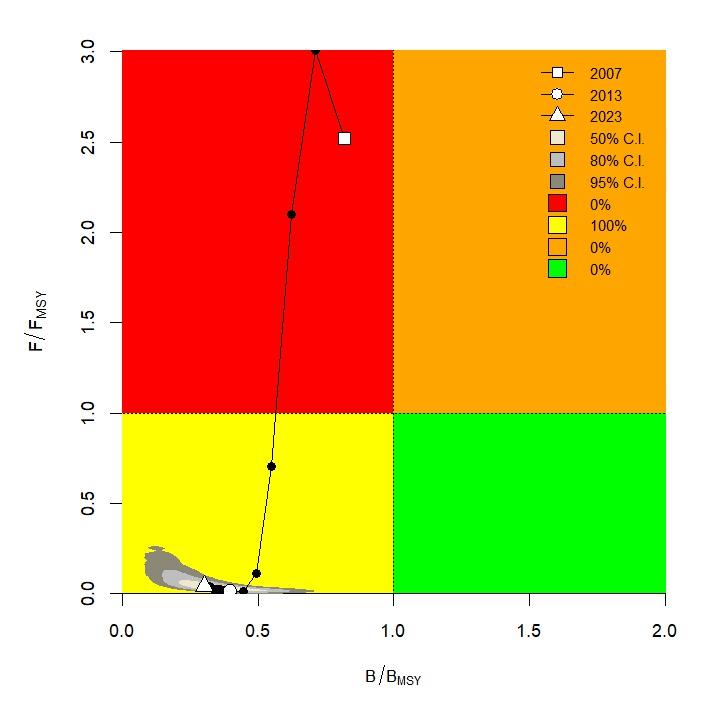 | n) | 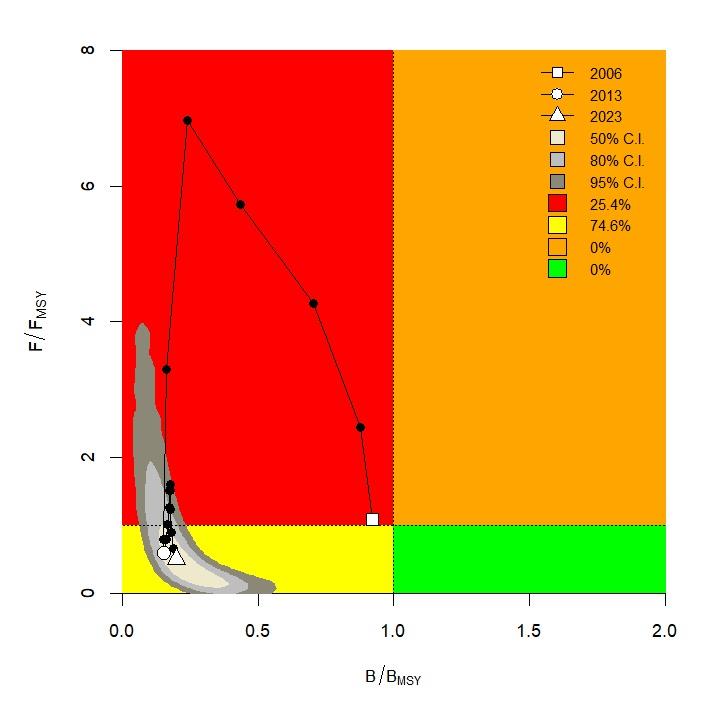 |
| o) | 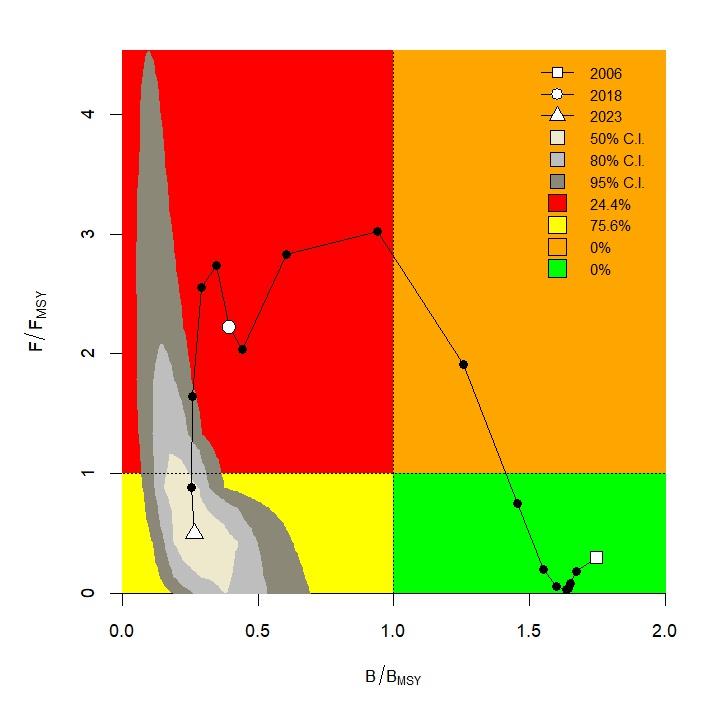 | p) | 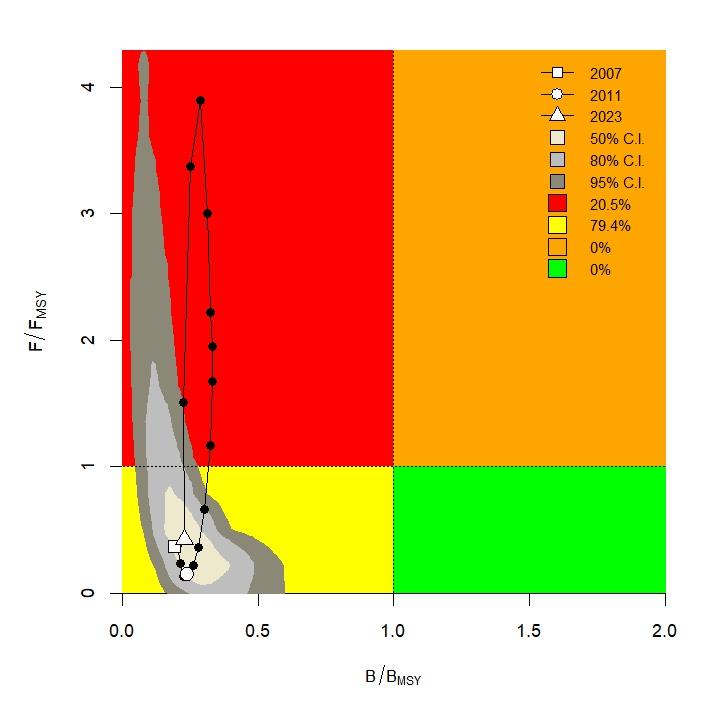 |
| q) | 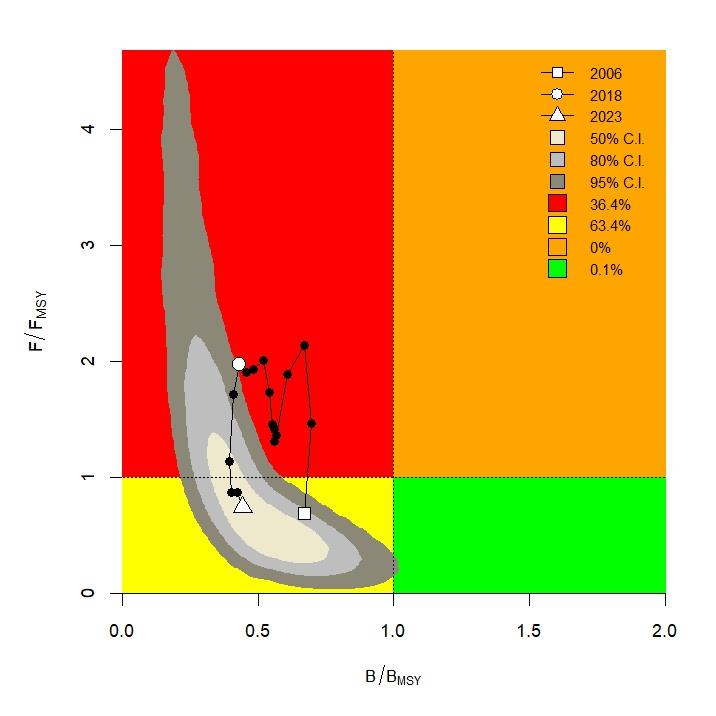 | r) | 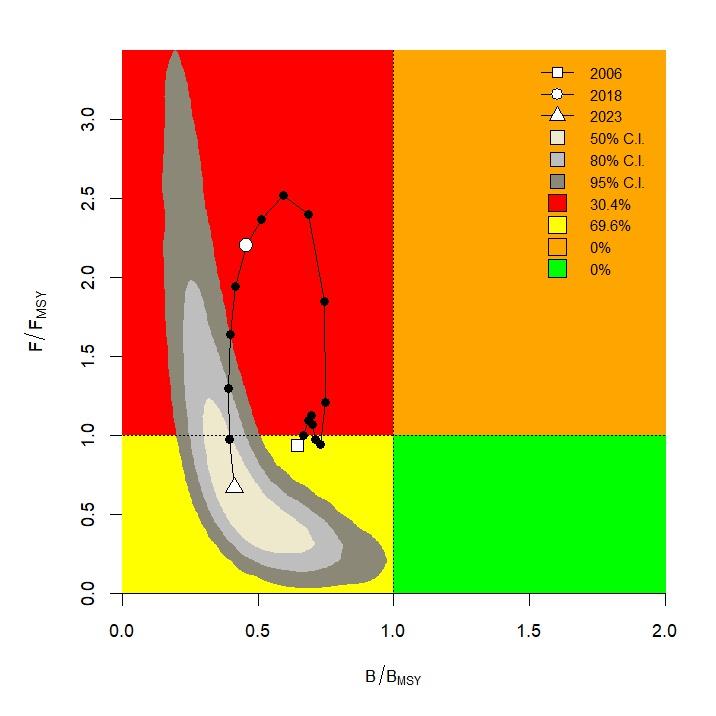 |
| s) | 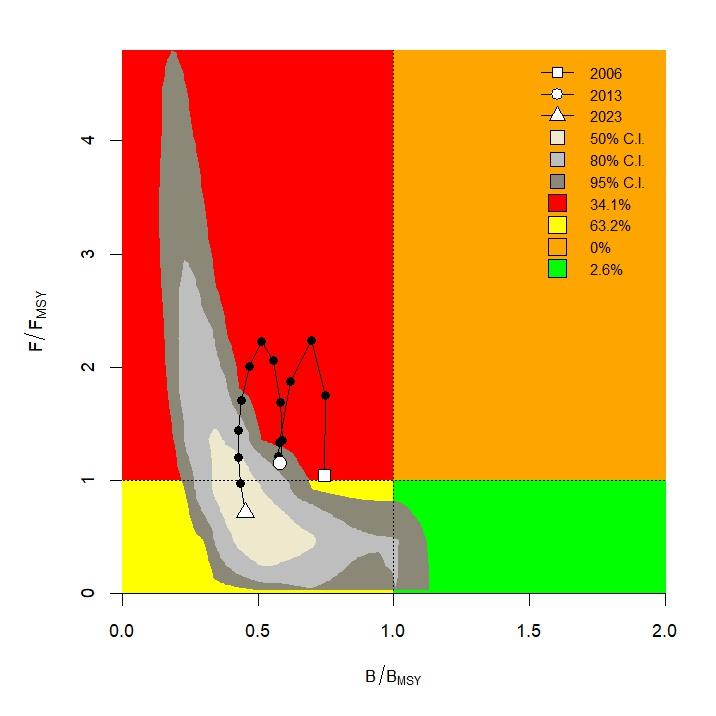 | t) | 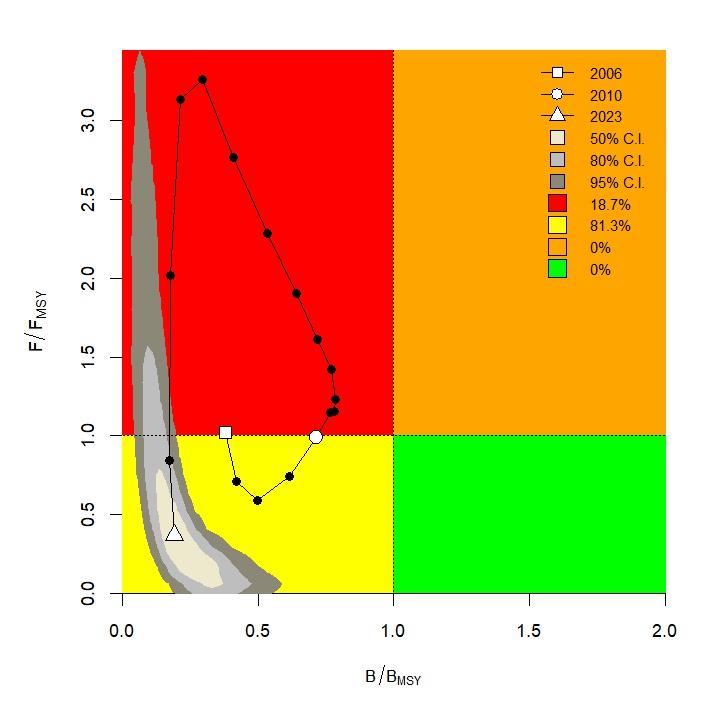 |
| u) | 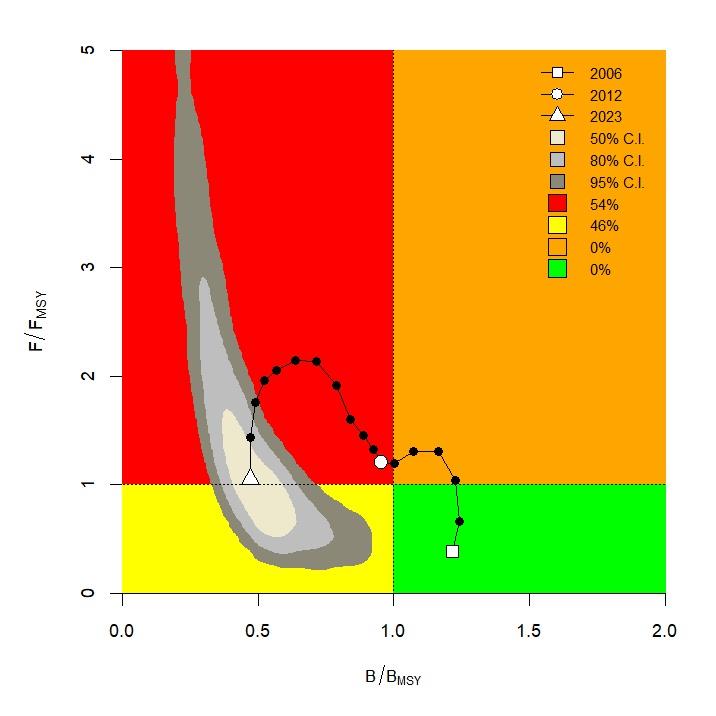 | v) | 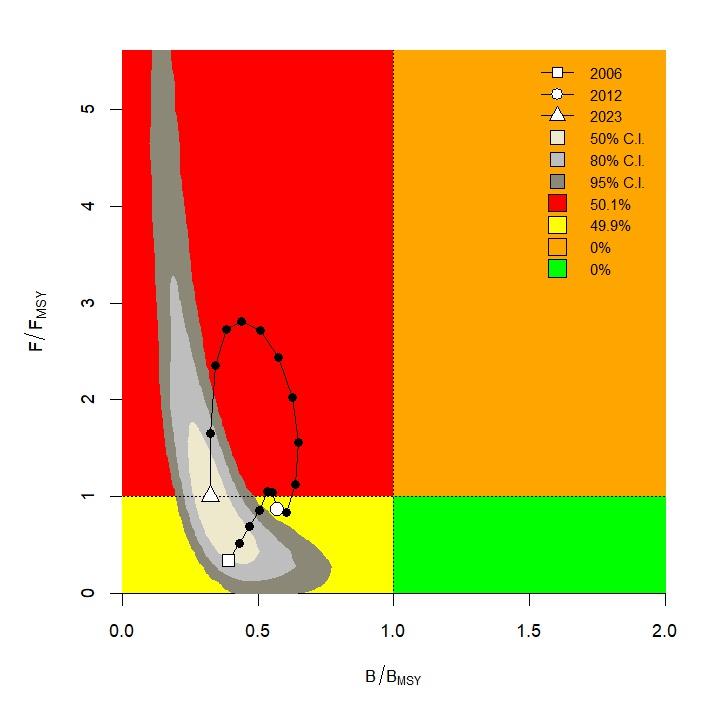 |
| w) | 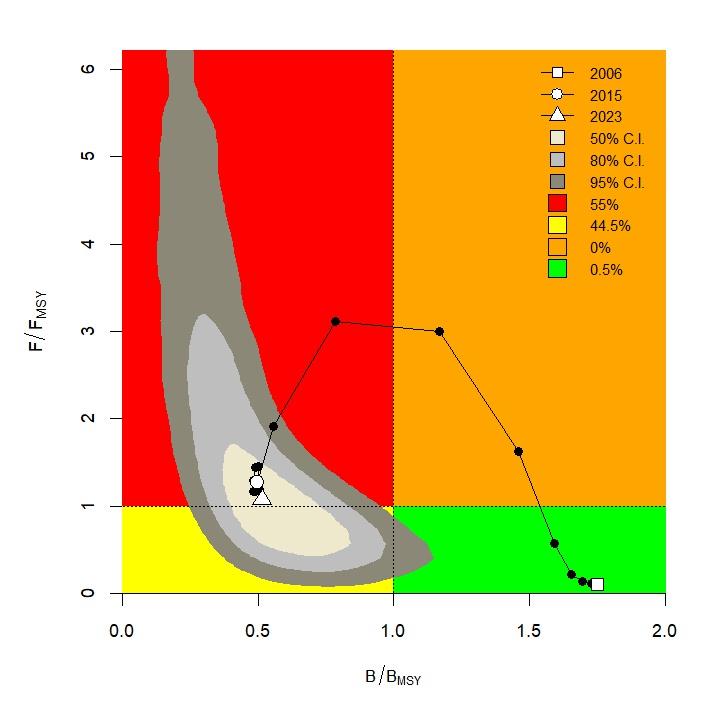 | x) | 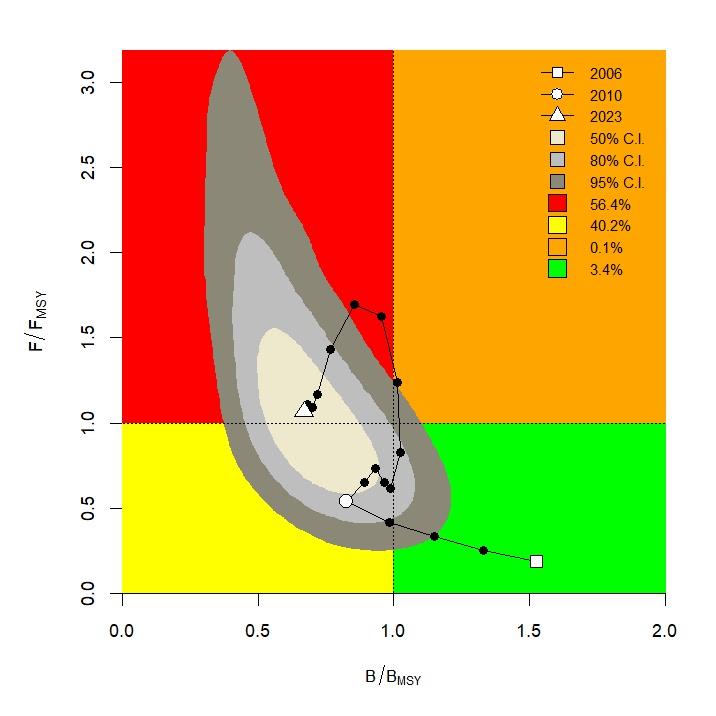 |
| y) | 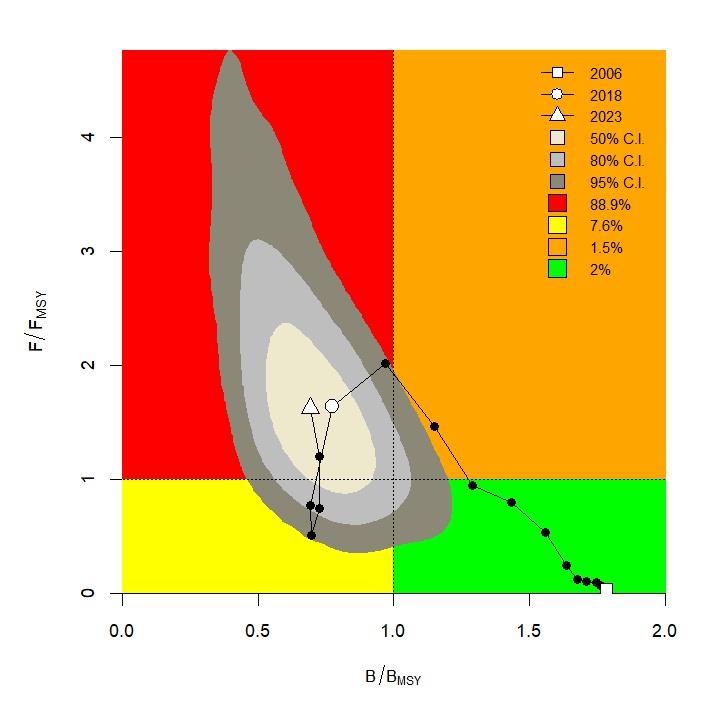 | z) | 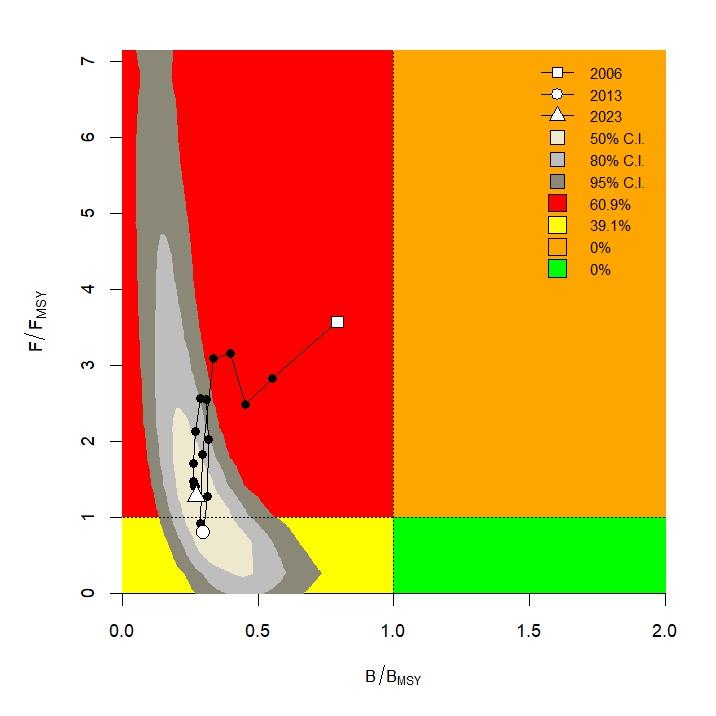 |
| aa) | 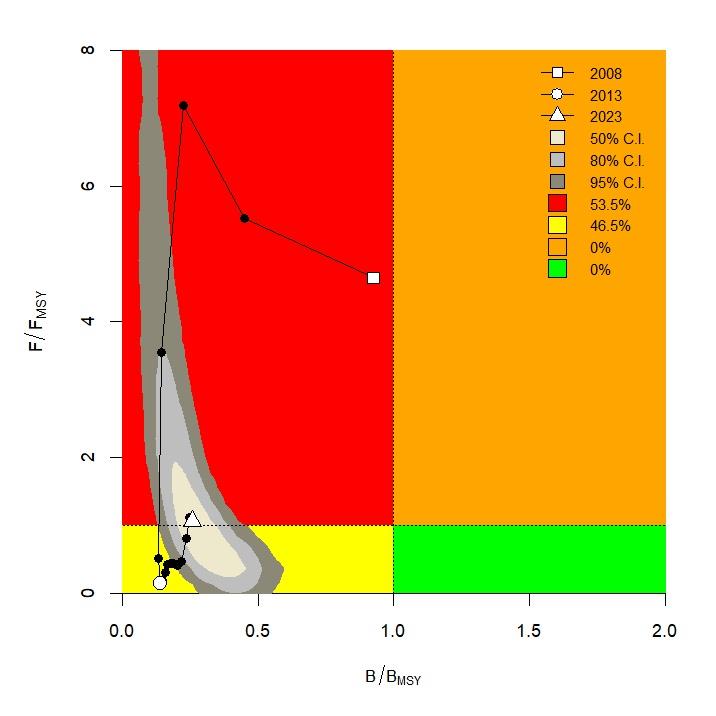 | ab) | 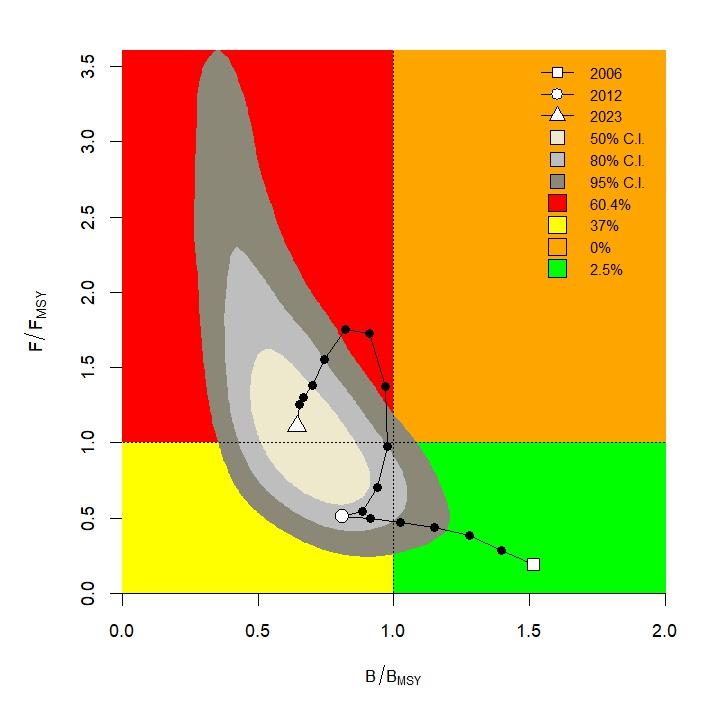 |
| ac) | 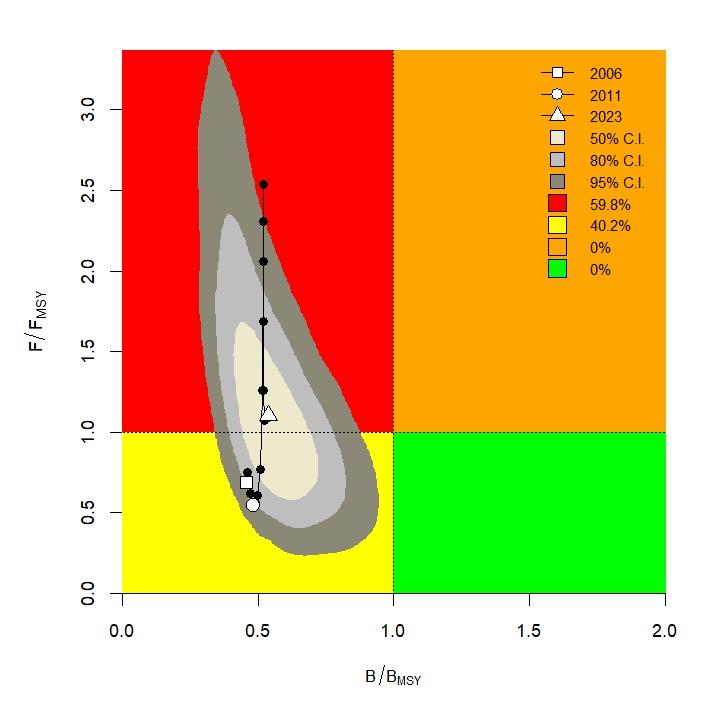 | ad) | 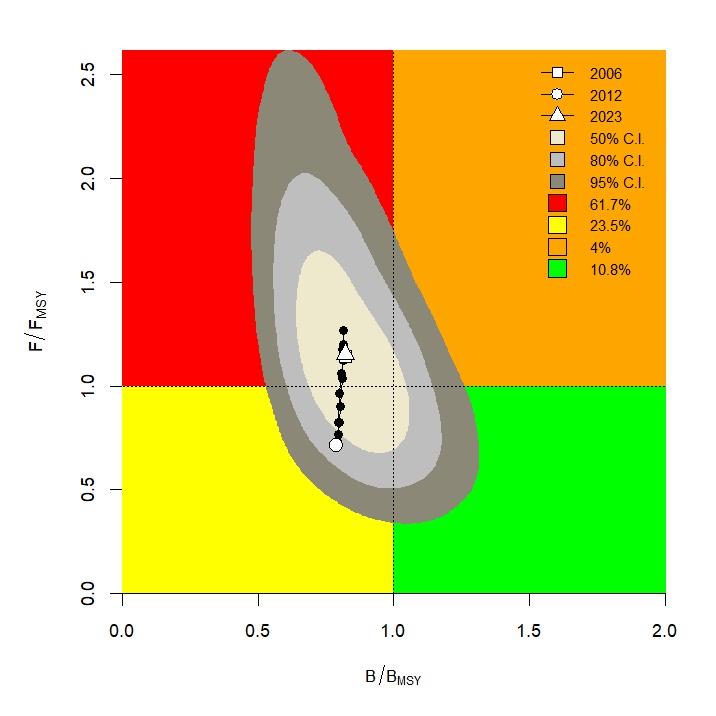 |
| ae) | 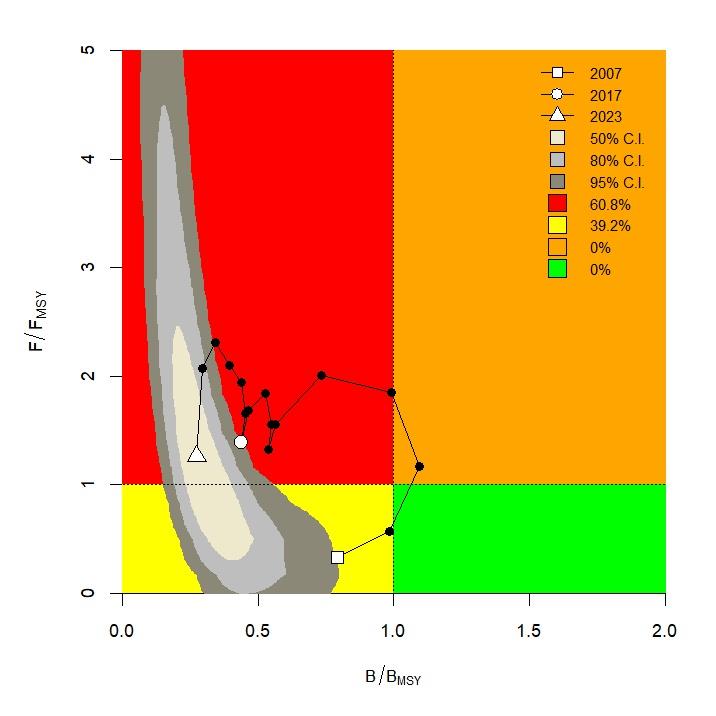 | af) | 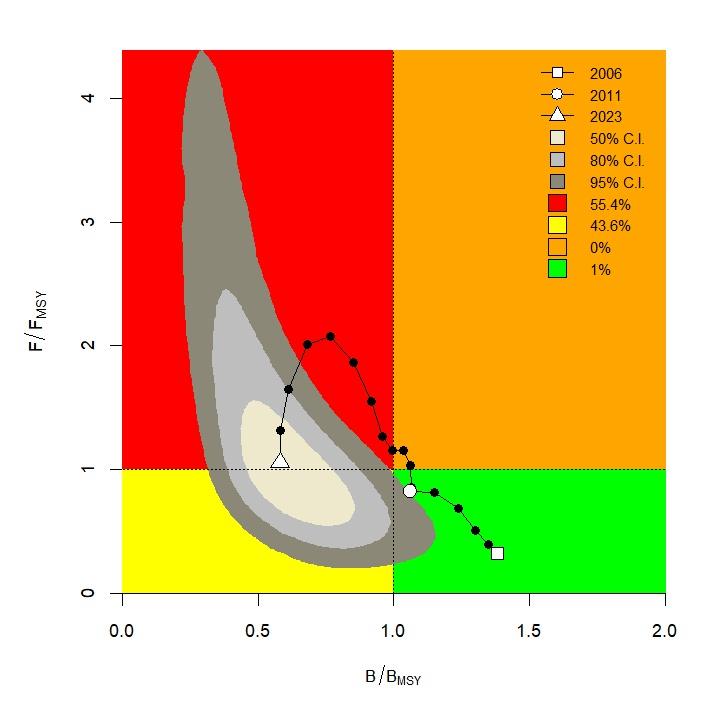 |

**Figure S3: Kobe diagrams based on the relationship between biomass (B) and fishing pressure (F) relative to maximum sustainable yield (MSY), illustrating the status of the stocks in group 4 (unsustainable under all the approaches) in the Californian province from 2006 to 2023**. a) *Bagre panamensis*, b) *Bodianus spp*., c) *Caulolatilus princeps*, d) *Cynocsion spp*. CAL, e) *Diplectrum euryplectrum*, f) *Eucinostomus spp*., g) *Haemulon spp*., h) *Holopragus guentherii*, i) *Hyporthodus acanthistius*, j) *Kyphosus spp*., k) *Lutjanus argentiventris*, l) *Lutjanus colorado*, m) *Lutjanus novemfasciatus*, n) *Lutjanus spp*., o) *Menticirrhus spp*., p) *Microlepidotus inornatus*, q) *Micropogonias megalops*, r) *Mugil spp*. CAL, s) *Scarus spp*. CAL, t) *Selar crumenophtalmus*, u) *Calamus spp*., v) *Cephalopholis colonus*, w) Cyclopsetta spp., x) *Diplectrum pacificum*, y) *Epinephelus analogus*, z) *Epinephelus spp*. CAL, aa) *Lutjanus guttatus*, ab*) Lutjanus peru*, ac) *Mycteroperca prionura*, ad) *Sebastes spp*., ae) *Selene brevoortii,* and af) *Sphoeroides annulatus*. Each point represents a year, and the color legend indicates the probability that the most recent year falls into one of the following states: unsustainable (red), overfishing (orange), overexploited (yellow), and sustainable (green). The ellipse represents uncertainty for the most recent year with confidence intervals of 50% in beige, 80% in gray, and 95% in dark gray.

**Kobe diagrams for the Cortez province**

| a) | 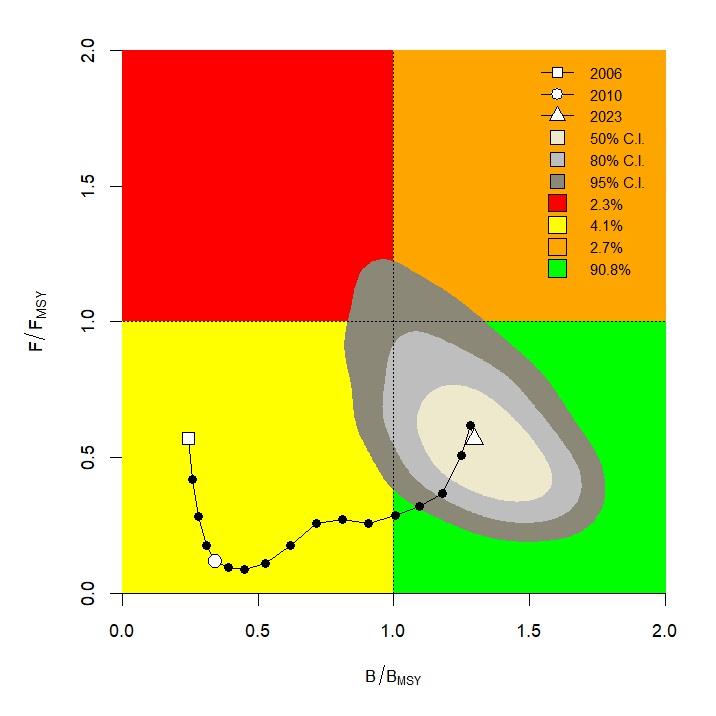 | b) | 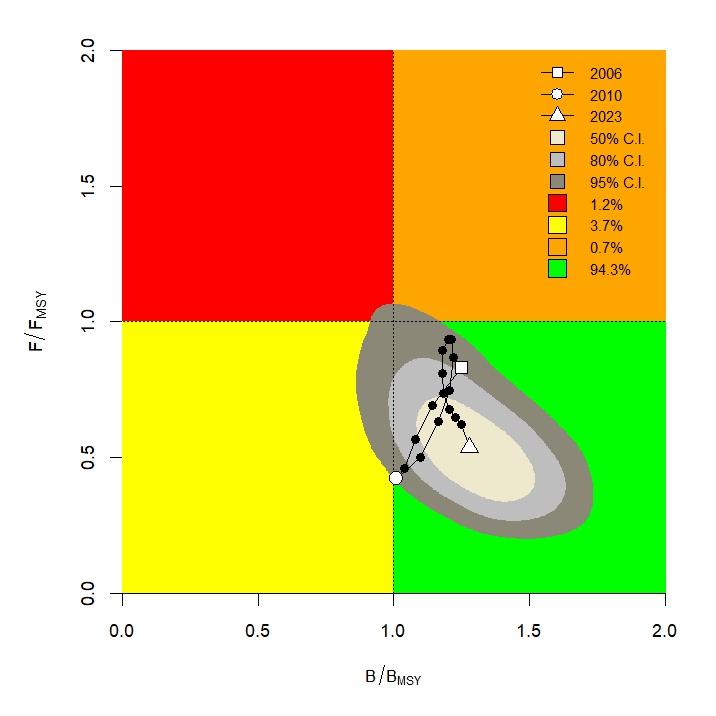 |
| --- | --- | --- | --- |
| c) | 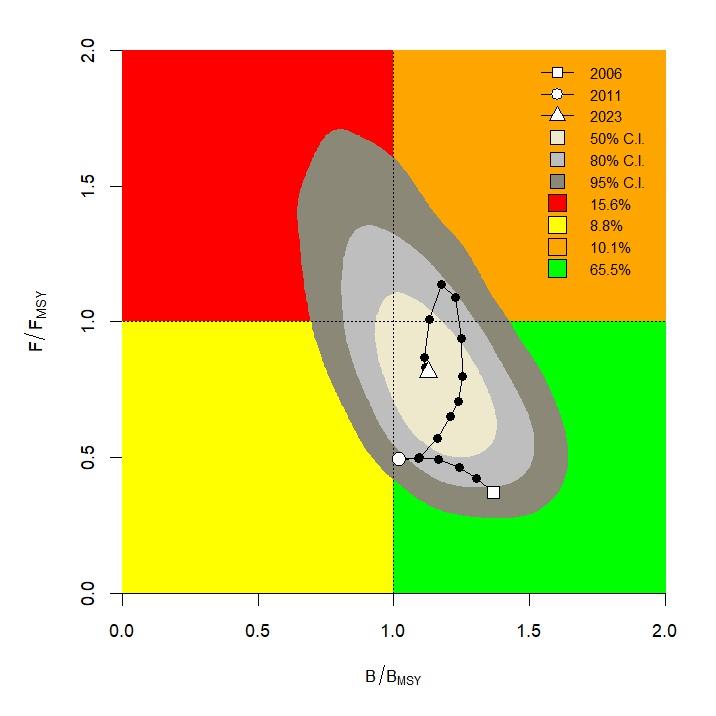 | d) | 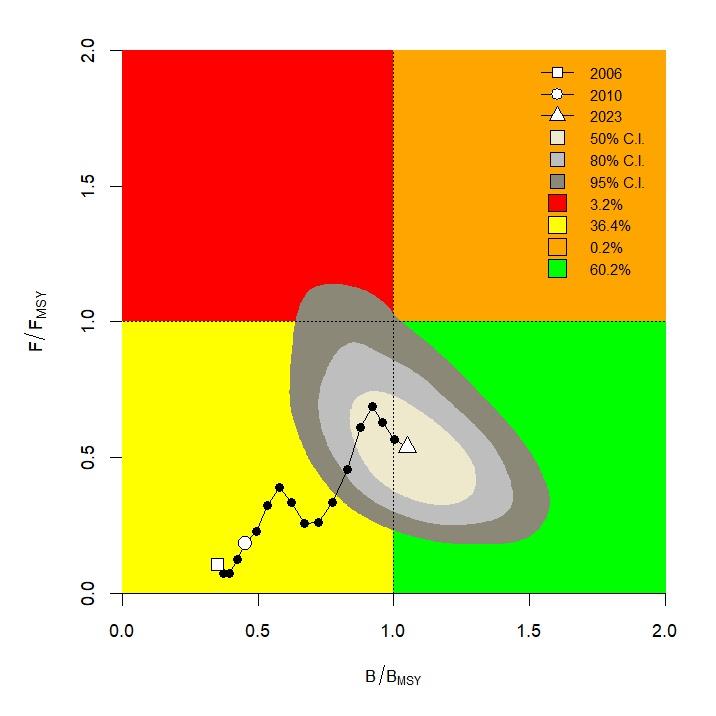 |
| e) | 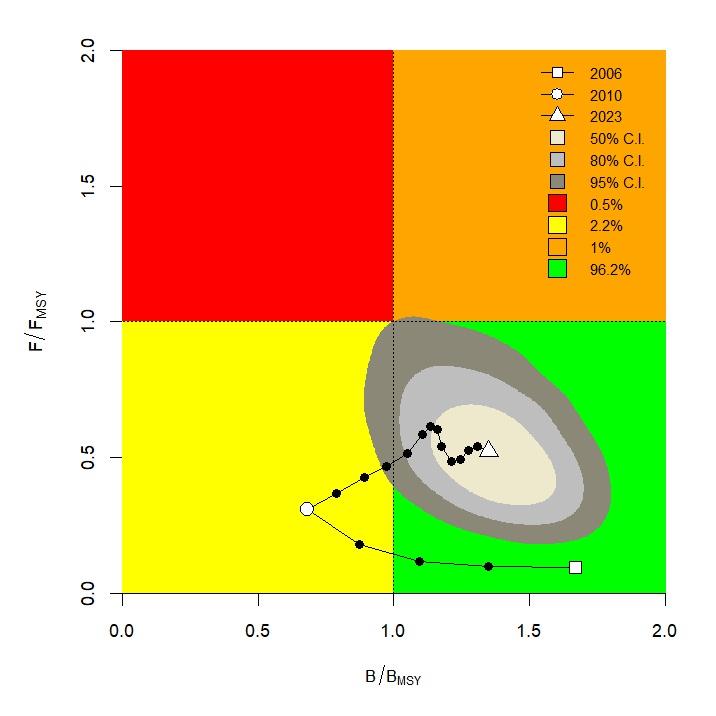 | f) | 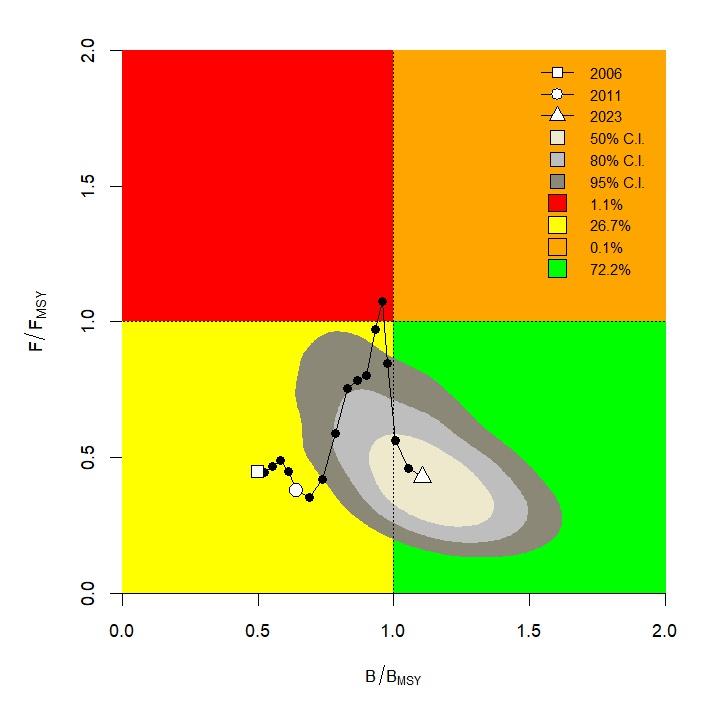 |
|  | **Figure S4**: **Kobe diagrams based on the relationship between biomass (B) and fishing pressure (F) relative to maximum sustainable yield (MSY), illustrating the status of the stocks in group 1 (sustainable under all the approaches) in the Cortez province from 2006 to 2023**. a) *Aluterus scriptus*, b) *Brotula spp*., c) *Centropomus spp*. COR, d) *Cynoscion xanthulus*, e) *Diplectrum pacificum*, and f) *Mycteroperca rosacea*. Each point represents a year, and the color legend indicates the probability that the most recent year falls into one of the following states: unsustainable (red), overfishing (orange), overexploitation (yellow), and sustainable (green). The ellipse represents uncertainty for the most recent year with confidence intervals of 50% in beige, 80% in gray, and 95% in dark gray. | | |


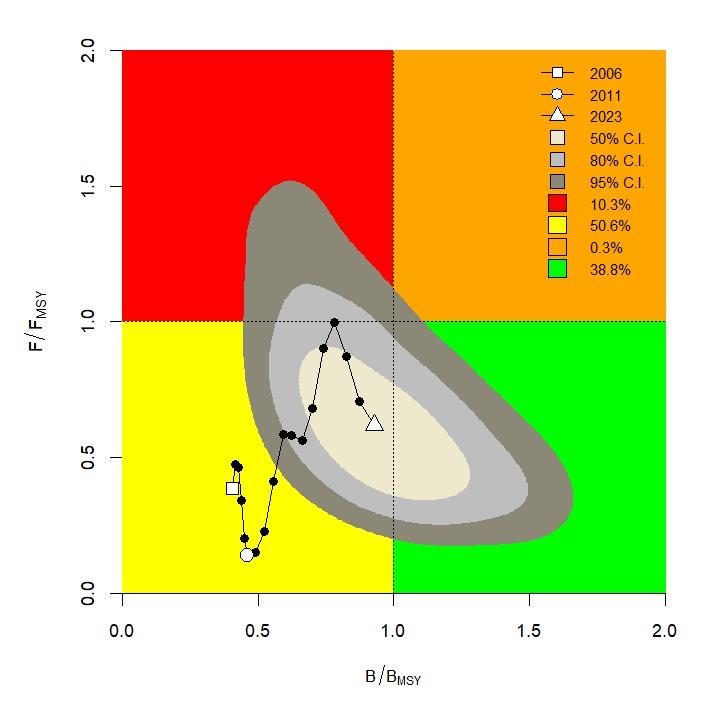


**Figure S5**: **Kobe diagram based on the relationship between biomass (B) and fishing pressure (F) relative to maximum sustainable yield (MSY), illustrating the status of group 2 stock *Mycteroperca jordani* (sustainable under the Flexible and Intermediate approaches) in the Cortez province from 2006 to 2023**. Each point represents a year, and the color legend indicates the probability that the most recent year falls into one of the following states: unsustainable (red), overfishing (orange), overexploitation (yellow), and sustainable (green). The ellipse represents uncertainty in the most recent year with confidence intervals of 50% in beige, 80% in gray, and 95% in dark gray.

| a) | 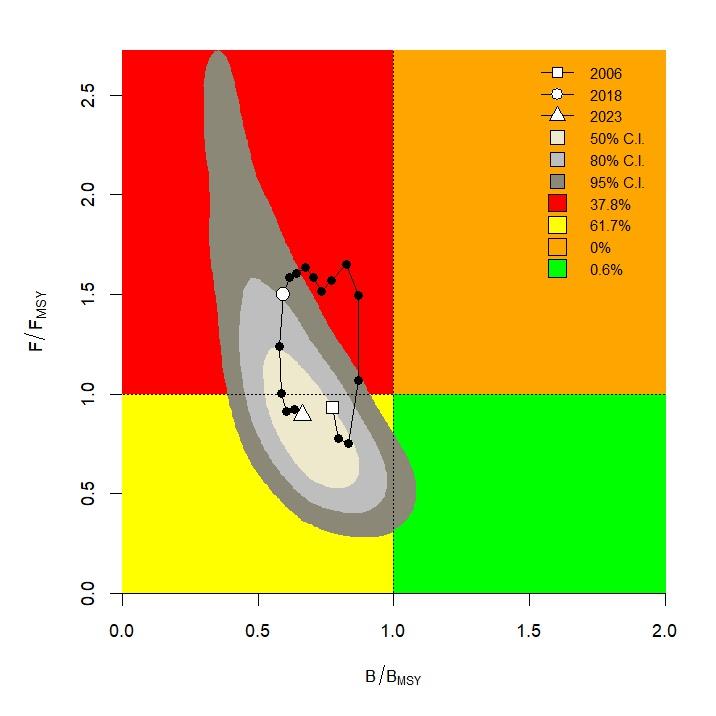 | b) | 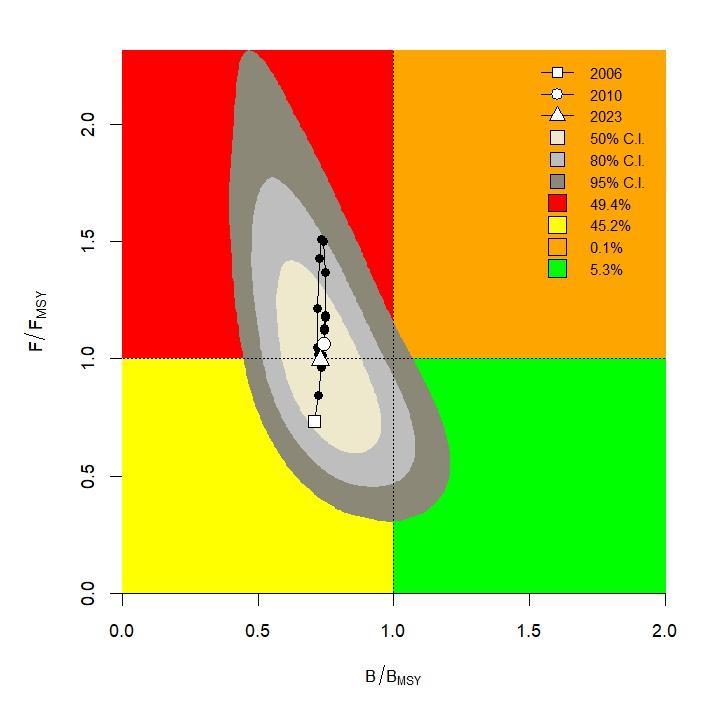 |
| --- | --- | --- | --- |
| c) | 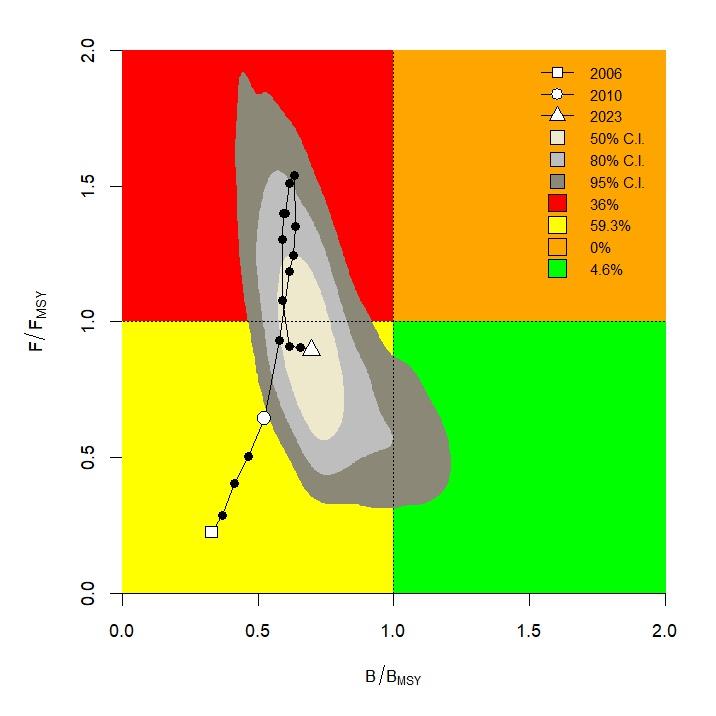 | d) | 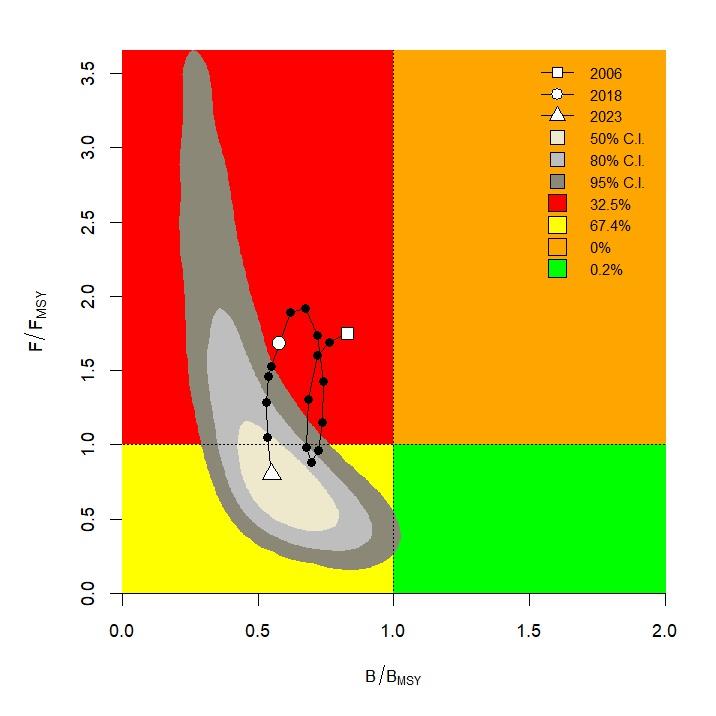 |
| e) | 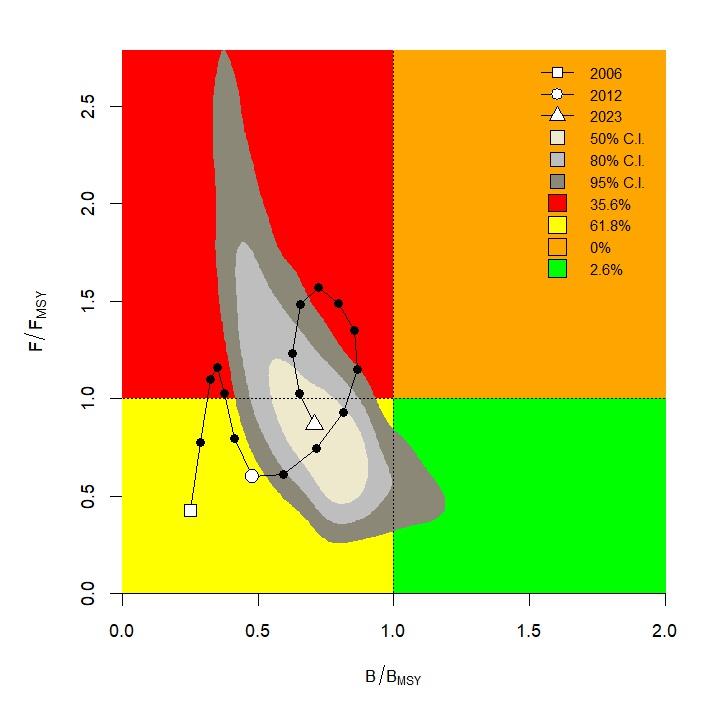 | f) | 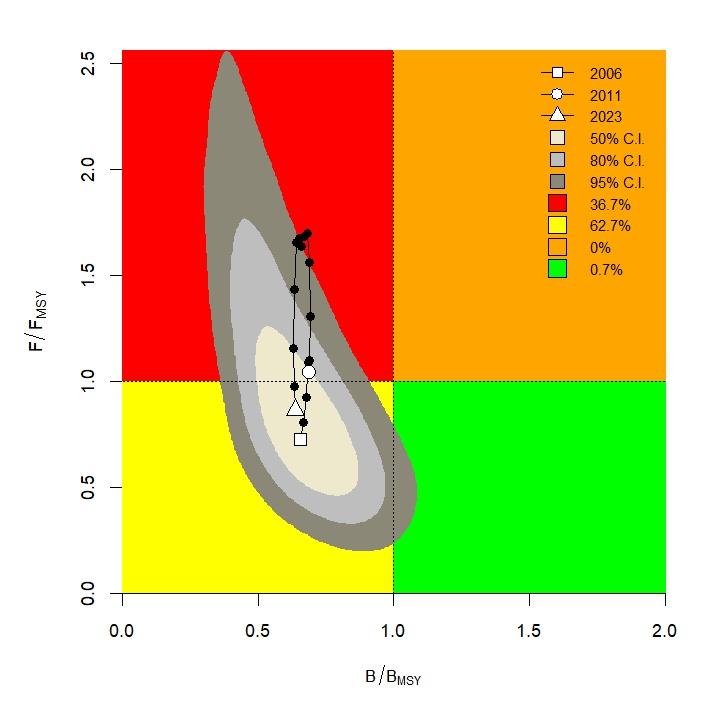 |
| g) | 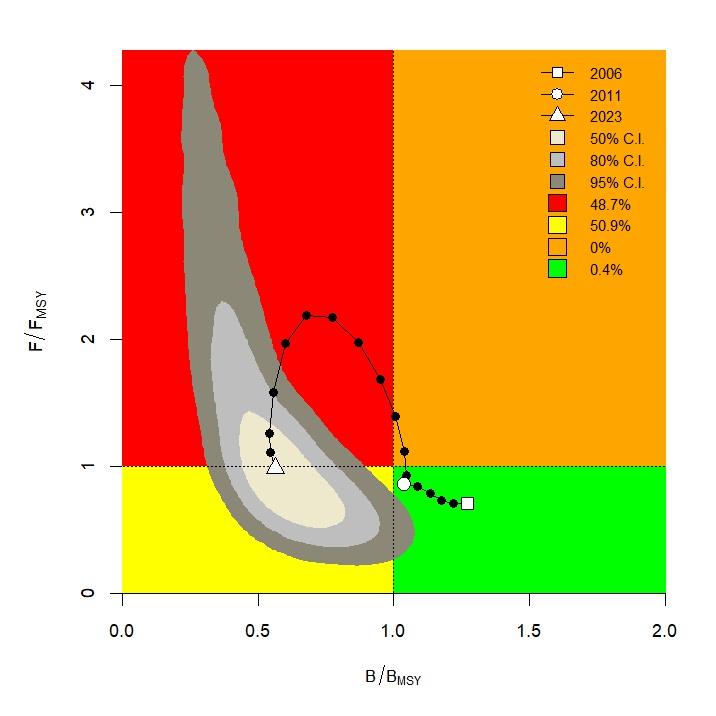 | h) | 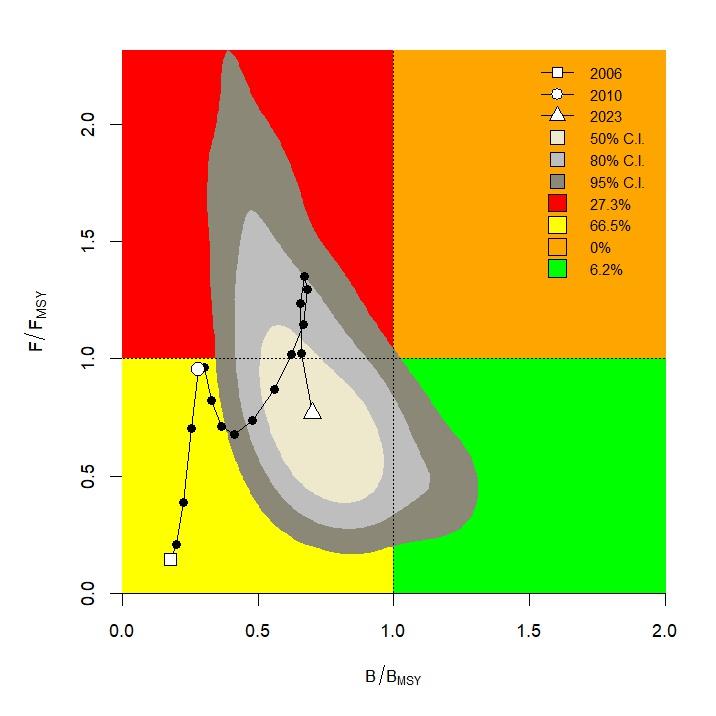 |
| i) | 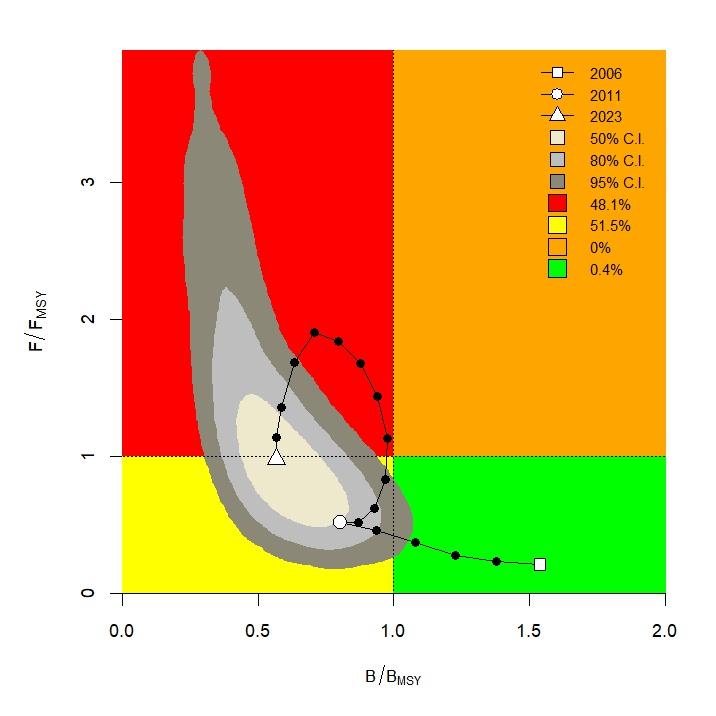 | j) | 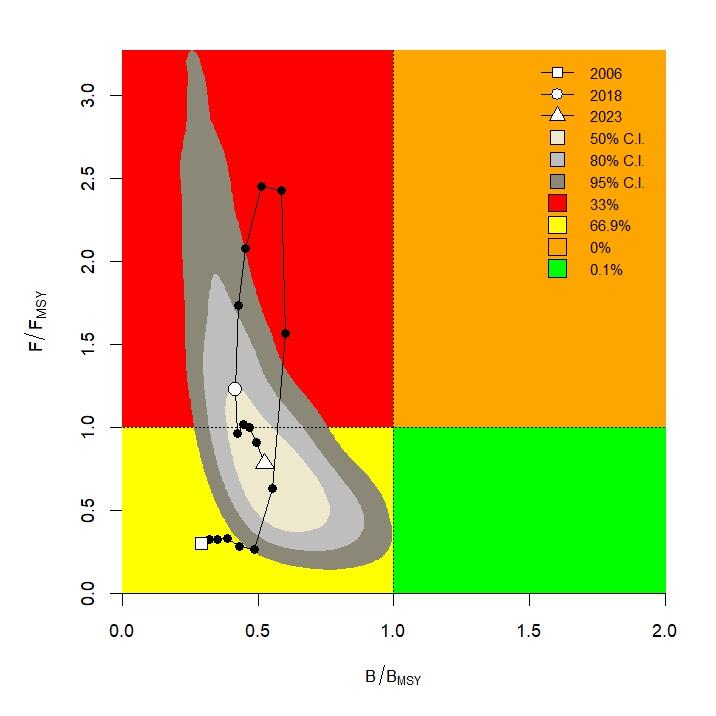 |

**Figure S6**: **Kobe diagrams based on the relationship between biomass (B) and fishing pressure (F) relative to maximum sustainable yield (MSY), illustrating the status of the stocks in group 3 (not over-fished under the Flexible approach) in the Cortez province from 2006 to 2023**. a) *Calamus spp*., b) *Caulolatilus princeps*, c) *Diapterus spp*., d) *Eucinostomus spp*., e) *Eugerres lineatus*, f) *Haemulon spp*., g) *Mugil spp*. COR, h) *Synodus spp*., i) *Trachinotus paitensis*, and j) *Trachinotus rhodopus*. Each point represents a year, and the color legend indicates the probability that the most recent year falls into one of the following states: unsustainable (red), overfishing (orange), overexploited (yellow), and sustainable (green). The ellipse represents uncertainty for the most recent year with confidence intervals of 50% in beige, 80% in gray, and 95% in dark gray.

| a) | 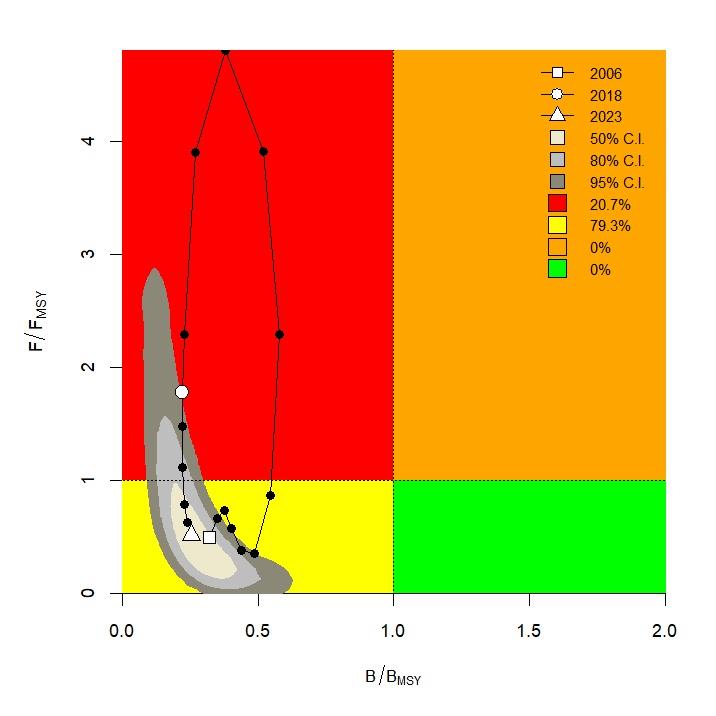 | b) | 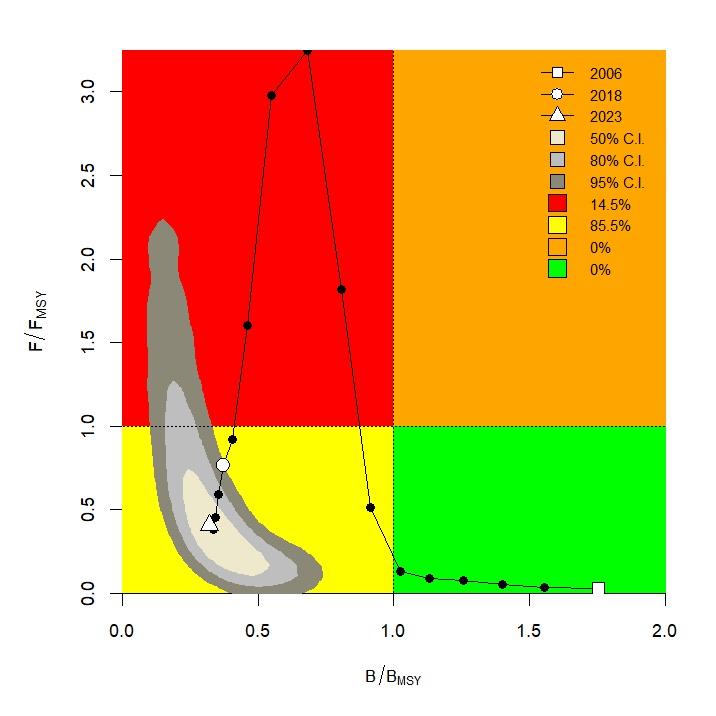 |
| --- | --- | --- | --- |
| c) | 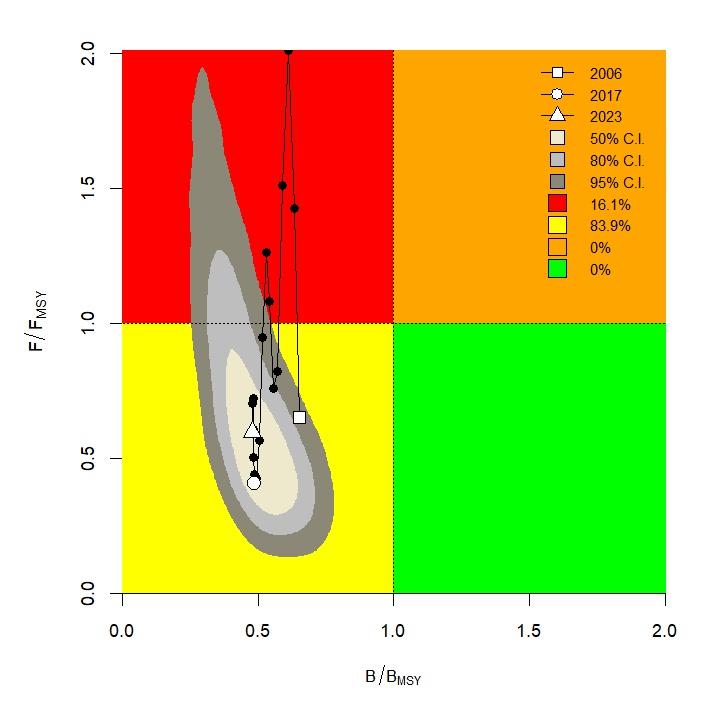 | d) | 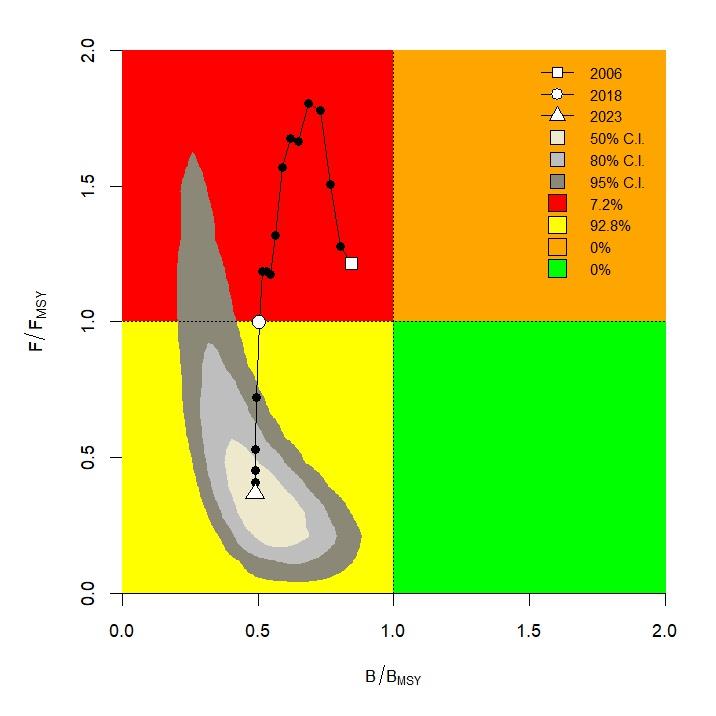 |
| e) | 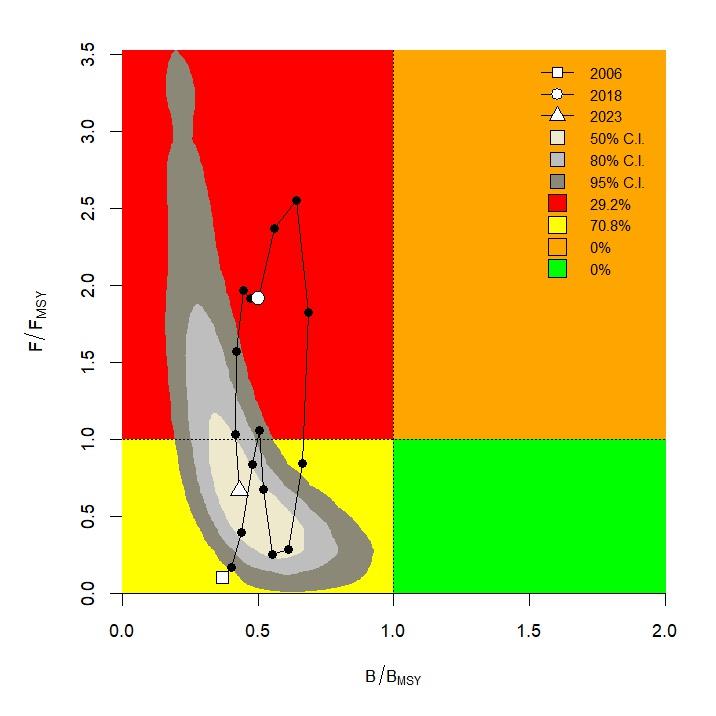 | f) | 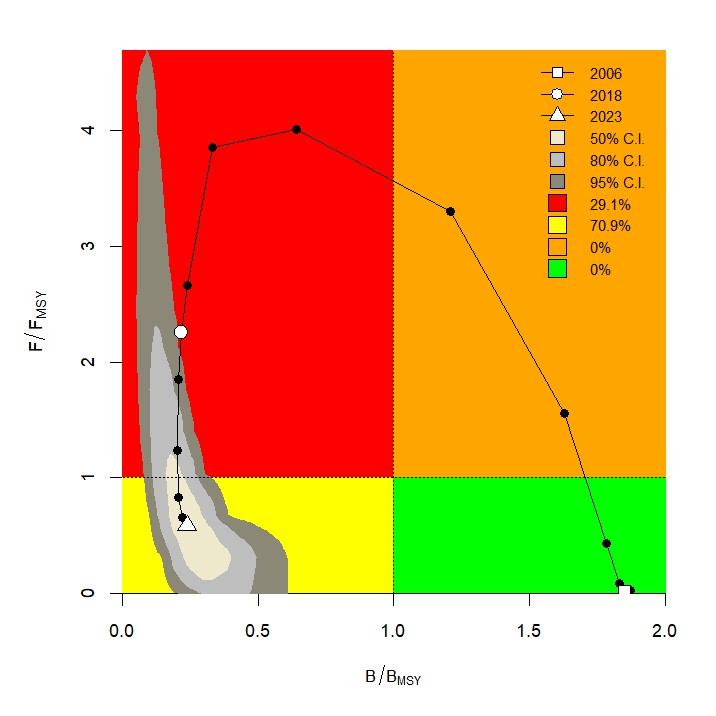 |
| g) | 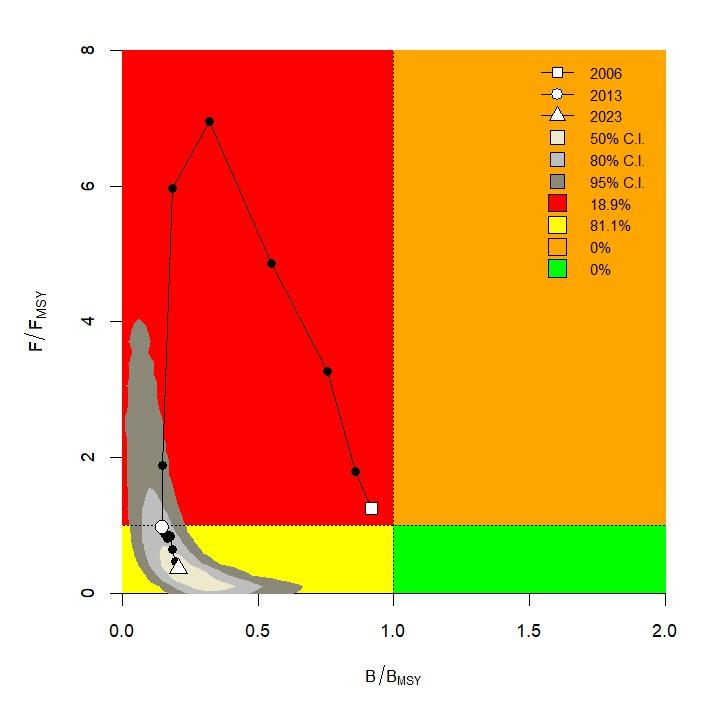 | h) | 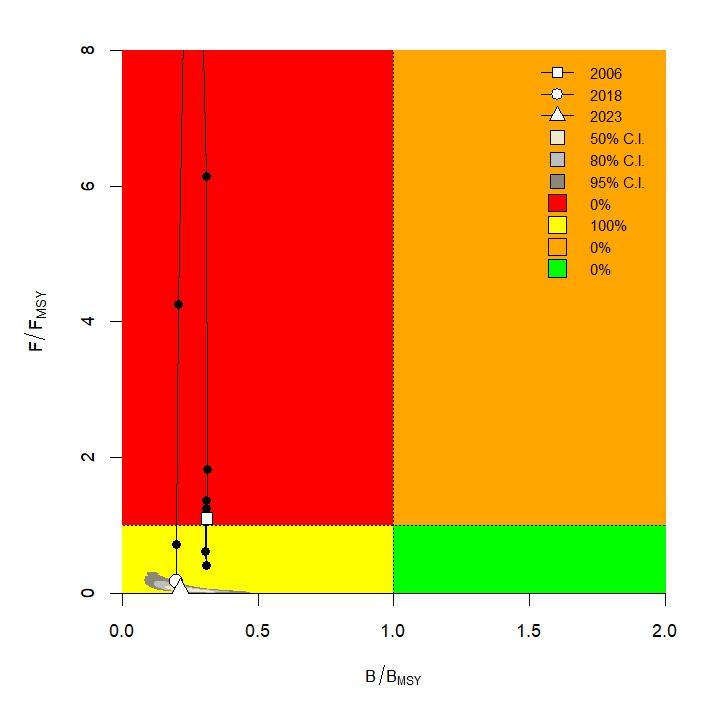 |
| i) | 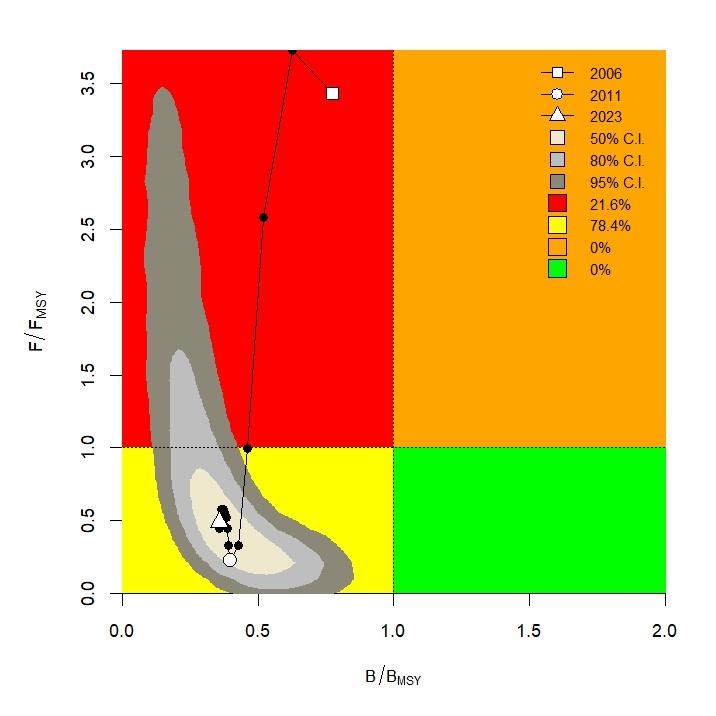 | j) | 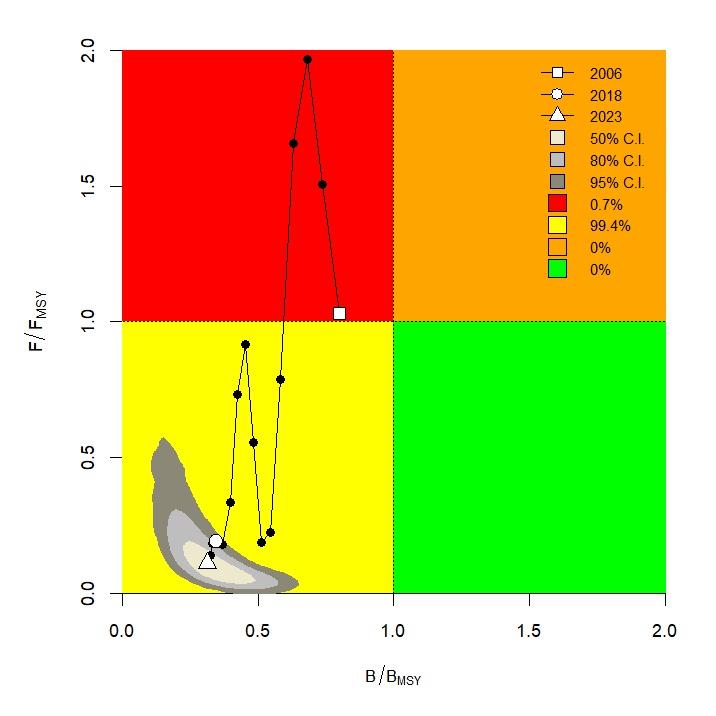 |
| k) | 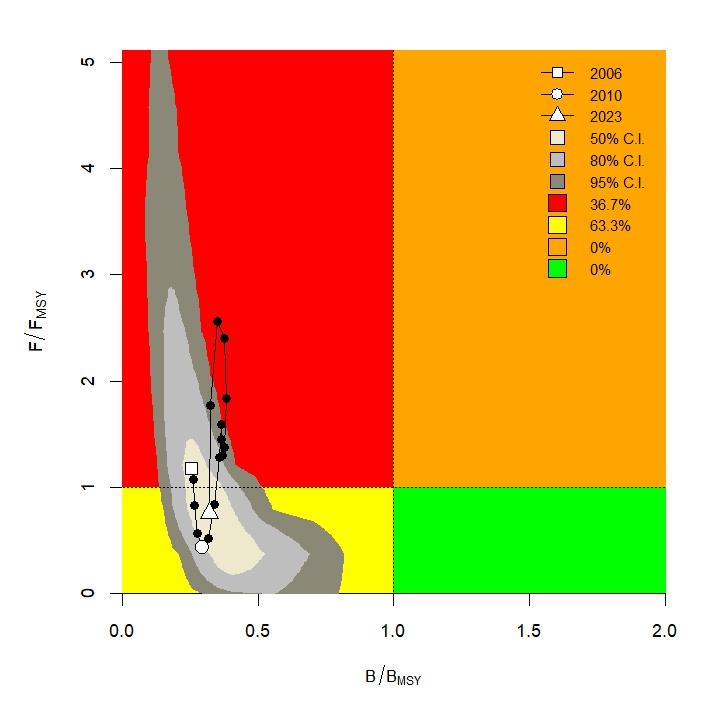 | l) | 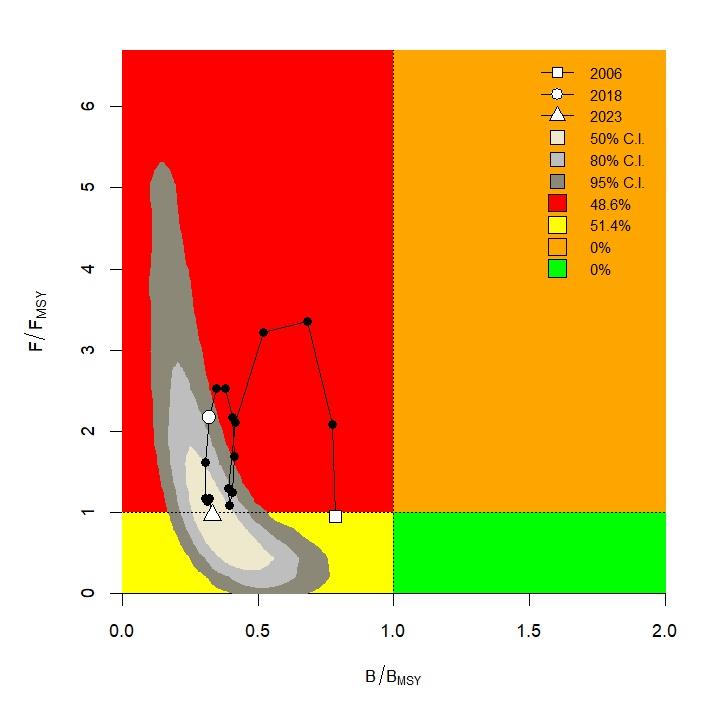 |
| m) | 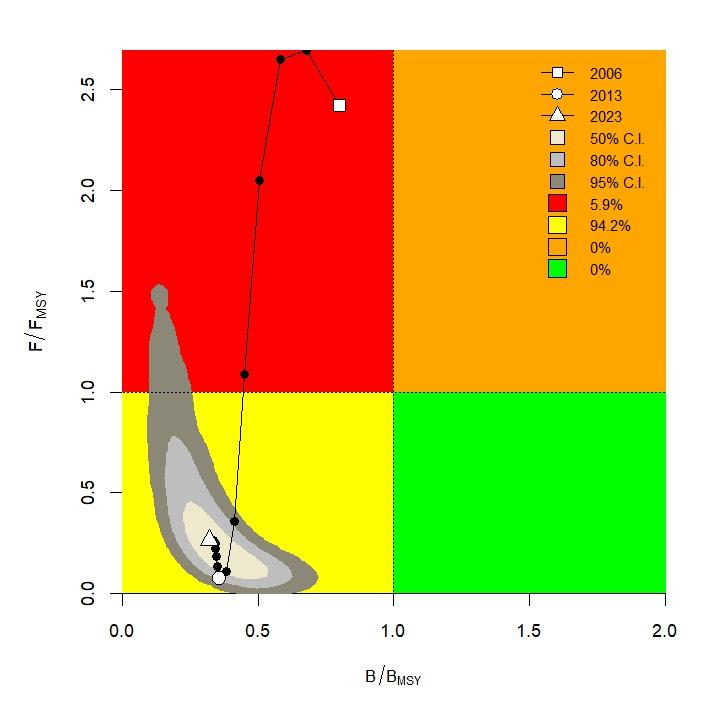 | n) | 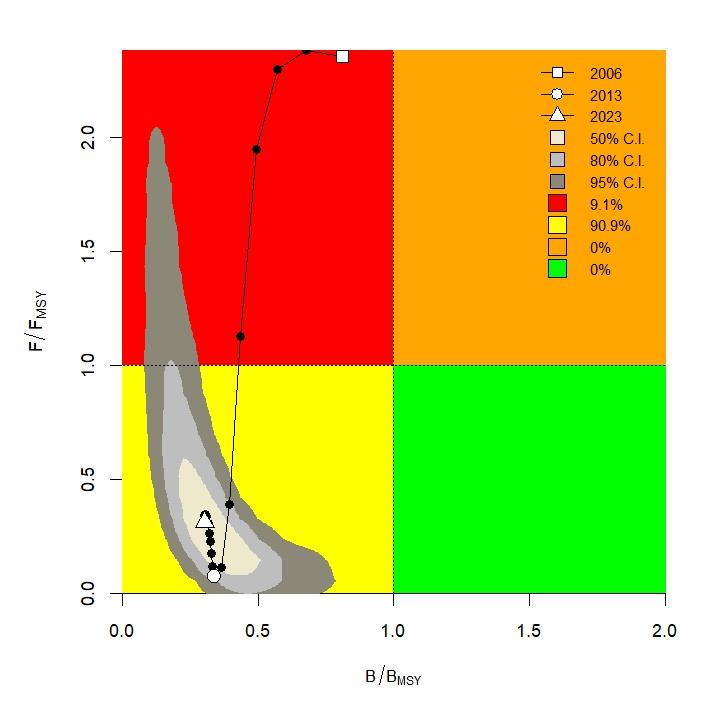 |
| o) | 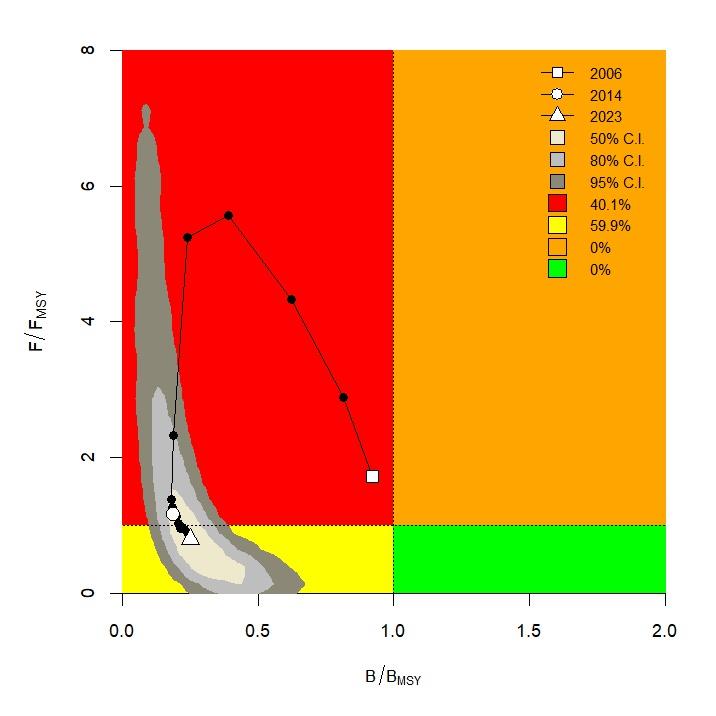 | p) | 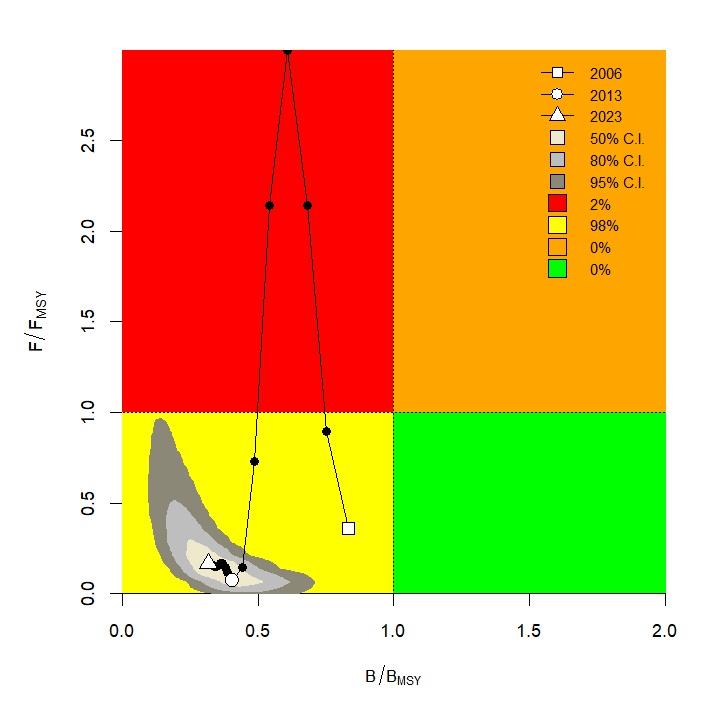 |
| q) | 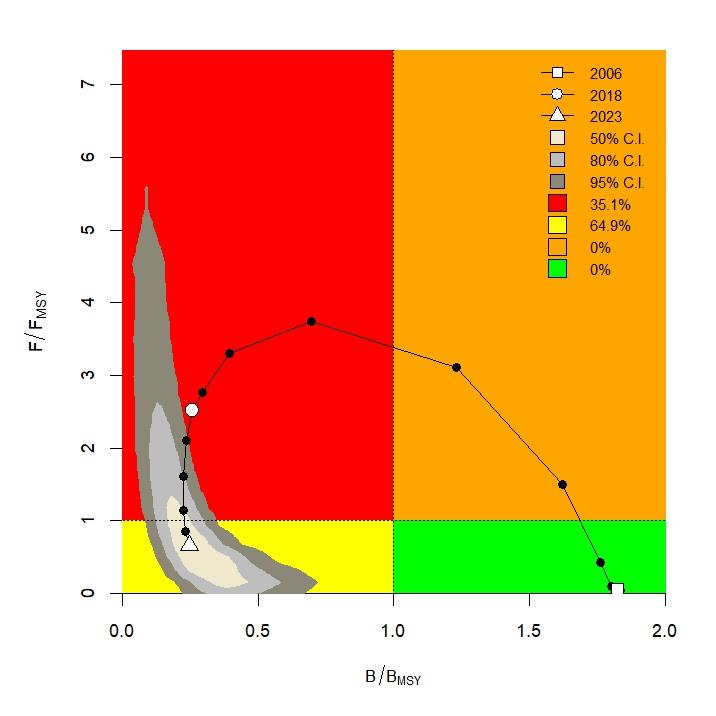 | r) | 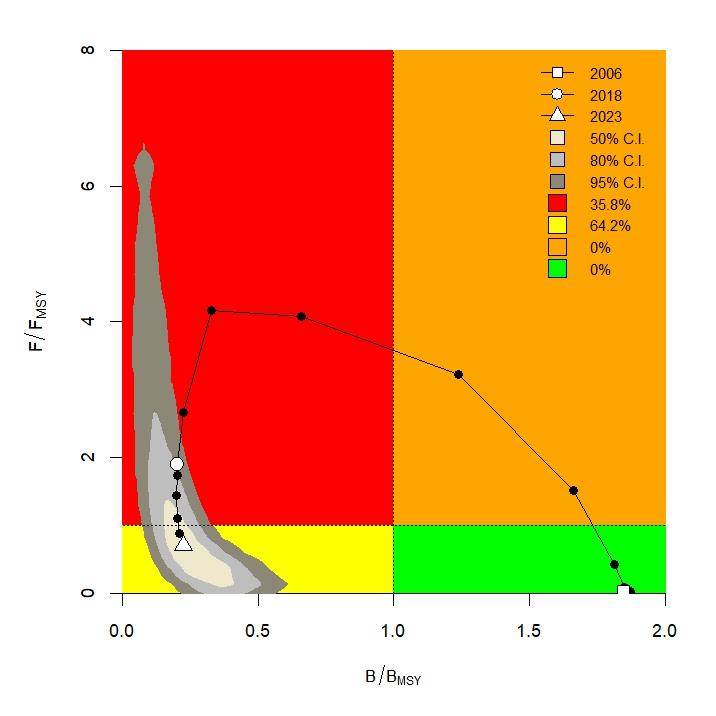 |
| s) | 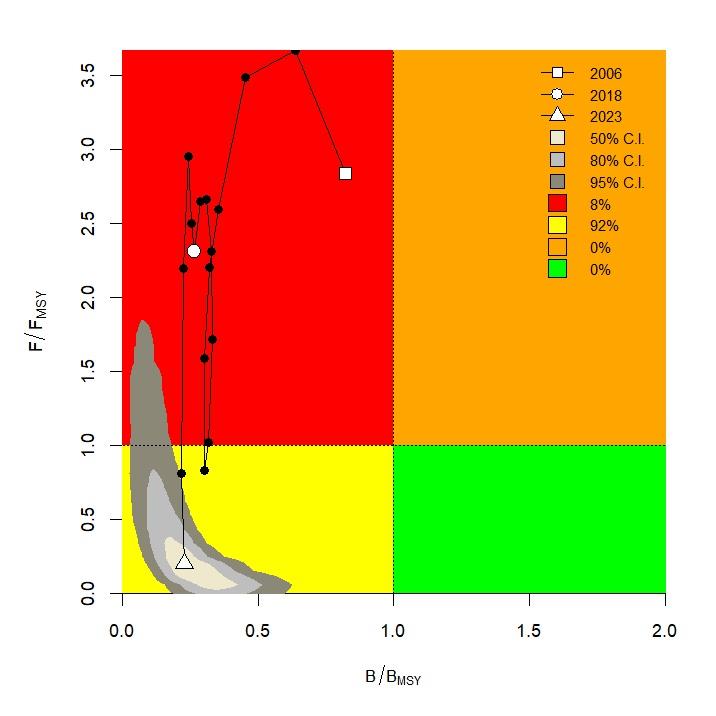 | t) | 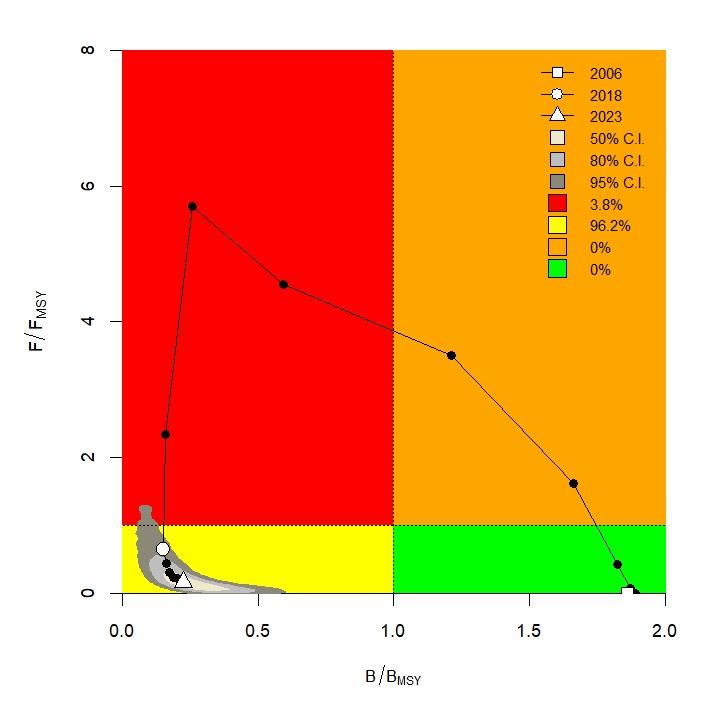 |
| u) | 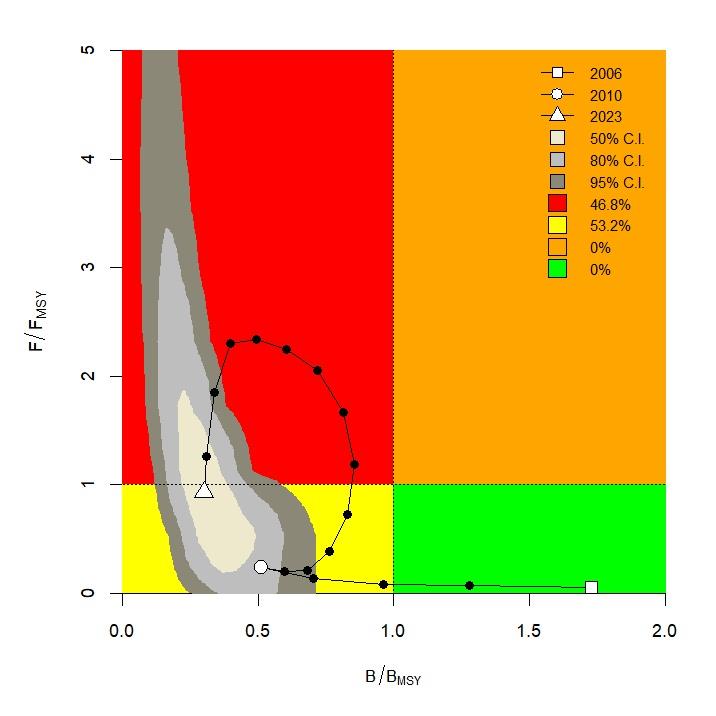 | v) | 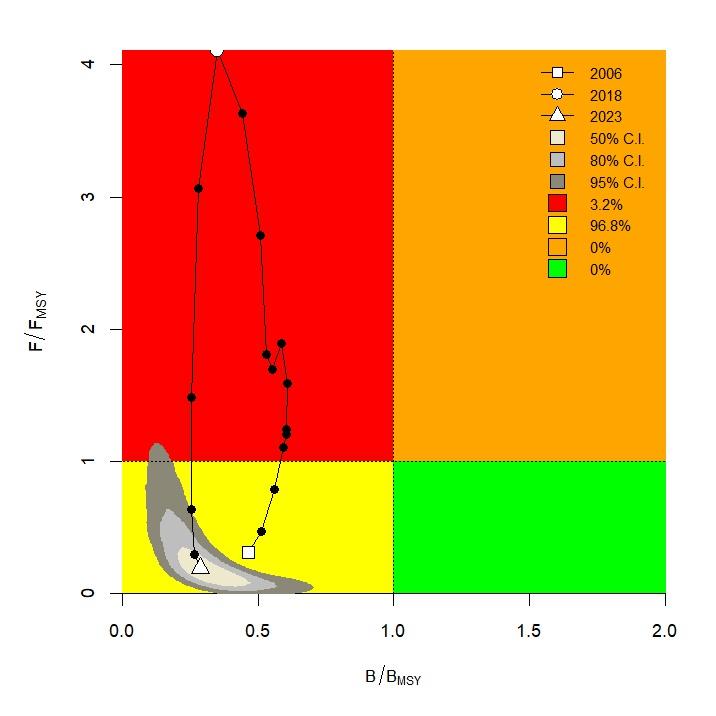 |
| w) | 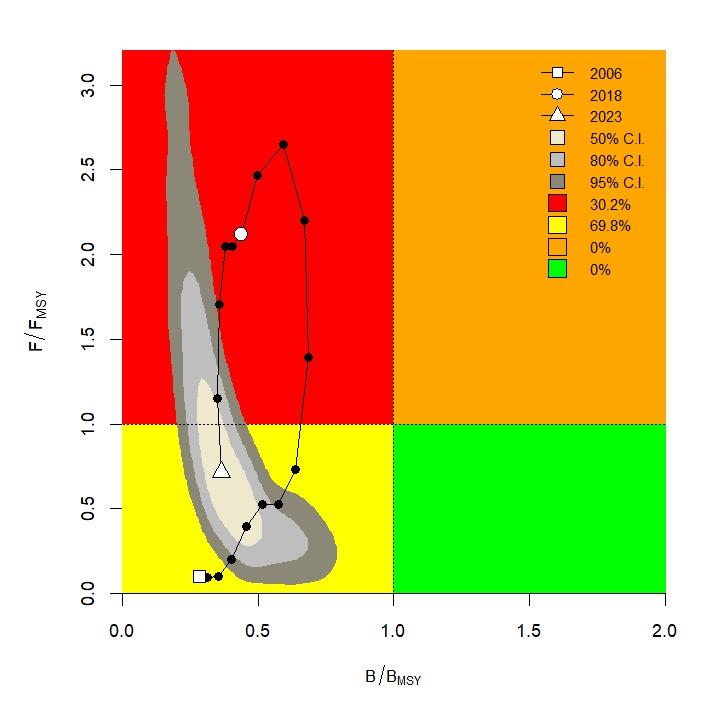 | x) | 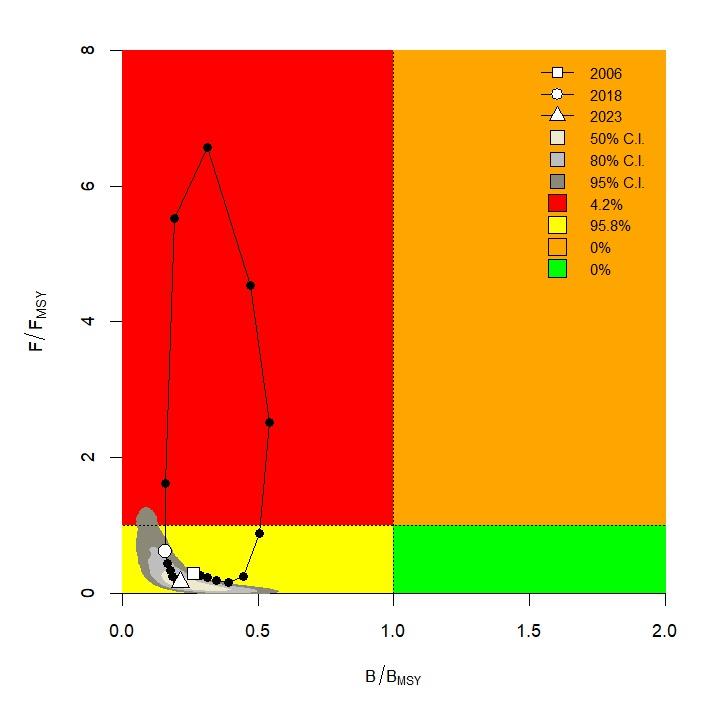 |
| y) | 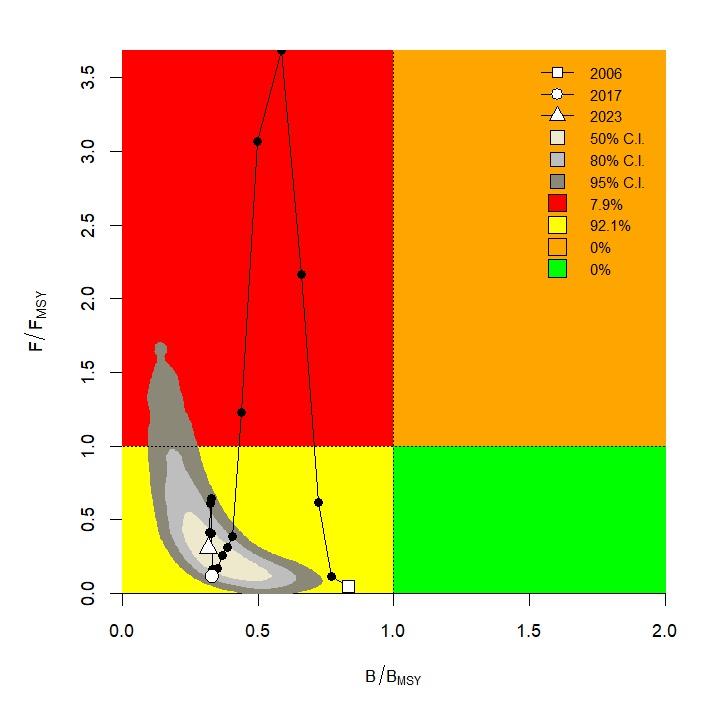 | z) | 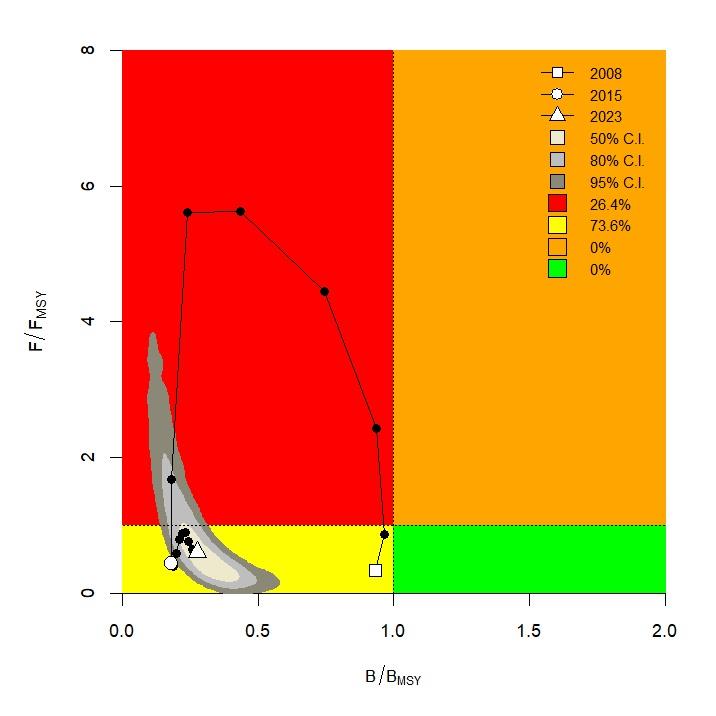 |
| aa) | 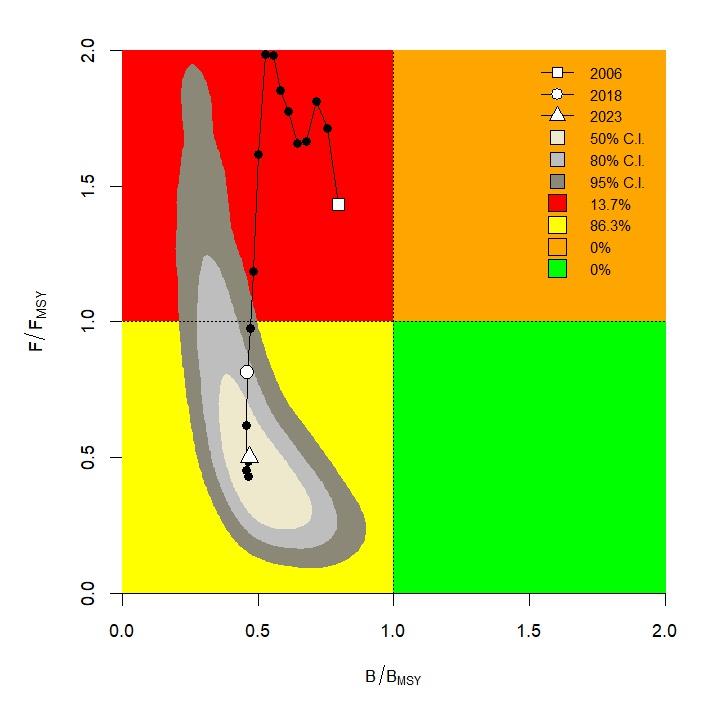 | ab) | 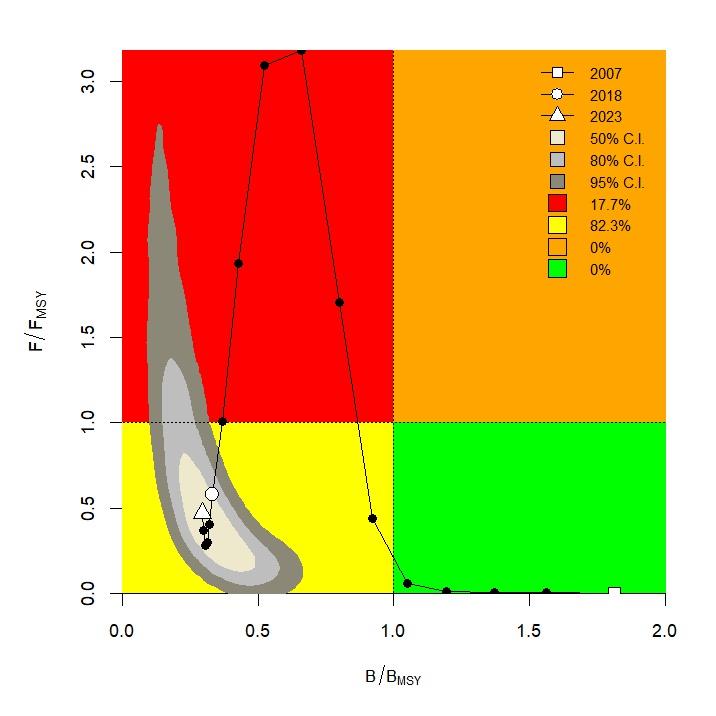 |
| ac) | 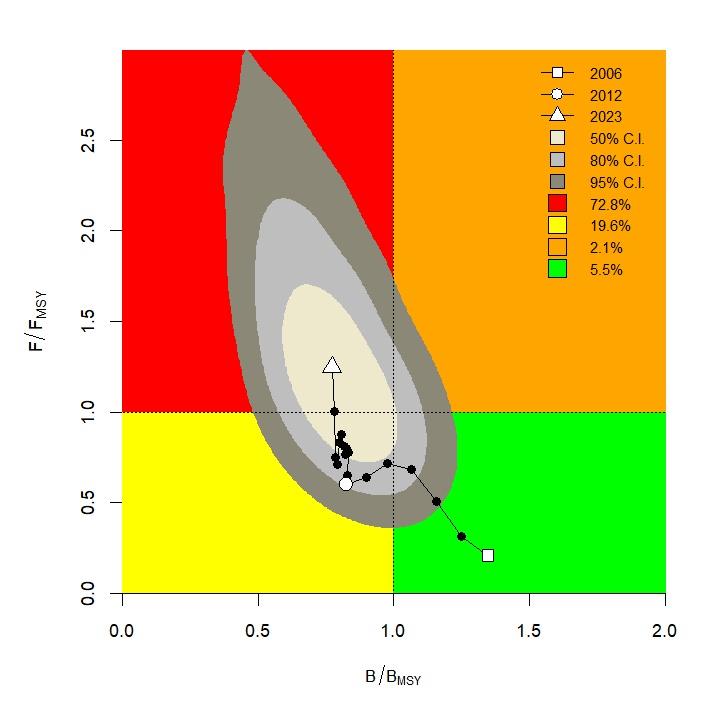 | ad) | 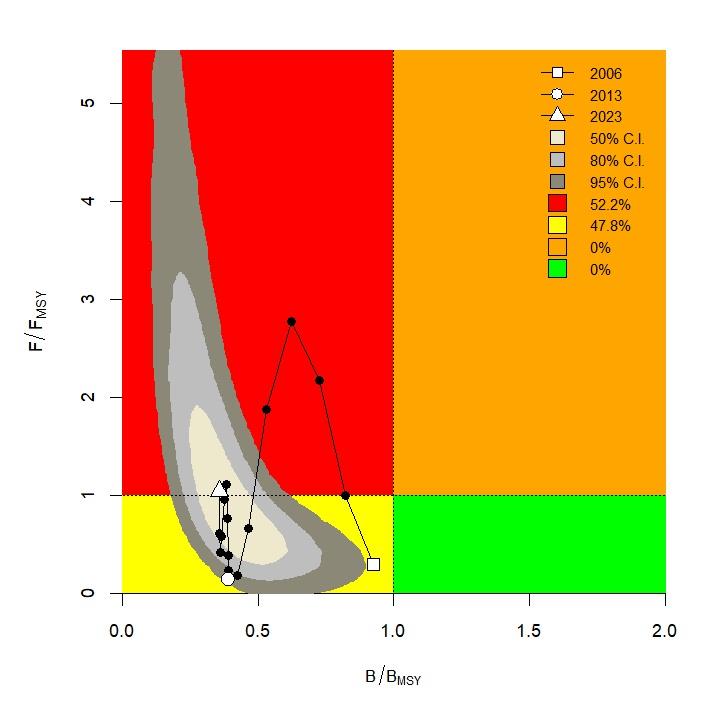 |
| ae) | 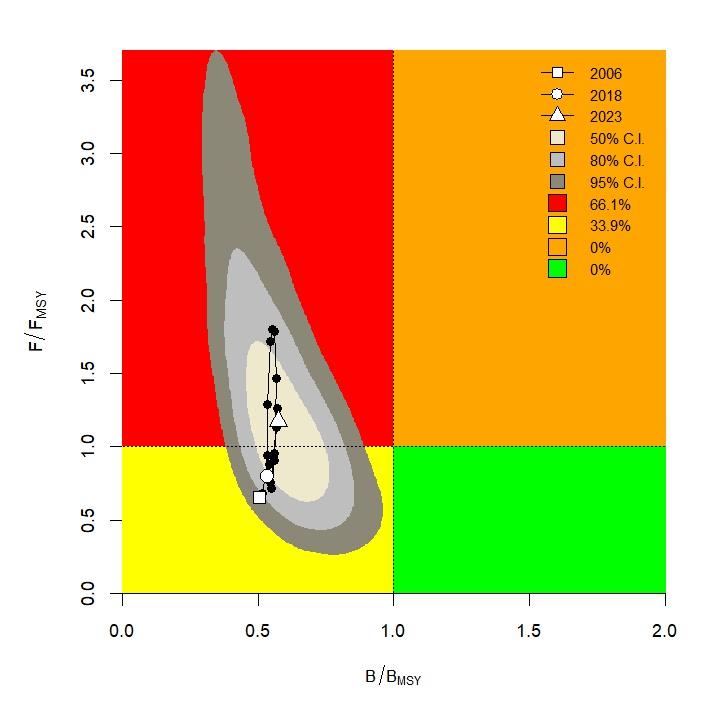 | af) | 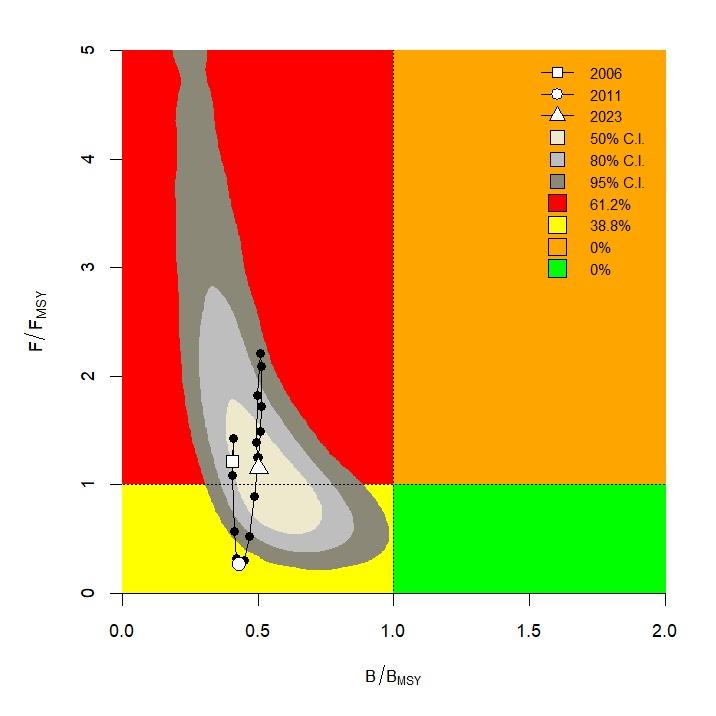 |
| ag) | 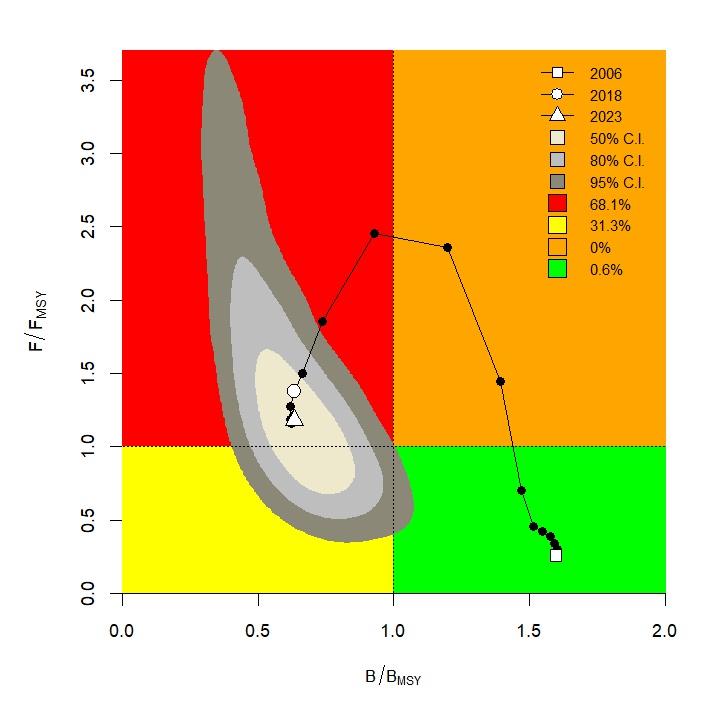 | ah) | 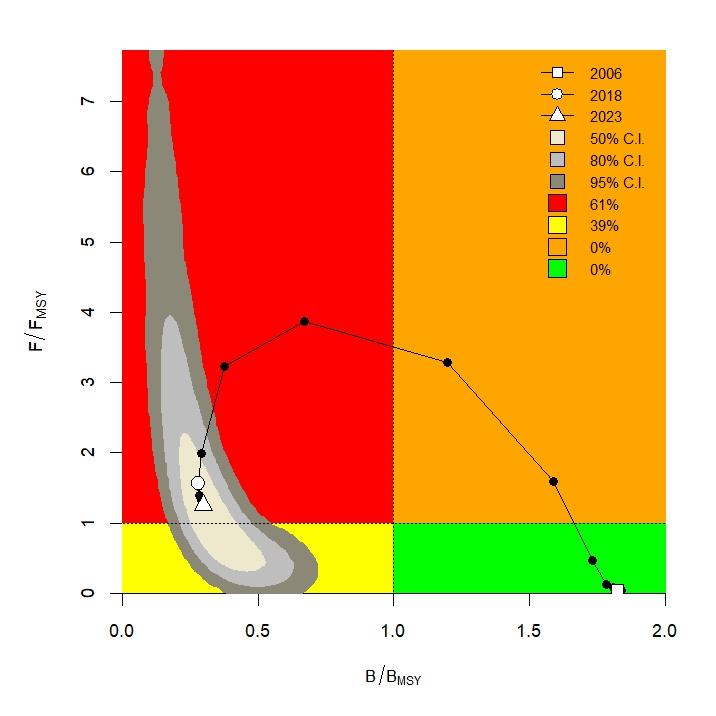 |
| ai) | 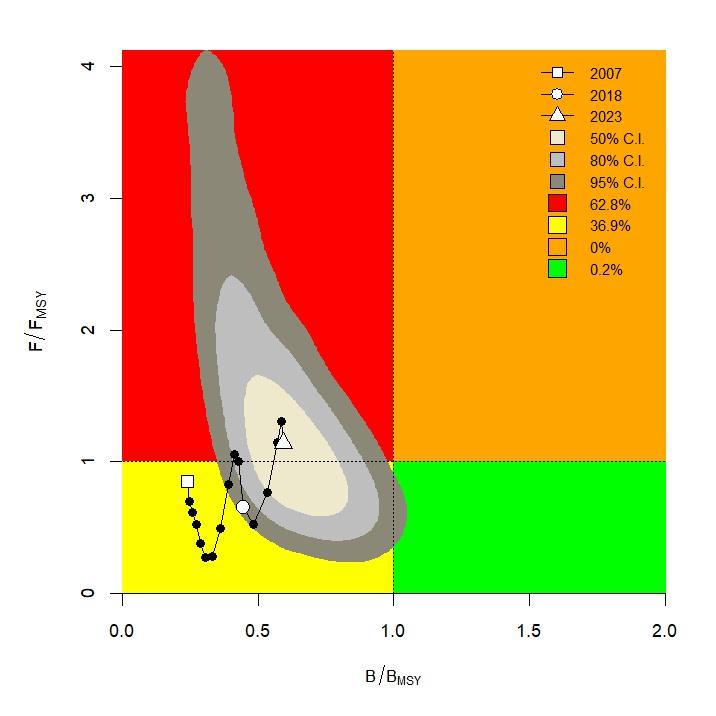 | aj) | 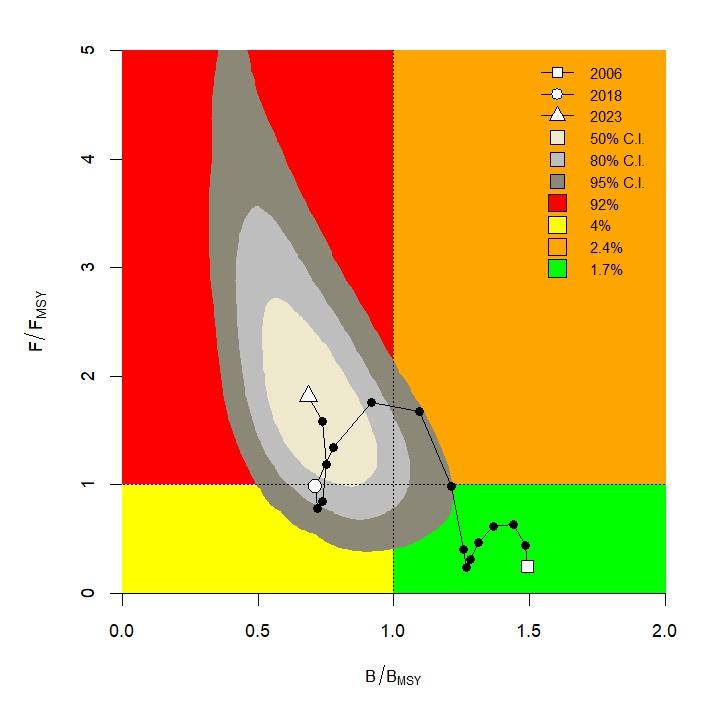 |
| ak) | 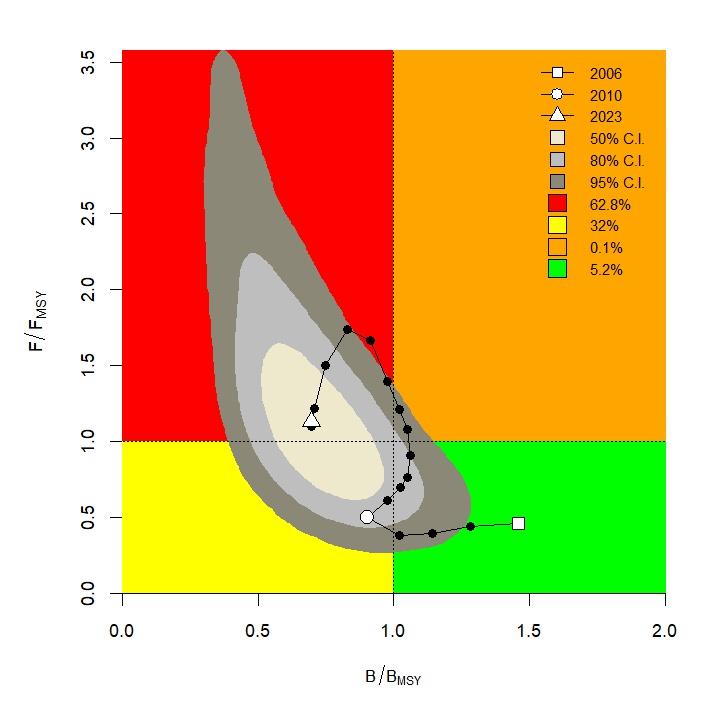 | al) |  |
| am) |  | an) |  |
| ao) |  | ap) |  |
| aq) |  | ar) |  |
| as) |  | at) |  |
| au) |  | av) |  |

**Figure S7**: **Kobe diagrams based on the relationship between biomass (B) and fishing pressure (F) relative to maximum sustainable yield (MSY), illustrating the status of the stocks in group 4 (unsustainable under all the approaches) in the Cortez province from 2006 to 2023**. a) *Bagre panamensis*, b) *Balistes polylepis*, c) *Bodianus spp*., d) *Caulolatilus affinis*, e) *Centropomus medius*, f) *Cephalopholis colonus*, g) *Synodus spp*., h) *Trachinotus paitensis*, i) *Trachinotus rhodopus*, j) *Hyporthodus acanthistius*, k) *Kathetostoma averruncus*, l) *Kyphosus spp*., m) *Lutjanus argentiventris*, n) *Lutjanus colorado*, o) *Lutjanus guttatus*, p) *Lutjanus novemfasciatus*, q) *Lutjanus peru*, r) *Menticirrhus spp*., s) *Microlepidotus inornatus*, t) *Micropogonias megalops*, u) *Mugil curema*, v) *Mulloidicthys dentatus*, w) *Nematistius pectoralis*, x) *Paralabrax nebulifer*, y) *Paralabrax spp*., z) *Scarus spp*. COR, aa) *Scorpaena mystes*, ab) *Seriola spp*., ac) *Anisotremus interruptus*, ad) *Atractoscion nobilis*, ae) *Bagre pinnimaculatus*, af) *Chanos chanos*, ag) *Cyclopsetta spp*., ah) *Diplectrum euryplectrum*, ai) *Elops affinis*, aj) *Epinephelus spp*. COR, ak) *Hyporthodus niphobles*, al) *Lutjanus spp*., am) *Menticirrhus elongatus*, an) *Mugil setosus*, ao) *Mycteroperca prionura*, ap) *Scarus perrico*, aq) *Sebastes spp*., ar) *Selar crumenophtalmus*, as*) Selene brevoortii*, at) *Sphoeroides annulatus*, au) *Stereolepis gigas,* and av) *Trachinotus spp*. Each point represents a year, and the color legend indicates the probability that the most recent year falls into one of the following states: unsustainable (red), overfishing (orange), overexploitation (yellow), and sustainable (green). The ellipse represents uncertainty in the last year with confidence intervals of 50% in beige, 80% in gray, and 95% in dark gray.
